# Causalized convergent cross-mapping and its approximate equivalence with directed information in causality analysis

**DOI:** 10.1093/pnasnexus/pgad422

**Published:** 2023-12-07

**Authors:** Jinxian Deng, Boxin Sun, Norman Scheel, Alina B Renli, David C Zhu, Dajiang Zhu, Jian Ren, Tongtong Li, Rong Zhang

**Affiliations:** Department of Electrical and Computer Engineering, Michigan State University, East Lansing, MI 48824, USA; Department of Electrical and Computer Engineering, Michigan State University, East Lansing, MI 48824, USA; Department of Radiology, Michigan State University, East Lansing, MI 48824, USA; Department of Neuroscience, Michigan State University, East Lansing, MI 48824, USA; Department of Radiology, Michigan State University, East Lansing, MI 48824, USA; Department of Computer Science and Engineering, University of Texas, Arlington, TX 76010, USA; Department of Electrical and Computer Engineering, Michigan State University, East Lansing, MI 48824, USA; Department of Electrical and Computer Engineering, Michigan State University, East Lansing, MI 48824, USA; Institute for Exercise and Environmental Medicine, Texas Health Presbyterian Hospital Dallas, Dallas, TX 75231, USA; Departments of Neurology and Internal Medicine, University of Texas Southwestern Medical Center, Dallas, TX 75390, USA

## Abstract

Convergent cross-mapping (CCM) has attracted increased attention recently due to its capability to detect causality in nonseparable systems under deterministic settings, which may not be covered by the traditional Granger causality. From an information-theoretic perspective, causality is often characterized as the directed information (DI) flowing from one side to the other. As information is essentially nondeterministic, a natural question is: does CCM measure DI flow? Here, we first causalize CCM so that it aligns with the presumption in causality analysis—the future values of one process cannot influence the past of the other, and then establish and validate the approximate equivalence of causalized CCM (cCCM) and DI under Gaussian variables through both theoretical derivations and fMRI-based brain network causality analysis. Our simulation result indicates that, in general, cCCM tends to be more robust than DI in causality detection. The underlying argument is that DI relies heavily on probability estimation, which is sensitive to data size as well as digitization procedures; cCCM, on the other hand, gets around this problem through geometric cross-mapping between the manifolds involved. Overall, our analysis demonstrates that cross-mapping provides an alternative way to evaluate DI and is potentially an effective technique for identifying both linear and nonlinear causal coupling in brain neural networks and other settings, either random or deterministic, or both.

**Significance Statement**

Causality analysis aims to find the relationship between causes and effects and is a central topic in science, economy, climate, and many other fields. Causality can be characterized using the directed information (DI) flowing from one side to the other. However, relying on probability estimation, DI may be very sensitive to data size and the quantization procedures used. Convergent cross-mapping (CCM), on the other hand, gets around this problem through geometric cross-mapping between the systems and random variables involved. In this paper, by establishing the approximate equivalence between causalized CCM and DI, we showed that causalized CCM provides an effective model-free approach to measure causal coupling in deterministic, random, or hybrid settings and can benefit a broad spectrum of applications that require quantitative causality detection.

## Introduction

Causality analysis aims to find the relationship between causes and effects and has been a central topic in science, economy, climate, and many other fields (1–7). The first practical causality analysis framework is Granger causality (GC), which was proposed by Granger in 1969 (8). GC is a *statistical* approach that relies on a multistep linear prediction model, where the basic idea is to determine whether the values of one time series are useful in predicting the future values of the other. As a well-known technique, the validity and computational simplicity of GC have been widely recognized (9–12), and its nonlinear extensions have also been studied in the literature (13–16). It has been observed by Granger himself (8), as well as others (17), that GC requires separability between the variables under consideration and may be problematic in detecting causation in deterministic settings.

As an effort to address this problem, in 2012, Sugihara et al. (17) proposed to use the convergent cross-mapping (CCM) approach and demonstrated that CCM could serve as an effective tool in addressing nonseparable systems and identifying weakly coupled variables under deterministic settings, which may not be covered by GC. Since then, CCM has attracted considerable attention from the research community in many different fields (18–25).

Rooted in dynamic systems theory, the fundamental assumption of CCM is that the dynamics in the world are not completely random but governed by some underlying deterministic rules. In dynamic systems theory, an attractor is a set of states toward which a system tends to evolve from a wide variety of starting conditions (26). In finite-dimensional systems, each evolving variable could be represented as a *d*-dimensional vector. The attractor is then a region in the *d*-dimensional space and is generally represented as a manifold. CCM relies on Takens’ embedding theorem (27), which says that in general, the attractor manifold of a dynamical system can be “reconstructed” from a single observation variable of the system, say *X*, in the sense that the reconstructed attractor (called a shadow manifold) $M_{x}$ is diffeomorphic to the true manifold, M. Based on Takens’ theorem, if two variables *X* and *Y* are causally linked, then they share the same attractor manifold M, and their corresponding shadow manifolds $M_{x}$ and $M_{y}$ will also be diffeomorphic. Consequently, nearby time points on manifold $M_{x}$ will be mapped to nearby points on $M_{y}$. That is, the time indices of nearby points in $M_{x}$ can be used to identify the nearby points in $M_{y}$. Therefore, the current state of variable *Y* can be predicted based on *X* and vice versa. If we denote the predicted version of *Y* as $\hat{Y},\;$ the CCM causation from *X* to *Y* is defined as the Pearson correlation between *Y* and $\hat{Y}$. The CCM algorithm is summarized in Box 1, and the concept of cross-mapping is illustrated through figures in Section 1 of Supplementary material.

Box 1. **CCM and causalized CCM.**

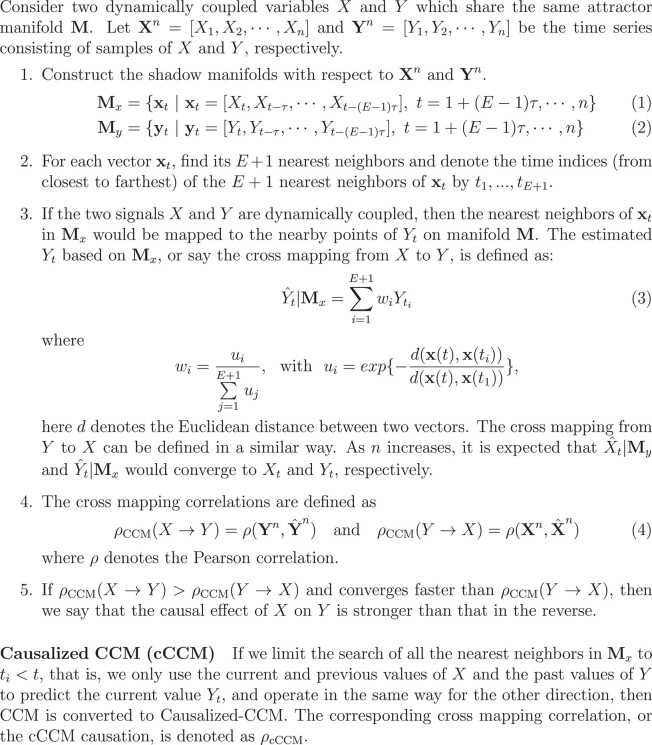

In literature, model-free methods like CCM, which rely on the embedding theorem and allow the reconstruction of the system space from scalar observations, are collectively referred to as state-space reconstruction approaches (28–34). They generally use nearest neighbor search and cross-mapping in the shadow manifolds to predict or reconstruct the state of variables, but the prediction methods and the measures used to characterize unidirectional causal coupling may vary in different approaches (17, 29, 33). A notable feature of Sugihara's approach (17) is that when *X* and *Y* are dynamically coupled, the prediction of *X* based on *Y* (and vice versa) will converge to the true value itself as the data length increases and was therefore named CCM. For this reason, CCM turns out to be a popular and representative technique among the state-space reconstruction methods. Along the same line, Porta et al. (29, 30) developed a multivariate *K*-nearest-neighbor (KNN) search-based predictability approach for causality detection in systems with multiple dynamically coupled time series by taking the impact of conditioning variables into consideration. As will be seen in Results section, this approach provides a possible way to extend bivariate CCM (17) to multivariate conditional CCM.

Note that since the late 1960s, several causality analysis frameworks have been proposed from different perspectives. In addition to GC and CCM, other representative frameworks include Directed Information (DI, 1990) (35) and Transfer Entropy (TE, 2000) (36), which were both developed based on information theory, and dynamic causal modeling (DCM, 2003) (37), which is rooted in the classical control theory and the neural mass model.

To this end, some natural and fundamental questions are: (i) Would CCM still be effective in random settings? (ii) Does CCM measure DI flow which is essentially nondeterministic? (iii) As a relatively recent newcomer to the family, what is the relationship of CCM with existing causality detection frameworks?

To answer these questions, we need to take a closer look at existing causality detection tools. DI was proposed by Massey in 1990 (35) when studying discrete memoryless communication channels with feedback, and it is the first causality detection tool based on information theory. DI measures the directed information flowing from one sequence to the other. As an information-theoretic framework, a major advantage of DI is that it is a universal method that does not rely on any model assumptions of the signals and is not limited by linearity or separability (38, 39).

TE is another information-theoretic causation measurement. It was introduced by Schreiber in 2000 (36) and measures the decrease of entropy in one signal *Y* after another signal *X* has been observed. Like GC, TE measures how much additional information the past values of *X* contain about the future observations of *Y*, given that we already knew the past values of *Y*. It can be regarded as a direct generalization of GC based on information theory.

Let $X_{i}$ and $Y_{i}$ represent the *i*-th sample of *X* and *Y*, respectively, generally taken at the same time instants. The major difference between DI and TE is that—TE only considers the impact of the past values of *X* (i.e. all samples *X_k_* with *k < i*) on *Y_i_*, just as in GC, while DI not only counts the impact of the past values of *X,* but also takes the instantaneous information exchange between *X_i_* and *Y_i_* into consideration (40). When there is no (significant) instantaneous information exchange between *X_i_* and *Y_i_*, as in the case when the information transmission from *X* to *Y* takes *nonzero* time, then DI and the cumulative variant of TE are essentially equivalent on the calculation of *DI flow* from one variable to the other. Moreover, Barnett et al. (41) proved that GC and TE are equivalent under the auto-regression model and Gaussian variables. This implies that, as model-free causality measures, both DI and TE are conditionally equivalent to GC under the auto-regression model and Gaussian variables. In literature, all the three frameworks have been applied for causality detection in neuroscience (5, 42, 43).

The DCM framework was proposed by Friston et al. in 2003 (37). It relies on the neural mass model and takes a similar format as the dynamic state-space model in the classical control theory. DCM provides a flexible framework to characterize the connectivity or coupling between brain regions and how the coupling is influenced by external inputs and the environment. Relying on the expectation maximization (EM) algorithm, DCM has been implemented on both fMRI and electroencephalogram data (44–47). In practical applications, due to the computational complexity, DCM is usually used as a confirmatory approach. That is, the users need to put forward different connectivity models and then compare them based on their likelihood evaluated under DCM, the process is known as Bayesian modeling (45). In (48), we explored the relationship between DI and DCM based on fMRI data and showed that: discretized DCM *and* DI *are equivalent* in characterizing the causal relationship between two brain regions under Gaussian variables when the external input is approximately a constant (i.e. when the external input changes much slower than the neuronal activity).

Based on the discussions above, we can see that as a universal causality measure, DI serves as the pivot that links GC, TE, and DCM together through the conditional equivalence between them. From the information theory perspective, DI demonstrates that causality can be quantified using the directed information flow from one time series to the other. Therefore, if CCM can be linked with DI, it is then connected with the whole causality analysis family and obtains its physical meaning in terms of directed information transmission.

Before doing that, one prestep needs to be taken. Recall that in its original definition, causality aims to determine whether the current and past values of one time series are useful in predicting the future values of the other in addition to its own past values. In the existing CCM algorithm (Box 1), however, the *whole* time series corresponding to *X*, and both the past and future values of *Y* are *all* exploited to estimate the current value of *Y*. That is, the causality defined in CCM is inconsistent with the original, widely accepted definition of causality, and changes are therefore needed to fill in the gap.

In this paper, first, we causalize the CCM algorithm so that only the current and previous values of variable *X* and the past values of variable *Y* are used to predict the current value of *Y*, and vice versa. The cCCM aligns with the traditional definition of causality, in the sense that the future values of one process cannot influence the past of the other.

Second, we demonstrate the approximate equivalence of CCM and cCCM through various simulation examples, including Gaussian random processes, sinusoidal waveforms, autoregressive processes, stochastic processes with a dominant frequency component embedded in noise, deterministic chaotic maps, and systems with memory. In all these examples, cCCM and CCM are highly consistent in causality detection and show similar convergence speeds in cross-mapping. Also, both cCCM and CCM can detect the increase in coupling strength and show consistent sensitivity to the changes in coupling strength. We also explore the connections and differences between CCM causation and Pearson correlation. The simulation result indicates that high correlation might lead to high bidirectional causation, but low correlation may correspond to either high or low causation, and either unidirectional or bidirectional.

Third, based on Takens’ theorem (27) and Gel’fand's theorem on the conditional equivalence between Pearson correlation and mutual information (MI) (49), as well as the Shannon–McMillan–Breiman theorem (50), we show that—under the assumption that the future of one process cannot influence the past or current of the other, cCCM and DI are approximately equivalent under Gaussian variables. An approximate mathematical relationship between cCCM and DI is derived, and the theoretical result is demonstrated using experimental fMRI data. Relying on information theory, this result reveals how cCCM measures the directed information flow from one time series to the other and links cCCM with other members of the causality detection family.

We also compare the performance of DI and cCCM through simulation examples where the time series may not (both) be Gaussian random variables. It was observed that in general, cCCM tends to be more robust or sensitive than DI in causality detection. This is largely because DI relies heavily on probability estimation, which is sensitive to data size as well as the digitization or quantization process used in the implementation algorithm. cCCM, on the other hand, gets around this problem through geometric cross-mapping of the corresponding neighborhoods between the manifolds involved. As exhaustive KNN search is required at the prediction of every sample of the target time series, the disadvantage of cCCM, therefore, is its high computational complexity, which is $O(n^{2})$ in the sequence length n, while the computational complexity of DI is only O(n). Moreover, we also investigate the noise tolerance of cCCM and show that—in the presence of noise, the cCCM causation may decrease, but would be very close to the noise-free case when the signal-to-noise ratio (SNR) is reasonably high (≥15 dB).

Finally, we demonstrate the capability of cCCM in detecting unidirectional causality through task-driven fMRI data. It is shown that unidirectional causality among the regions of interest (ROIs) can be detected by cCCM, and the result is consistent with that of CCM and DI and is also consistent across the majority of subjects. We also conduct multivariate conditional CCM and cCCM for all the ROI pairs. Our results indicate that due to rich diversity in the brain network, multivariate conditional CCM and cCCM with respect to (i.e. conditioning on) all the rest of ROI regions generally result in very small causality ratios and cannot be used for causality detection. However, conditional cCCM and CCM with respect to individual regions can detect unidirectional causality and demonstrate the impact of interdependence between the ROI regions on the causality ratio.

Overall, our analysis demonstrates that CCM provides an innovative and reliable way to evaluate unidirectional and bidirectional causation between causally coupled variables, and is potentially an effective technique for identifying both linear and nonlinear causal coupling in brain neural networks and other settings, either random or deterministic, or both.

## Results

### Causalized CCM

In the existing CCM algorithm (Box 1), the whole time series corresponding to *X*, and both the past and future values of *Y* are all exploited to estimate the current value *Y*(*t*). That is, for two time series of length *n*, for each 1≤t≤n, *Y*(*t*) is predicted based on all $X(t_{i})$s where $1\;\leq \;t_{i}\leq \;n$, and all $Y(t_{i})$'s where $1\;\leq \;t_{i}\leq \;n$ and $t_{i}\;\neq t$.

Recall that causality aims to determine whether the current and past values of one time series are useful in predicting the future values of another time series, in addition to its own past values. In CCM, *if only the current and historical values of X and the past values of Y are used to predict the current value Y*(*t*)*, and vice versa, then* CCM *is converted to* cCCM. That is, in cCCM, we limit the search of all the nearest neighbors in $M_{x}$ to $t_{i}<t$ to predict the current value *Y*(*t*) and operate in the same way for the other direction.

**Fig. 1.**
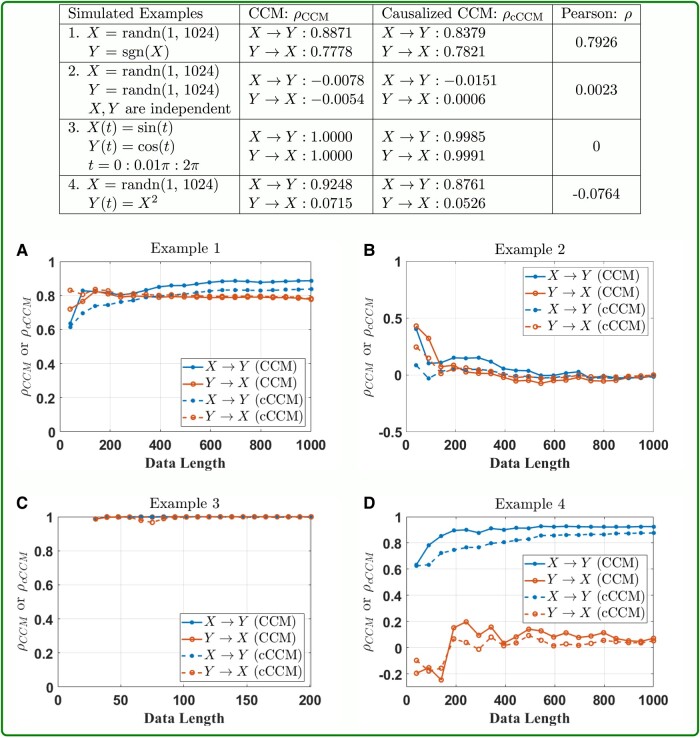
**Comparison of CCM, cCCM, and Pearson correlation based on simulation examples.** Here, randn(1, 1,024) returns a 1-by-1,024 matrix of normally distributed random numbers, *sgn* represents the sign function, t=0:0.01π:2π represents the sequence [0,0.01π,…,1.99π,2π], where the step size is 0.01π. As can be seen, CCM and cCCM are highly consistent and converge at similar speed. However, in general, correlation cannot predict causation.

The performance of CCM and cCCM is compared through simulation examples, which include both deterministic and random settings, with either bidirectional or unidirectional causation, or no causation (Fig. 1). In Example 1, *X* and *Y* are both random variables and experience bidirectional causation, but the causal effect of *X* on *Y* is stronger than that in the inverse direction. In Example 2, *X* and *Y* are independent random variables that have no causal coupling. In Example 3, *X* and *Y* are deterministic signals with strong bidirectional causation. In Example 4, *X* and *Y* are random variables, and there is a strong unidirectional causation from *X* to *Y*, but no causation in the inverse direction.

The reason that we consider normally distributed random variables in Examples 1, 2, and 4 is because the normal distribution is the most commonly occurring one in practical applications, and the reason that we consider the sinusoidal waveforms in Example 3 is because in practice, most signals can be decomposed as the superposition of sinusoidal waveforms of different frequencies through Fourier series. The simulation results for these examples are summarized in Fig. 1. Through Examples 1–4, we can see that CCM and cCCM are highly consistent and converge at similar speed, with the cCCM causation being slightly smaller than the CCM causation in general, which is expected since the latter uses a larger data set to predict the target time series. These numerical examples also show that both CCM and cCCM can be applied to different signal settings, either deterministic or random.

**Fig. 2.**
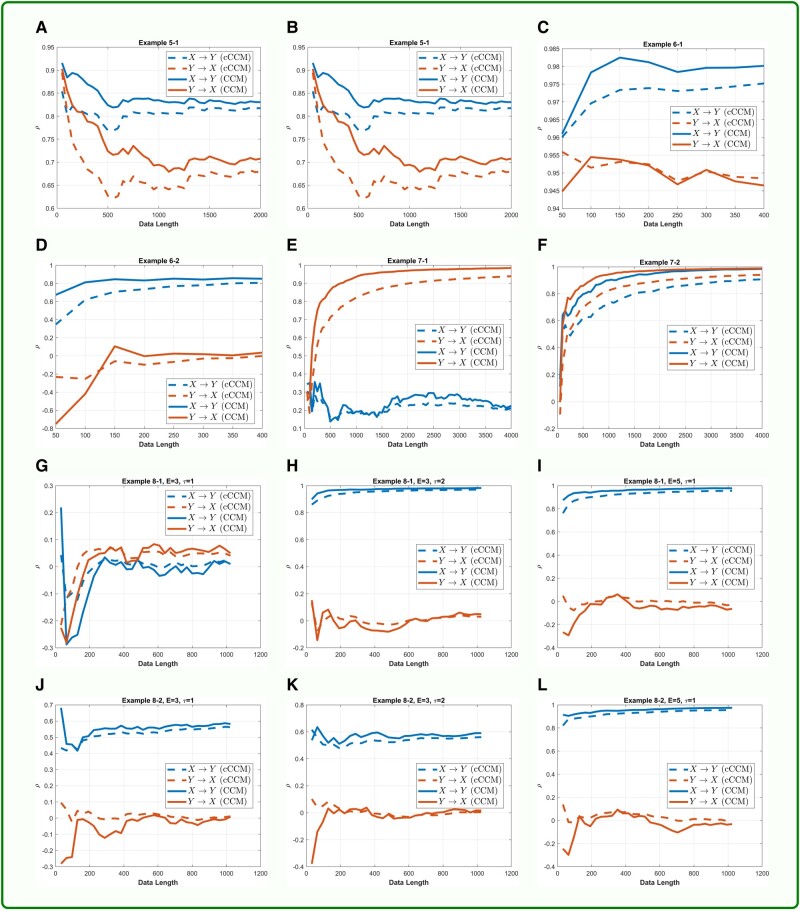
**Performance of cCCM and CCM vs. the data length:** A) Example 5-1. B) Example 5-2. C) Example 6-1. D) Example 6-2. E) Example 7-1. F) Example 7-2. G) Example 8-1, *E* = 3, *τ* = 1. H) Example 8-1, *E* = 3, *τ* = 2. I) Example 8-1, *E* = 5, *τ* = 1. J) Example 8-2, *E* = 3, *τ* = 1. K) Example 8-2, *E* = 3, *τ* = 2. L) Example 8-2, *E* = 5, *τ* = 1. In these examples, cCCM and CCM are highly consistent in causality detection and show similar convergence speed in cross-mapping. Both cCCM and CCM can detect the increase in coupling strength and show consistent sensitivity to the changes in coupling strength. Simulation results also indicate that for causality to be accurately detected in systems with memory, the product *E* · *τ* needs to be larger than the dominant delays, and for each time instant *t*, all the samples corresponding to the dominant delays need to appear in x(t)=[X(t),X(t–τ),…,X(t–(E–1)τ)],the constructing vector of the shadow manifold.

The consistence of cCCM and CCM is further demonstrated through additional examples, including autoregressive models (Examples 5-1 and 5-2), stochastic processes with a dominant spectral component embedded in noise (Examples 6-1 and 6-2), deterministic chaotic maps (Examples 7-1 and 7-2), and systems with memory (Examples 8-1 and 8-2). The results are shown in Box 2 and Fig. 2. In all these examples, cCCM and CCM are also highly consistent in causality detection and show similar convergence speed in the cross-mapping. Both cCCM and CCM can detect the increase in coupling strength and show consistent sensitivity to the changes in coupling strength.

Box 2. **cCCM vs. CCM: more simulation examples—autoregressive models, systems with dominant frequency embedded in noise, deterministic chaotic maps, and systems with memory.**

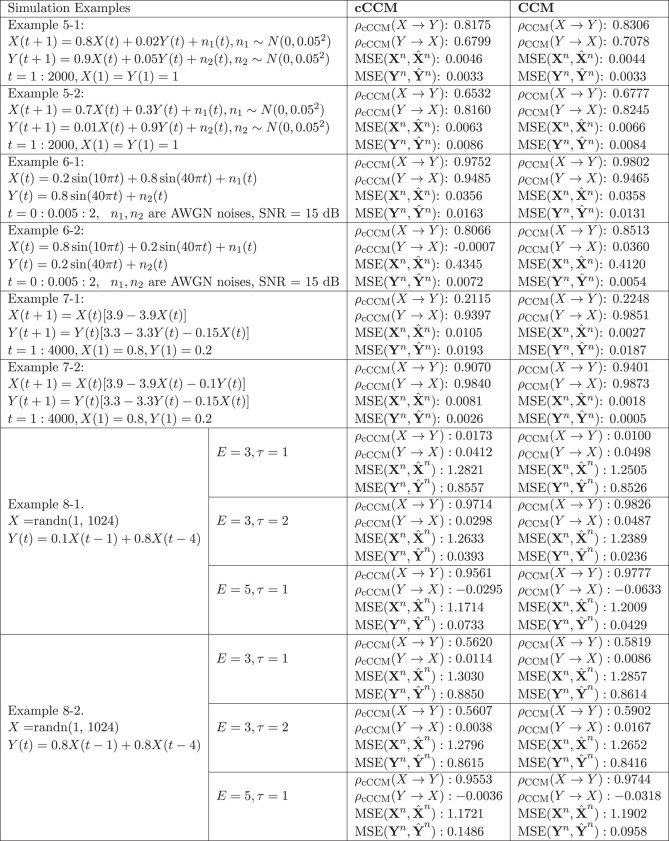

In all these examples, we generally chose E=5,τ=1. From examples on systems with memory (Examples 8-1 and 8-2), we tested different choices of *E* and *τ*, and found that the dominant delays (i.e. delays corresponding to the dominant peaks) in the channel impulse response do impose requirements on both the dimension of the shadow manifolds *E* and the signal lag *τ* used in shadow manifold construction. More specifically, for the causality to be accurately detected in systems with memory, we need: (i) The product E⋅τ is larger than the dominant delays, and (ii) For each time instant *t*, all the samples corresponding to the dominant delays appear in the constructing vector of the shadow manifold, x(t)=[X(t),X(t–τ),…,X(t–(E–1)τ)]. This was illustrated in Box 2. In Example 8-1, dominant delay = 4. If E=3,τ=1, then condition (i) is not satisfied, and the corresponding causality cannot be detected. If condition (i) is satisfied but condition (ii) is not fully satisfied, then only the causality corresponding to the dominant delays that appear in the constructing vector of the shadow manifold will be detected. This can be seen from Example 8-2, where Y(t)=0.8X(t–1)+0.8X(t–4). When E=3,τ=2, the shadow manifold constructing vector x(t)=[X(t),X(t−2),…,X(t−4)], then X(t–4) appears in x(t) but X(t–1) does not. As a result, only the causality corresponding to item 0.8X(t–4) can be detected and the causality corresponding to item 0.8X(t–1) cannot be detected. When E=5,τ=1,  x(t)=[X(t),X(t–1),…,X(t–4)], then the causality can be accurately detected since both X(t–2) and X(t–4) appear in x(t). More discussions on the choices of *E* and the signal lag *τ* used in the construction of the shadow manifolds were presented in Section 8 of Supplementary material.

### CCM causation and Pearson correlation

Examples 1–4 are also used to compare CCM causation and Pearson correlation, and explore the possible relationship between them, as shown in Fig. 1. It can be observed that when the Pearson correlation coefficient *ρ* is high, the CCM value might be high as well. This is because Pearson correlation reflects the mutual dependence between the two signals, and therefore higher Pearson correlation may imply strong causation in both directions, as shown in Example 1.

On the other hand, it can also be seen from Fig. 1 that when the Pearson correlation *ρ* is low, the cross-mapping correlation $\rho_{\mathrm{CCM}}\;\mathrm{and}$  $\rho_{\mathrm{cCCM}}\;$can either be low in both directions when *X* and *Y* are completely independent (as shown in Example 2), or high either only in one direction (as in Example 4) or in both directions (as in Example 3). From Example 3, we can see that *X* = sin(*t*) and *Y* = cos(*t*) have a 90^°^ phase shift, i.e. they are in quadrature or “orthogonal,” that is why they are not correlated. However, both CCM and cCCM can identify the strong bidirectional causation between them. In summary, Pearson correlation, very often, cannot predict the causation between two time series.

### Approximate equivalence of cCCM and DI for Gaussian variables

Recall that as a universal metric that does not rely on any model assumptions of the signals, DI serves as the pivot that links the existing causality frameworks. In this section, we first revisit the definition of DI (35), then explore the relationship between cCCM and DI using information theory and show that they are approximately equivalent under Gaussian variables.

#### Directed information

Let $X^{n}=[X_{1},X_{2},\ldots ,X_{n}]$ and $Y^{n}=[Y_{1},Y_{2},\ldots ,Y_{n}]$ denote the time series corresponding to signals *X* and *Y*, respectively. The directed information from $X^{n}$ to $Y^{n}$ is defined as


(5) $$\begin{array}{c} I(X^{n}\to Y^{n})=\overset{n}{\underset{i=1}{\sum }}\;[H(Y_{i}|Y^{i-1})-H(Y_{i}|Y^{i-1},X^{i})]=\overset{n}{\underset{i=1}{\sum }}\;I(X^{i};Y_{i}|Y^{i-1}). \end{array}$$

The average DI from *X* to *Y*, measured in bits per sample, is defined as


(6) $$\begin{array}{c} {\bar{I}}_{n}(X\to Y)=\frac{I(X^{n}\to Y^{n})}{n}. \end{array}$$

Here *H* denotes the entropy operator, H(Y|X) is the conditional entropy of *Y* given X, and I(X;Y|Z) denotes the conditional MI of *X* and *Y* given Z. The definitions of all the information measures, as well as the corresponding chain rules used in this article are summarized in Section 2 of Supplementary material.

Next, we establish the approximate equivalence of DI and cCCM by showing that: if (i) *X* and *Y* are dynamically coupled, zero-mean Gaussian random variables and their joint distribution is bivariate Gaussian, and (ii) $X^{n},\;Y^{n}\;$ are stationary ergodic Gaussian random processes, then when *n* is sufficiently large,


$${\bar{I}}_{n}(X\to Y)\approx \;-\frac{1}{2}\log\;(1-\rho_{\mathrm{cCCM},n}^{2}(X\to Y)),$$

where ${\bar{I}}_{n}$ is the average DI from *X* to *Y* per sample and $\rho_{\mathrm{cCCM},n}(X\to Y)=\rho (Y^{n},{\hat{Y}}^{n})$.

This result can be proved in the following two steps.

Step 1: If the two signals *X* and *Y* are dynamically coupled, then


(7) $$\begin{array}{c} \underset{n\to \infty }{\lim}\;{\bar{I}}_{n}(X\to Y)=\underset{n\to \infty }{\lim}\;{\bar{I}}_{n}(Y;\hat{Y}), \end{array}$$

where


$${\bar{I}}_{n}(X\to Y)=\frac{I(X^{n}\to Y^{n})}{n},\;\;\;{\bar{I}}_{n}(Y;\hat{Y})=\frac{I(Y^{n};{\hat{Y}}^{n})}{n}.$$

Step 2: If *X* and Y are zero-mean Gaussian random variables and their joint distribution is bivariate Gaussian, and $X^{n},\;Y^{n}\;$ are stationary ergodic Gaussian random processes, then when *n* is sufficiently large,


(8) $$\begin{array}{c} {\bar{I}}_{n}(X\to Y)\approx {\bar{I}}_{n}(Y;\hat{Y})\approx -\frac{1}{2}\log\;(1-\rho_{\mathrm{cCCM},n}^{2}(X\to Y)), \end{array}$$

where $\rho_{\mathrm{cCCM},n}(X\to Y)=\rho (Y^{n},{\hat{Y}}^{n})$. Steps 1 and 2 can be repeated to get the results in the reverse direction.

The first step follows from Taken's theorem (27), and the second step is based on the closed-form relationship between MI and Pearson correlation for Gaussian variables (which was established by Gel’fand (49)), as well as the Shannon–McMillan–Breiman theorem (50) which shows that when $X^{n},\;Y^{n}\;$ are stationary ergodic random processes, then $\lim_{n\to \infty }{\bar{I}}_{n}(X;Y)=I(X,Y)$. Details of the theoretical derivations are summarized in Section 4 of Supplementary material. In the rest of the paper, we follow the notation $\rho_{\mathrm{cCCM}}(X\to Y)$ from Sugihara's paper, we would like to point out that this notation is for simplicity and actually represents $\rho_{\mathrm{cCCM},n}(X^{n}\to Y^{n})=\rho (Y^{n},{\hat{Y}}^{n})$. In a strict sense, $\rho_{\mathrm{cCCM}}(X\to Y)=\lim_{n\to \infty }\rho (Y^{n},{\hat{Y}}^{n})\;.$

### fMRI-based demonstration for the approximate equivalence of cCCM and DI

The approximate equivalence of cCCM and DI is demonstrated using resting-state fMRI data, which are often modeled as Gaussian random variables (51, 52). For fMRI, we investigate the baseline data of 30 subjects from the risk reduction for Alzheimer's disease (rrAD) trial (53, 54), where 18 common ROIs of the default mode network (DMN) were extracted and sorted in a descending order by their connection strength to the isthmus of the posterior cingulate cortex seed region time course. The fMRI data of each brain region is regarded as a dynamic manifold with a deterministic attractor but perturbed by random afferent input and noise. Here, the total length of the BOLD (blood-oxygen-level-dependent) signal is 284 samples, with a sampling period of 2.5 s, i.e. the time duration of the BOLD signal is ∼12 min.

**Fig. 3.**
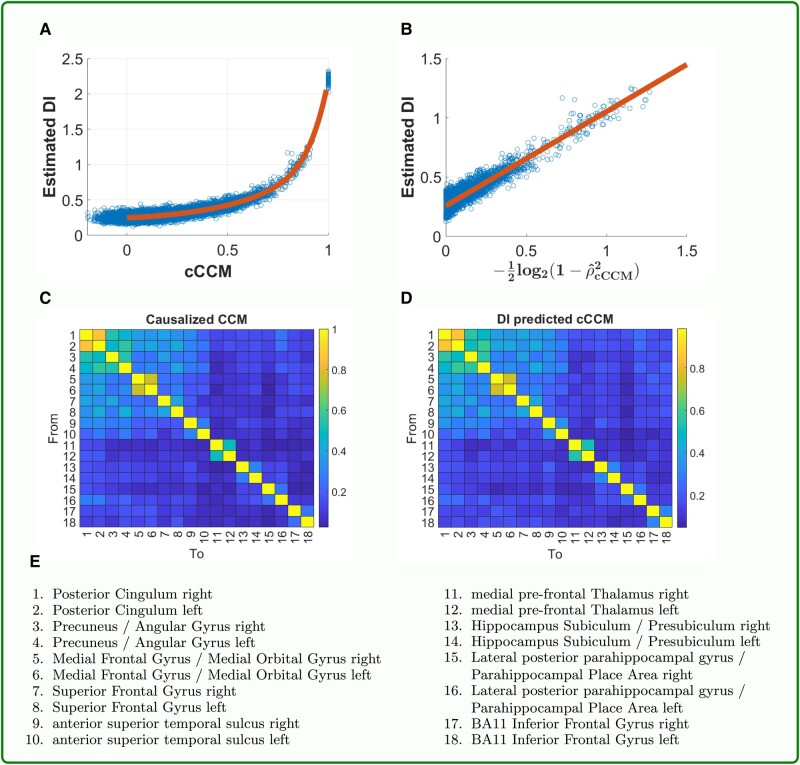
**Demonstration of the approximate equivalence of cCCM and DI using resting-state fMRI data of 30 subjects from the rrAD trials** (53, 54). A) The approximate log-relationship between estimated DI and cCCM. The relationship between cCCM and DI can be modeled as: ${\bar{I}}_{n}(X\to Y)\approx a\left[-\frac{1}{2}\;\mathrm{lo}g_{2}(1-\rho_{\mathrm{cCCM}}^{2}(X\to Y))\right]+b$ where a=0.7945,b=0.2578. The constants a,b here are largely caused by the quantization noise and error propagation in DI calculations, the finite data size, and the noise in the fMRI data. Equivalently, cCCM can be predicted from DI using: $|\rho_{\mathrm{cCCM}}(X\to Y)|=\sqrt{1-2^{-\frac{2[{\bar{I}}_{n}(X\to Y)-b]}{a}}}.$ B) The approximate linear relationship between the directly estimated DI and cCCM-predicted DI, which is given by $-\frac{1}{2}\;\mathrm{lo}g_{2}(1-\rho_{\mathrm{cCCM}}^{2}(X\to Y))$. C and D), Heatmaps for the cCCM and DI-predicted cCCM, both are averaged over all the 30 subjects. E) The region index table.

The relationship between the estimated DI and cCCM causation is illustrated in Fig. 3. Figure 3(A and B) plots the DI and cCCM values between all the 18×17=306 region pairs in the DMN for all the 30 subjects. That is, each figure has 18×17×30=9,180 points. We can see that there is a log-relationship between them—DI can be represented using cCCM and vice versa. Figure 3C and D presents the heatmaps of the cCCM and the DI-predicted cCCM values (averaged over 30 subjects), respectively. As can be seen, these two figures are highly consistent. It should be noted though, due to the finite data size, the quantization error in the digitization process of DI calculation (55), and the noise in the fMRI data, the estimated DI and $\rho_{\mathrm{cCCM}}\;$ satisfy the following relationship, which is a linear transformation of Eq. (8),


$${\bar{I}}_{n}(X\to Y)\approx a\left[-\frac{1}{2}\;\mathrm{lo}g_{2}(1-\rho_{\mathrm{cCCM}}^{2}(X\to Y))\right]+b$$

where a=0.7945,b=0.2578 in this case.

We also conducted causality analysis of the DMN using DI and cCCM for two randomly selected subjects from the rrAD trial, subject 1115 and subject 1151, respectively (see Section 6 of Supplementary material). Again, we can observe the log-relationship between DI and cCCM. Region pairs that show significantly asymmetric interactions (or say, with significant unidirectional causality), selected as pairs (i,j) where $|\rho_{\mathrm{cCCM}}(i\to j)|-|\rho_{\mathrm{cCCM}}(j\to i)|>0.15,$ were identified in Fig. S5g for subject 1115, and Fig. S6g for subject 1151. As can be seen, asymmetric interactions (or unidirectional causality) can be observed in individual subjects; however, the region pairs that show obvious unidirectional causality vary across different subjects. When the result is averaged over all the scans, as shown in Fig. 3C, the DMN network does not present dominant unidirectional causality during the resting state but shows significant bidirectional causality among regions right posterior cingulum, left Posterior Cingulum, right precuneus/angular gyrus and left precuneus/angular gyrus, where $\rho_{\mathrm{cCCM}}\;$ is bigger or close to 0.5 in both directions. This result is consistent with the previous findings in literature (5).

The cCCM causation ($\rho_{\mathrm{cCCM}}$) distribution and average node significance in DMN for all 30 subjects are shown in Fig. S7, where node significance as a transmitter and receiver is evaluated using $\sum_{j=1(j\neq i)}^{18}\left|\rho_{\mathrm{cCCM}}(i\to j)\right|$ and $\sum_{i=1(i\neq j)}^{18}\left|\rho_{\mathrm{cCCM}}(i\to j)\right|$, respectively, and $\rho_{\mathrm{cCCM}}$ for each region pair is averaged over all the 30 subjects. The pattern of bidirectional causal interactions in the averaged $\rho_{\mathrm{cCCM}}$ indicates that Posterior cingula of DMN, followed by precuneus/angular gyri, and medial frontal gyri/medial orbital gyri, act as key nodes for both information transmitting and receiving. Our result is consistent with previous findings in (5) and (56).

### Noise effect in cCCM and further comparisons on cCCM and DI

We analyzed the impact of noise by evaluating the estimation error on cCCM under noise, along the line that increased noise level may increase the prediction error in cCCM. The theoretical result is shown in Eq. (9) at the bottom of Box 3, refer Section 7 of Supplementary material for the mathematical proof of it. Our result indicates that cCCM causality tends to decrease as the noise power increases.

Box 3. **Simulation results on causality detection with and without noise (all results were averaged over 100 Monte Carlo runs).**

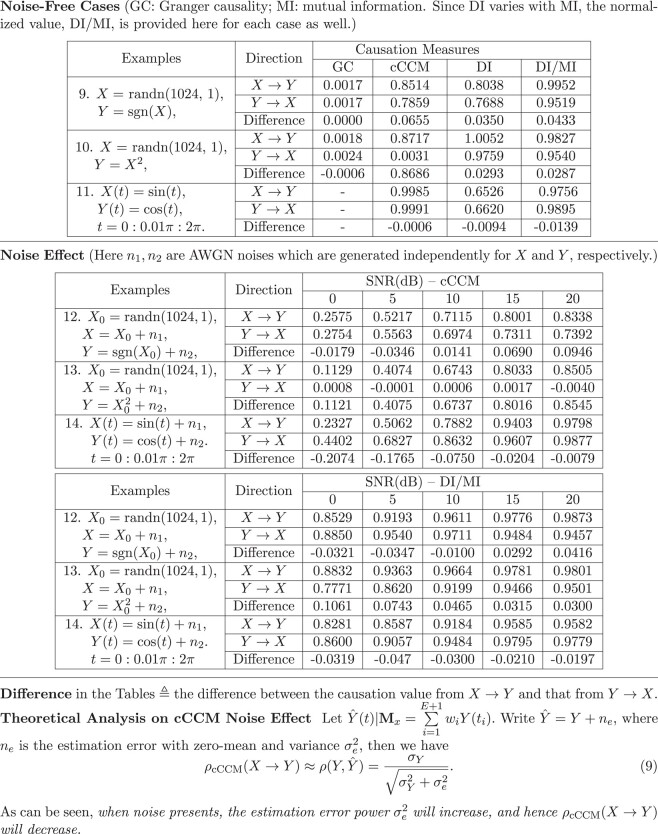

We further evaluate the noise effect in both cCCM and DI through simulation examples. As shown in Box 3, the noise-free case includes Examples 9–11, and the noisy case includes Examples 12–14. All results are averaged over 100 Monte Carlo runs (see Section 9 of Supplementary material for plots on the estimated cCCM and DI causation measures versus the number of Monte Carlo runs). It should be noted that the values of GC, cCCM, and DI cannot be compared directly, but should be explained based on their original definition. For the definition of GC, refer Section 3 of Supplementary material. As DI values vary with the corresponding MI values, both DI and its normalized version DI/MI are provided in Box 3.

#### Noise-free results

We start with the noise-free case which serves as the benchmark for noise effect analysis. From the simulation examples, we can see that the results of cCCM and DI are consistent in Examples 9 and 11, while the result of GC is quite different from them. In Example 9, both cCCM and DI show bidirectional causality but indicate that the causal effect of *X* on *Y* is slightly stronger than that in the reverse. GC is not able to identify the causal coupling between *X*(*t*) and *Y*(*t*), this is consistent with what was observed in Wang et al. (38). However, it was observed in the simulation that the GC is able to detect the causality from X(t)→Y(t−τ) and from Y(t)→X(t−τ) for 1≤τ≤20. In the Supplementary material of Sugihara et al. (17), the authors illustrated that GC causality may change back and forth with the time lag used in the data.

In Example 11, GC fails to detect the bidirectional causality between *X* and *Y* because they are deterministic functions. This is consistent with the previous findings that GC relies on the randomness in the data and may be problematic in deterministic settings (17). At the same time, while *X* = sin(*t*) and *Y* = cos(*t*) have a 90° phase shift (i.e. they are orthogonal with each other), both cCCM and DI can successfully detect the bidirectional causality between *X* and *Y*.

In Example 10, GC cannot detect any causal coupling between *X* and *Y.* cCCM can successfully identify the unidirectional causality from *X* → *Y*, and indicates that there is no significant causality from *Y* → *X.* This is what we expect since the sign information in *X* is completely lost in *Y.* On the other hand, DI not only can successfully detect the causality from *X* → *Y*, but can also indicate that there is a causal effect from *Y* → *X*, which is unlikely to be true. This may be due to the offsets in probability estimation and possible error propagation in DI calculation (55) and needs further exploration. Here, *X* is Gaussian, but *Y* is not, so they no longer satisfy the conditions for the approximate equivalence between cCCM and DI, which also explains why DI and cCCM have different performances in this example.

From these examples, we can see that it may be difficult for GC to detect causality in deterministic and nonlinear settings (without time lags), and cCCM and DI may detect both deterministic and statistical causality in nonlinear settings. Moreover, it was also observed that cCCM tends to be more robust or sensitive than DI for causality detection, especially when *X* and *Y* have significantly different ranges (which is a challenging task for the DI estimation algorithm (55)), with the price of a much higher computational complexity. Relying on exhaustive search of nearest neighbors, the computational complexity of cCCM is $O(n^{2})$ for two sequences of length n, which is much higher than that of DI, where the computational complexity is linear in the sequence length *n*, O(n).

#### Impact of the noise

To illustrate the effect of noise on cCCM and DI, we evaluate the causality of the simulation data sets in Examples 9–11 under additive white Gaussian noise for different SNR levels, as shown in Examples 12–14, respectively. As the SNR increases from 0 to 20 dB, the estimated cCCM and DI causation gradually converge to that in the noise-free case. The simulation result on cCCM echoes our theoretical analysis, where it was proved that the estimated causality would decrease as the noise power increases. However, when the SNR is sufficiently large (i.e. SNR ≥ 15 dB), cCCM can deliver reliable results.

### Extension of bivariate cCCM to multivariate conditional cCCM

This method aligns with the multivariate KNN predictability approaches, which are also based on the embedding theorem and geometric cross-mapping for state-space reconstruction (28–31), and provides one way to extend bivariate cCCM to multivariate conditional cCCM. The same approach can be applied to CCM as well.

Let $\Omega =\{X_{1},\ldots ,X_{Q}\}$ be the set of dynamically coupled random variables which share the same attractor manifold M. For q=1,…,Q, let $X_{q}^{n}=[X_{q,1},X_{q,2},\ldots ,X_{q,n}]$ denote the time series consisting of samples of $X_{q}$ and construct the shadow manifold with respect to $X_{q}^{n}$ as


(10) $$\begin{array}{c} M_{X_{q}}=\{x_{q,t}|x_{q,t}=[X_{q,t},X_{q,t-\tau },\ldots ,X_{q,t-(E-1)\tau }],t=1+(E-1)\tau ,\ldots ,n\},\;q=1,\ldots ,Q. \end{array}$$

For q=1,…,Q, let ${\hat{X}}_{i,t}|M_{X_{q}}$ be the estimated $X_{i,t}$ based on $M_{X_{q}}.$ Let ${\hat{X}}_{i,t}|\Omega$ denote the multivariate prediction of $X_{i,t}$ based on all the ${\hat{X}}_{i,t}|M_{X_{q}}$, q∈{1,…,Q},q≠i. That is,


(11) $$\begin{array}{c} \begin{array}{c} \;{\hat{X}}_{i,t}|\Omega =\overset{Q}{\underset{q=1,q\neq i}{\sum }}\;a_{q}{\hat{X}}_{i,t}|M_{X_{q}}+e_{i,t}|\Omega ,\; \end{array} \end{array}$$

where the coefficients $a_{q}$, q∈{1,…,Q}, and q≠i, are selected to minimize the variance of the estimation error $e_{i,t}|\Omega .$ Similarly, ${\hat{X}}_{i,t}|\Omega \setminus \{X_{j}\}$ denotes the multivariate prediction of $X_{i,t}$ based on all the ${\hat{X}}_{i,t}|M_{X_{q}}$, q∈{1,…,Q} but q≠i,j, that is


(12) $$\begin{array}{c} \begin{array}{c} \;{\hat{X}}_{i,t}|\Omega \setminus \{X_{j}\}=\overset{Q}{\underset{q=1,q\neq i,j}{\sum }}\;b_{q}{\hat{X}}_{i,t}|M_{X_{q}}+e_{i,t}|\Omega \setminus \{X_{j}\}, \end{array} \end{array}$$

where the coefficients $b_{q},q\in \{1,\ldots ,Q\}$ but q≠i,j, are selected to minimize the variance of the estimation error $e_{i,t}|\Omega \setminus \{X_{j}\}.$ Following Eqs. (11) and (12), define the estimation error vectors as:


$$\begin{array}{rl} & e_{i}^{n}|\Omega =[e_{i,1}|\Omega ,\ldots ,e_{i,n}|\Omega ],\\ & e_{i}^{n}|\Omega \setminus \{X_{j}\}=[e_{i,1}|\Omega \setminus \{X_{j}\},\ldots ,e_{i,n}|\Omega \setminus \{X_{j}\}].\begin{array}{cc} \; & \;\\ \; & \; \end{array} \end{array}$$

Following (29), the CR from $X_{j}\to X_{i}$ is defined as


(13) $$\begin{array}{c} \begin{array}{c} CR_{X_{j}\to X_{i}}=\frac{\mathrm{Var}(e_{i}^{n}|\Omega \setminus \{X_{j}\})-\mathrm{Var}(e_{i}^{n}|\Omega )}{\mathrm{Var}(e_{i}^{n}|\Omega \setminus \{X_{j}\})} \end{array} \end{array}$$

A natural question is: *does the approximate equivalence still hold for multivariate conditional cCCM and DI?* In general, the relationship between multivariate KNN search-based conditional cCCM and conditional DI becomes very complicated and uncertain, and approximate equivalence may no longer holds, as in the case between conditional DI and DCM (48). This part still needs to be further explored.

### Detection of unidirectional causality in brain network based on visual task-driven fMRI

Here, we consider another fMRI dataset where 14 right-handed healthy college students (7 males and 7 females, 23.4 ± 4.2 years of age) from Michigan State University volunteered to participate in a task-driven fMRI-based study (38, 57, 58). For each subject, fMRI datasets were collected on a visual stimulation condition with a scene-object fMRI paradigm, where each volume of images was acquired 192 times (8 min) while a subject was presented with 12 blocks of visual stimulation after an initial 10 s resting period. In a predefined randomized order, the scenery pictures were presented in six blocks and the object pictures were presented in other six blocks. All pictures were unique. In each block, 10 pictures were presented continuously for 25 s (2.5 s for each picture), followed by a 15-s baseline condition (a white screen with a black fixation cross at the center). The subject needed to press his/her right index finger once when the screen was switched from the baseline to picture condition. More details on data acquisition and preprocessing are given in Materials and methods section.

#### ROI selection

We selected 10 ROI regions, including: left primary visual cortex (LV1), left parahippocampal place area (LPPA), left sensory motor cortex (LSMC), left parahippocampal white matter (LPWM), left retrosplenial cortex (LRSC), right primary visual cortex (RV1), right parahippocampal place area (RPPA), right sensory motor cortex (RSMC), right frontal white matter (RFWM), and right retrosplenial cortex (RRSC).

#### Result for bivariate cCCM

Recall that the total length of the fMRI BOLD time series under visual stimulation condition is n=192, with the sampling period being 2.5 s. We conducted causality analysis for all the possible unidirectional regional pairs using CCM, cCCM, and DI. However, since CCM and cCCM did not converge with the data length n=192, we chose to interpolate the fMRI sequence by a factor of 2 using the spline interpolation command in MATLAB, which reduced the sampling period from 2.5 to 1.25 s and increased the data length to (2×192)−1=383. We then applied CCM, cCCM, and DI to the interpolated sequences and obtained *consistent* results on unidirectional causality from all the three models.

**Fig. 4.**
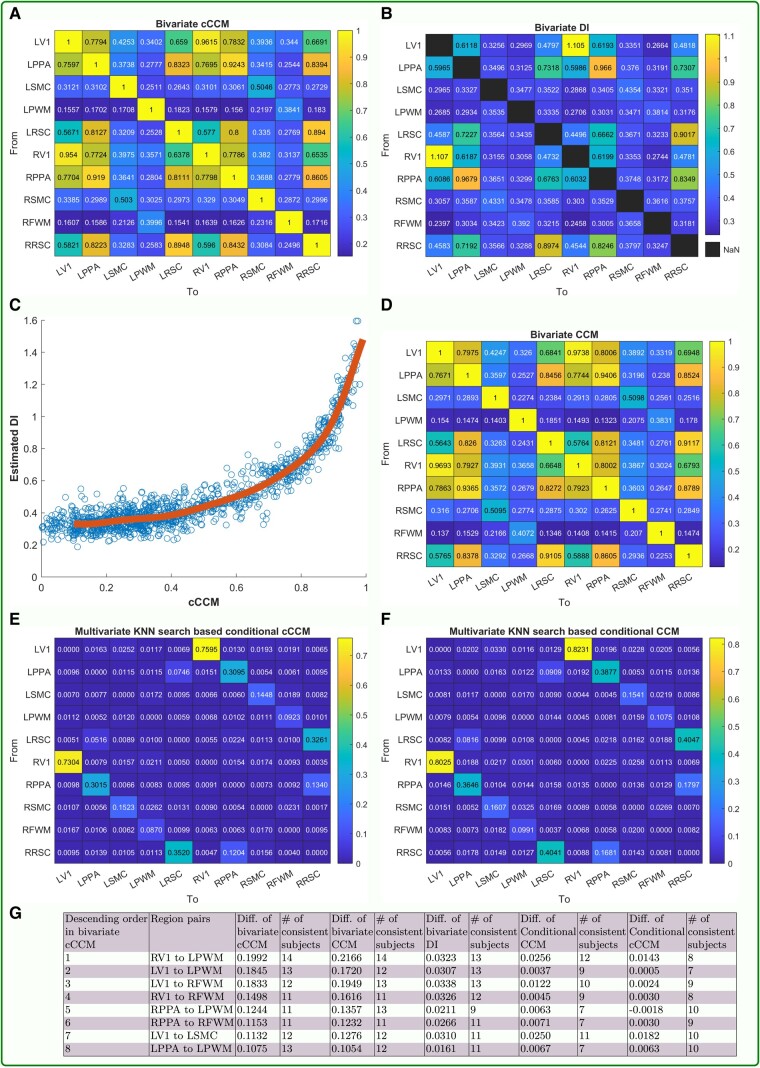
**Brain causality analysis using CCM, cCCM, and DI based on visual task-fMRI.** A) Heatmap of bivariate cCCM. B) Heatmap of bivariate DI. C) Approximate equivalence between cCCM and DI. D) Heatmap of bivariate CCM. E and F) Heatmap of multivariate conditional cCCM and CCM with respect to all the other ROI regions, respectively. G) ROI region pairs that show consistent unidirectional causality across bivariate cCCM, CCM, DI, and the majority of subjects. Here, *Diff*. means the causation difference in two opposite directions.

The consistence of CCM, cCCM, and DI in the detection of unidirectional causality (averaged over all the 14 subjects) is shown in Fig. 4(A–D and G), where the approximate equivalence of cCCM and DI is shown in Fig. 4C. The ROI region pairs which show consistent unidirectional causality across bivariate CCM, cCCM, DI, and across majority of the subjects for all these three models are shown in Fig. 4G. Here region pairs with averaged causation difference (between the two opposite directions) larger than 0.1 for both bivariate CCM and cCCM, and with average difference larger than 0.01 for DI were identified as ROI pairs with significant unidirectional causality, which include: RV1 → LPWM, LV1 → LPWM, LV1 → RFWM, RV1 → RFWM, RPPA → LPWM, RPPA → RFWM, LV1 → LSMC, and LPPA → LPWM. As can be seen, CCM and cCCM resulted in much larger causation difference in the two opposite directions than that of DI, and hence is more *robust* than DI in identifying unidirectional causality in brain networks for this task-driven fMRI dataset.

It is worth noting that CCM, cCCM, and DI all identified the same unidirectional causal relations among these ROI pairs, with both the original BOLD sequences and the interpolated sequences. This implies that even if CCM and cCCM did not converge with *n* = 192 samples, they were still able to identify the unidirectional causal relations correctly, but interpolation of the fMRI sequence did enhance the robustness of CCM- and cCCM-based causality analysis. In literature, it was also reported by Lin et al. in 2014 (59) that increasing the sampling rate of the fMRI signal can improve the robustness of causality analysis.

*The interpretation of the unidirectional causality from V1 and PPA to LPWM and RFWM needs to be carefully made* since the white matter regions do not contain the cell bodies of the neurons, which are located in the gray matter. As a result, the recorded BOLD signals in the white matter tend to be noisy and have relatively low power levels compared with other ROIs. In fact, the average power levels of the BOLD signals of LPWM and RFWM are 5.28 and 6.71, respectively, which is much lower than that of the other ROI regions. More specifically, LV1 and RV1 turn out to have the highest average power levels among all the ROIs, which are 37.79 and 37.97, respectively; the average power levels of LSMC and RSMC are 11.88 and 12.17, respectively, which are the lowest among all the ROIs except the white matter.

On the other hand, while gray matter is a common focus in fMRI studies, recent studies have consistently found that BOLD signals can be reliably detected in white matter, and brain functional connectivity has been organized into distributed networks in white matter (60, 61). Our result on unidirectional causality echoes the existing findings and indicates that neuronal activity in V1 and PPA might lead to the information transfer activity in the white matter, which connects regions that send and receive signals. This is similar to the situation in human society—when we have some thoughts, we may start to communicate with our friends using a phone or computer, either through the internet or the airlink. Although the phone, computer, internet, or airlink themselves do not own or generate the thoughts, information transmitting and receiving activities can be observed from them, and the unidirectional causality here lies in that the information exchange activity is caused by the thinking activity in human beings.

If we exclude white matter from the result and focus on the cortical regions, then only one ROI region pair, LV1→ LSMC, demonstrated significant unidirectional causality. In addition, RV1 → LSMC also showed apparent unidirectional causality (though not included in Fig. 4), where the difference between $\rho_{\mathrm{CCM}\;}$ in the two opposite directions (i.e. RV1 → LSMC and LSMC → RV1) is 0.1018 with 11 consistent subjects, the difference between $\rho_{\mathrm{cCCM}\;}$ in the two opposite directions is 0.0875 with 12 consistent subjects, and the corresponding difference for DI being 0.0343 with 12 consistent subjects. The unidirectional causality from LV1 → LSMC and RV1 → LSMC in visual task-fMRI aligns with the experimental design where the subjects saw the picture before pressing the finger.

In addition, from Fig. 4, it can also be observed that significant bidirectional causality exists between many ROI region pairs, such as LV1 and RV1, LPPA and RPPA, LRSC and RRSC, etc.

#### Result for multivariate conditional cCCM

We also conducted multivariate conditional CCM and conditional cCCM for all the 10×9=90 unidirectional ROI pairs using the same interpolated fMRI sequences of length *n* = 383 as in the bivariate case.

The cCCM and CCM causality ratios for each ROI pair *conditioning on all the rest of the state space* are shown in Fig. 4(E and F). As can be seen, both conditional CCM and cCCM are very sensitive to the interdependence between the brain regions under consideration and the rest of the regions in the state space. Due to rich brain network diversity, the multivariate conditional CCM and cCCM causality ratios with respect to the rest of the state space turn out to be very small or insignificant and cannot really be used for unidirectional causality detection. That is, when we consider the conditional causality from $X_{j}$ → $X_{i}$, if $X_{j}$ is largely dependent on the random variables in *Ω* ∖ $\;\{X_{i},X_{j}\}$, then the KNN-based conditional CR would be very small even if there exists (bivariate) unidirectional causality from $X_{j}\to X_{i}$.

**Fig. 5.**
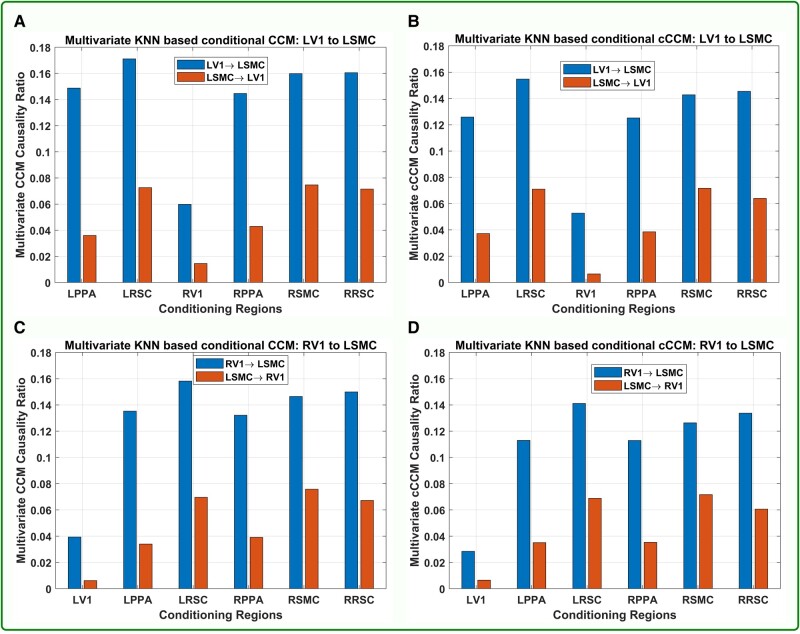
**Multivariate conditional CCM and cCCM with respect to individual regions**. A) Conditional CCM from LV1 → LSMC. B) Conditional cCCM from LV1 → LSMC. C) Conditional CCM from RV1 → LSMC. D) Conditional cCCM from RV1 → LSMC. The results indicate that: (i) Conditional CCM and cCCM causality ratios with respect to individual regions are highly consistent; (ii) RV1 has the most significant impact on the conditional causality from LV1 → LSMC, and LV1 has the most significant impact on the conditional causality from RV1 → LSMC. This implies that RV1 has the highest interdependence with LV1, followed by LPPA and RPPA.

We further checked the multivariate conditional CCM and cCCM causality *with respect to individual regions*. More specifically, we compared the conditional CCM and cCCM causality from LV1 → LSMC, RV1 → LSMC with respect to all the other individual ROI regions and the result is shown in Fig. 5. It can be observed that: (i) Conditional CCM and cCCM ratios with respect to individual regions are highly consistent; (ii) RV1 has the most significant impact on the conditional causality from LV1 → LSMC, and LV1 has the most significant impact on the conditional causality from RV1 → LSMC. This implies that RV1 has the highest interdependence with LV1, followed by LPPA and RPPA, and the result is consistent with the strengths of causal coupling among the ROI regions, as shown in Fig. 4(A, B, and D). In short, our numerical analysis indicates that conditional cCCM and CCM with respect to individual regions can detect unidirectional causality and demonstrate the impact of interdependence between the ROI regions on the conditional causality.

## Discussion

In this article, we first causalized CCM so that it aligns with the traditional definition of causality—in the sense that the future values of one process cannot influence the past of the other—and demonstrated the consistence of cCCM and CCM through representative simulation examples. We then showed the approximate equivalence of cCCM and DI under Gaussian variables and established an approximate mathematical relationship between them. This result links cCCM to other representative causality analysis frameworks in the family—GC, TE, and DCM, in the sense that they are all approximately equivalent to each other under certain conditions.

We explored the possible relationships between correlation and causation, and showed that high correlation might imply high causation, but low correlation may be connected to either high or low causation, either unidirectional or bidirectional. This is consistent with the findings in previous work that, in general, correlation cannot predict causation (17). We also tested the noise effect on cCCM and showed that in the presence of noise, the cCCM causation decreases, but will get close to that in the noise-free case when the SNR is 15 dB or higher.

We compared the performance of DI and cCCM when the signals are not jointly Gaussian and hence DI and cCCM are no longer approximately equivalent. In the simulation examples, it was observed that cCCM tends to be more robust in causality detection, especially when the two sequences under consideration have significantly different ranges, which is a condition that raises significant challenges for DI calculation. This is because DI relies heavily on probability estimation, which is sensitive to the digitization or quantization process used in the implementation algorithm (55). cCCM, on the other hand, gets around the problem of probability estimation through geometric cross-mapping of the corresponding neighborhoods between the manifolds involved, at the cost that exhaustive KNN search (here K=E+1and *E* is the dimension of the shadow manifold) is required at the prediction of every sample of the target time series. The disadvantage of cCCM, therefore, is its high computational complexity, which is $O(n^{2})$ in the sequence length n, while the computational complexity of DI is only O(n).

In the implementation of the cross-mapping technique, the choice of the dimension of the shadow manifold plays a critical role. According to Takens’ theorem (27) and Whitney's embedding theorem (62), the magic number is E=2d+1, and often less (17), where *d* is the dimension of the attractor shared by the two time series under consideration. If *E* is too large, cCCM may no longer deliver meaningful results (see Section 8 of Supplementary material for simulation results).

Taking the interdependence between different random variables in a multivariate system into consideration, we extended bivariate CCM and cCCM to multivariate conditional CCM and cCCM along the lines of multivariate KNN predictability approaches (29, 30). Whether the approximate equivalence between multivariate conditional cCCM and DI still holds is an open question that needs further investigation. An effective way to estimate multivariate conditional DI also needs to be explored.

Our numerical analysis based on experimental fMRI indicated that in the resting state, DMN shows significant bidirectional causality among certain regions (mainly right posterior cingulum, left Posterior Cingulum, right precuneus/angular gyrus, and left precuneus/angular gyrus), but no dominant unidirectional causality was observed. On the other hand, analysis based on a visual task-driven fMRI dataset showed that unidirectional causality among the ROI regions (mainly from the left and right primary visual cortices to LSMC and to the related white matter regions) can be detected by cCCM during the performance of tasks, and the result is consistent with that of CCM and DI and is also consistent across the majority of subjects. In addition, due to rich diversity in the brain network, multivariate conditional CCM and cCCM conditioning on all the rest of ROI regions generally result in very small causality ratios and cannot be used for causality detection. However, conditional cCCM and CCM with respect to individual regions can detect unidirectional causality and demonstrate the impact of interdependence between the ROI regions on the conditional causality.

For future work, we will continue to explore the (conditional) causal relationships in brain networks under both resting-state and task-driven scenarios, with the expectation that the study on causality may help identify the possible paths on directed information flow among brain regions and provide us a better understanding on the region-level information transmission mechanisms in the brain. In addition, to study the transitive causal chains (63) in the brain network, we will investigate time-delayed causal interactions among brain regions to possibly determine the order of variables in each transitive information processing and transmission chain.

We also explored the impact of sampling frequency on cCCM through simulation examples, refer to Section 10 of Supplementary material. Again, sinusoid waveforms were used here since they can serve as the building blocks to approximate most functions encountered in reality. As can be seen, cCCM works well when the sampling rate is well above the Nyquist rate and may require larger data length to converge as the sampling frequency is above but very close to the Nyquist sampling rate; when the sampling frequency is below or equal to the Nyquist rate, very often, cCCM can no longer deliver meaningful results.

In the case when the two signals under consideration are periodic, CCM and cCCM are equivalent since the future is identical with the past for periodic signals. When the data length of the time series under consideration, say X and *Y*, is very limited, can we concatenate the time series to increase the data length and consider the causality of [X,X] and [Y,Y] instead, for example? The answer is no. This is because concatenation can create new causality which does not exist in the original X and *Y*. In fact, even if X and *Y* are two independent time series that are not causally coupled, a causal pattern is enforced in the concatenated time series through signal repetition (see Section 11 of Supplementary material for simulation result).

Overall, our analysis shows that the cross-mapping technique is easy to implement and is a promising tool for identifying both linear and nonlinear causal coupling in different settings, either random or deterministic, or both. It has attracted considerable attention in the research communities, and we believe that a broad spectrum of applications is yet to come.

## Materials and methods

In this study, we utilized two experimental fMRI datasets.

### Dataset 1: resting-state fMRI

#### Data description

Here, we investigate the baseline data of 30 subjects (age 60–83, 17 men, and 13 women) from the rrAD trial (53, 54). All study procedures were approved by the institutional review board at University of Texas Southwestern Medical Center, University of Kansas Medical Center Research Institute, Pennington Biomedical Research Center, and Washington University in St Louis, and each participant provided written, informed consent. A detailed description of the imaging protocol of the rrAD trial, including inclusion and exclusion criteria, has been provided by Szabo-Reed et al. (64). Overall, individuals with a history of severe neurological, psychological, cardiovascular, and other severe diseases were excluded from this study. Included subjects underwent baseline resting-state fMRI acquisition (eyes focused on a cross) for 12 min on a GE MR750W 3T MRI system with a 48-channel head/neck coil, located at the University of Texas Southwestern Medical Center. The fMRI data were acquired with 2.5 s TR (time of repetition), 28 ms TE (time of echo), and a 64 × 64 matrix size with 3.4 mm × 3.4 mm pixels. Slices with a thickness of 3.4 mm were recorded without parallel imaging.

In addition to the functional images, anatomical 3D 1-mm^3^ isotropic T1-weighted Magnetization-Prepared Rapid Acquisition Gradient Echo (MPRAGE) images with cerebrospinal fluid (CSF) suppressed were also collected for each of the subjects using the following parameters: 176 sagittal slices, TE = 3.8–4 ms, TR of acquisition ≍8.6 ms, time of inversion (TI) = 830 ms, TR of inversion = 2330 ms, flip angle = 8°, FOV (field of view) = 25.6 cm × 25.6 cm, matrix size = 256 × 256, slice thickness = 1 mm, and parallel imaging acceleration factor = 2.

#### fMRI preprocessing and noise regression

The data of each subject was first processed with the AFNI (65) proc.py script (AFNI's tool to create a complete and standardized fMRI preprocessing pipeline). This script includes the steps of outlier detection, despiking, correction for slice-timing differences, functional image coregistration to anatomical recordings, alignment between functional volumes to correct for rigid motions, and smoothing using a 4-mm kernel. We then transformed the motion parameters extracted by AFNI's motion correction into an FSL compatible format and performed aggressive ICA-AROMA (66) before applying a bandpass filter (0.009–0.08 Hz) and third-order detrending.

Using DARTEL (67) from SPM (68) and the anatomical MPRAGE images, we created a common template and normalized all subject's fMRI data into the common space. Using the isthmus of the posterior cingulate cortex as the seed region for each subject, we created seed-based connectivity maps of the DMN for each subject. From these connectivity maps, 18 common ROI of the DMN were extracted and sorted in a descending order by their connection strength to the isthmus of the posterior cingulate cortex seed region time course.

### Dataset 2: visual task-driven fMRI

#### Data acquisition

Fourteen right-handed healthy college students (seven males, 23.4 ± 4.2 years of age) from Michigan State University volunteered to participate in this study. All subjects provided informed consent. All experimental procedures were approved by the Michigan State University Institutional Review Board (38, 57, 58). The experiment was conducted on a 3-T GE Signa HDx MR scanner (GE Healthcare, Waukesha, WI, USA) with an eight-channel head coil. For each subject, fMRI datasets were collected on a visual stimulation condition with a scene-object fMRI paradigm, and then, on a resting-state condition. The parameters for the fMRI scan were: gradient-echo echo planar imaging, 36 contiguous 3-mm axial slices in an interleaved order, time of echo = 27.7 ms, time of repetition = 2,500 ms, flip angle = $80^{\circ }$, FOV = 22 cm, matrix size = 64×64, ramp sampling, and with the first four data points discarded. On the visual stimulation fMRI condition, each volume of images was acquired 192 times (8 min) while a subject was presented with 12 blocks of visual stimulation after an initial 10 s resting period. In a predefined randomized order, the scenery pictures were presented in six blocks and the object pictures were presented in other six blocks. All pictures were unique. In each block, 10 pictures were presented continuously for 25 s (2.5 s for each picture), followed by a 15-s baseline condition (a white screen with a black fixation cross at the center) (38, 57, 58). The subject needed to press his/her right index finger once when the screen was switched from the baseline to picture condition. Stimuli were displayed in color on full screen on a 1,024×768 32-in LCD monitor (Salvagione Design, Sausalito, CA, USA) placed at the back of the magnet room. The LCD subtended $10.2^{o}\times 13.1^{o}$ of visual angle. On the rs-fMRI condition, each volume of images was acquired 164 times (6 min and 50 s) after a subject was informed to relax, keep his/her eyes closed, and stay awake throughout the scan. After the aforementioned functional data acquisition, high-resolution volumetric T_1_-weighted spoiled gradient-recalled images with CSF suppression were obtained to cover the whole brain with 120 1.5-mm sagittal slices, $8^{o}$ flip angle, and 24 cm FOV. These images were used to identify anatomical locations (38, 57, 58).

#### Data preprocessing

All stimulus fMRI data preprocessing and analysis for each subject were conducted with AFNI software (65) as described in Henderson et al. (57). Essentially, slice-timing correction and rigid-body motion correction were carried. Spatial blurring with a full width half maximum of 4 mm was applied to reduce random noise. Multiple linear regressions (using the “3dDeconvolve” routine in AFNI) were applied on a voxel-wise basis to find the magnitude change when each picture condition was presented, followed by general linear tests to find the statistical significances between stimulus conditions. The ROI in this study was defined in the Talairach coordinate space (69). Regions showing preferential activation to scenes over objects (voxel-based *P*-value < $10^{-4}$) in the right and left parahippocampal gyri were defined as the RPPA and LPPA (57). The right and left V1 ROIs were defined as the regions activated by pictures (voxel-based *P*-value < $10^{-10}$) within Brodmann area 17. Because there was a high level of activation at and around V1, a highly conservative *P*-value threshold was chosen to define relatively focal ROIs. The RSMC and LSMC spherical ROIs with a 6-mm radius were defined with the centers at (R36, P22, S54) and (L38, P26, S50) correspondingly in the Talairach coordinate space (R = Right, L = Left, P = Posterior, S = Superior). The SMC coordinate locations were defined by Witt et al. (70) and the ROIs were created as in Zhu et al. (58). The time courses from the stimulation fMRI dataset that were already preprocessed as previously were detrended and had their baselines removed also. The spatially averaged time course at each of the aforementioned ROIs was generated for the causality analyses.

## Supplementary Material

pgad422_Supplementary_Data

## Data Availability

The fMRI datasets presented in this study are available to qualified investigators according to the NIH data-sharing policy upon request. The rrAD public dataset can be accessed at https://rradtrial.org/. The 1D files corresponding to the task-fMRI can be accessed at https://github.com/dengjinx/CCM-cCCM-DI.git. Those who are interested in the original task-fMRI datasets should contact D.C.Z. (zhuda@msu.edu) to arrange a research agreement for data sharing. All the other data supporting the findings of this study are available within the article and its Supplementary material. The relevant MATLAB code can also be found at https://github.com/dengjinx/CCM-cCCM-DI.git.
