# Supplementary material for: Causalized convergent cross-mapping and its approximate equivalence with directed information in causality analysis
Source: PNAS Nexus. 2023 Dec 7;3(1):pgad422. doi: 10.1093/pnasnexus/pgad422 (PMC10758925; doi:10.1093/pnasnexus/pgad422)
Supplement: pgad422_Supplementary_Data [file pgad422_supplementary_data.pdf]

Causalized Convergent Cross Mapping and its Approximate Equivalence with Directed Information in Causality Analysis—Supplementary File

Jinxian Deng, Boxin Sun, Norman Scheel, Alina B. Renli,
David C. Zhu, Dajiang Zhu, Jian Ren, Tongtong Li* and Rong Zhang*

Contents

1 Illustration of the Cross-Mapping Concept **2**
- 1.1 Schematic Diagram for Cross-Mapping 2
- 1.2 Illustration of the Cross-Mapping Concept using Shadow Manifolds Generated from Resting-State fMRI Data 3

2 Information Measures and Chain Rules **6**
- 2.1 Information Measures 6
- 2.2 Chain Rules 6

3 Some Representative Causality Analysis Frameworks: GC, DI, TE and DCM **7**

4 The Approximate Equivalence between cCCM and DI for Gaussian Variables **9**
- 4.1 Closed-Form Relationship between Pearson Correlation and Mutual Information 9
- 4.2 Approximate Equivalence between Causalized-CCM and DI 10

5 Multivariate Conditional cCCM **13**
- 5.1 A Simulation Example on Conditional Causality 13
- 5.2 Multivariate KNN search based conditional cCCM 13

6 Additional Results on Brain Causality Analysis based on Experimental fMRI Data **14**
- 6.1 Causality Analysis of the Default Mode Network based on Resting-State fMRI Data 14
- 6.2 Unidirectional Causality Analysis of the Brain Network based on Task-Driven fMRI Data . 18

7 The Impact of Estimation Error on cCCM **23**

8 Discussions on the Choice of the Shadow Manifold Dimension E and Signal Lag τ **24**

9 How Many Monte Carlo Runs Do We Need to Evaluate the Noise Effect in cCCM and DI ? **30**

10 An Example on the Impact of Sampling Frequency on cCCM **33**

11 An Example on Data Repetition in Causality Analysis **34**

1 Illustration of the Cross-Mapping Concept

Based on Takens' theorem, if two variables X and Y are causally linked, then the nearest neighbors of $\mathbf{x}_t$ in $\mathbf{M}_x$ will be mapped to the nearby points of $\mathbf{y}_t$ in manifold $\mathbf{M}_y$. This would no longer hold when X and Y are not causally linked. In the following, we illustrate the CCM concept through both schematic diagrams, as well as the shadow manifolds generated using experimental fMRI data.

1.1 Schematic Diagram for Cross-Mapping

The cross-mapping from $\mathbf{M}_x$ to $\mathbf{M}_y$ is shown below under two different scenarios. Supplementary Figure 1 illustrates the case when there is a strong causation from X to Y, Supplementary Figure 2 illustrates the opposite situation. Here, the dimension of the shadow manifold is $E = 2$, and the simplex that represents the neighborhood of $\mathbf{x}_t$ consists of three nearest neighbors, similarly for the neighborhood of $\mathbf{y}_t$.

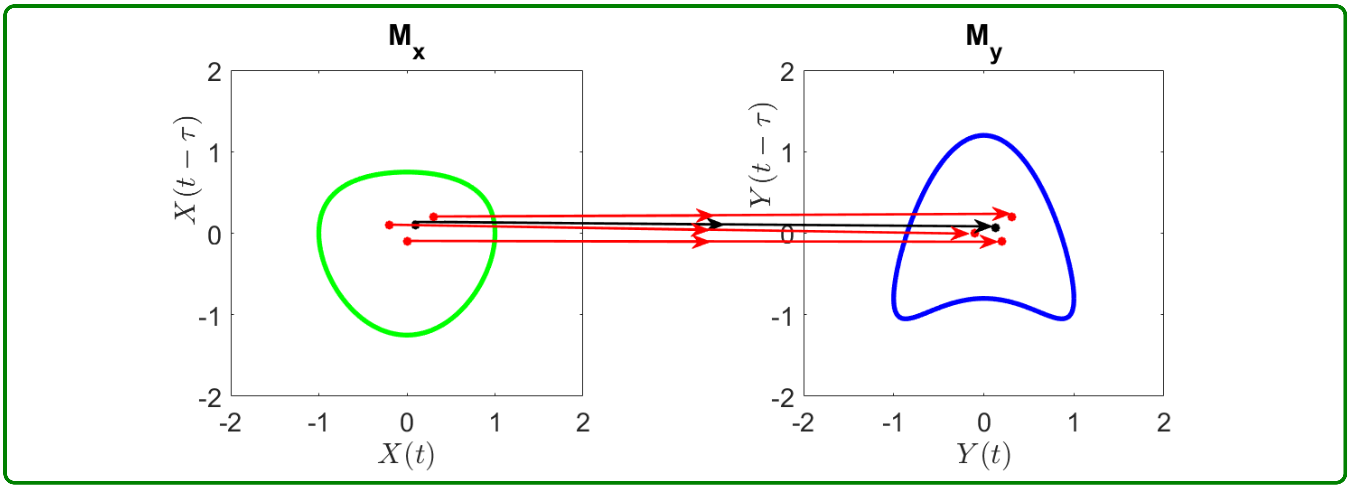

Supplementary Figure 1: **Cross-mapping from $\mathbf{M}_x$ to $\mathbf{M}_y$** when there is a strong causation from X to Y. In this case, the nearest neighbors of $\mathbf{x}_t$ (the black dot in $\mathbf{M}_x$) are mapped to the nearest neighbors of $\mathbf{y}_t$ (the black dot in $\mathbf{M}_y$).

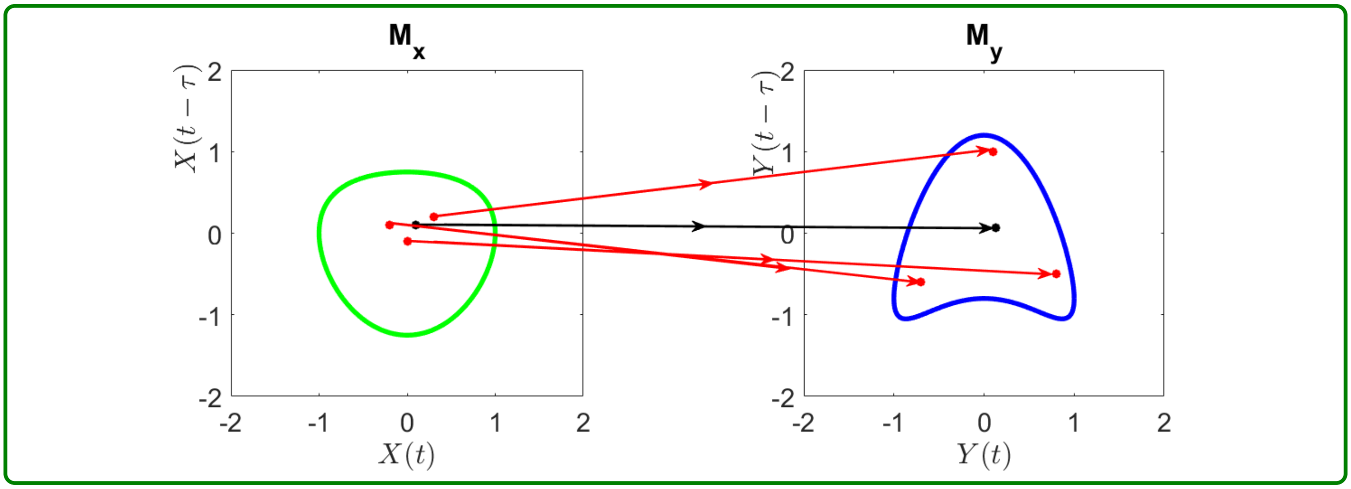

Supplementary Figure 2: **Cross-mapping from $\mathbf{M}_x$ to $\mathbf{M}_y$** when there is no causation from X to Y. In this case, the nearest neighbors of $\mathbf{x}_t$ (the black dot in $\mathbf{M}_x$) are not mapped to the nearest neighbors of $\mathbf{y}_t$ (the black dot in $\mathbf{M}_y$).

1.2 Illustration of the Cross-Mapping Concept using Shadow Manifolds Generated from Resting-State fMRI Data

In this subsection, we choose one subject randomly (Subject 1115) from the rrAD dataset [1], and illustrate the concept of cross-mapping using resting-state fMRI data corresponding to brain regions in the default mode network (DMN). Here, the dimension of the shadow manifold is $E = 3$, and the simplex that represents the neighborhood of $\mathbf{x}_t$ consists of four nearest neighbors, similarly for the neighborhood of $\mathbf{y}_t$.

Supplementary Figure 3 shows the cross-mapping corresponding to two causally coupled regions, right posterior cingulum (X) and left posterior cingulum (Y). As can be seen, in this case, nearest neighbors of a point $\mathbf{x}_t$ in $\mathbf{M}_x$ are mapped to nearest neighbors of point $\mathbf{y}_t$ in $\mathbf{M}_y$ and vice versa. Supplementary Figure 4 shows the cross-mapping corresponding to another two regions, lateral posterior parahippocampal gyrus/parahippocampal place area left (X) and medial frontal gyrus/medial orbital gyrus right (Y). In this case, there is a weak causation from X to Y and no causation from Y to X. As can be seen, nearest neighbors of a point $\mathbf{x}_t$ in $\mathbf{M}_x$ are no longer mapped to nearest neighbors of point $\mathbf{y}_t$ in $\mathbf{M}_y$, and the situation is even worse in the other direction which shows no causation.

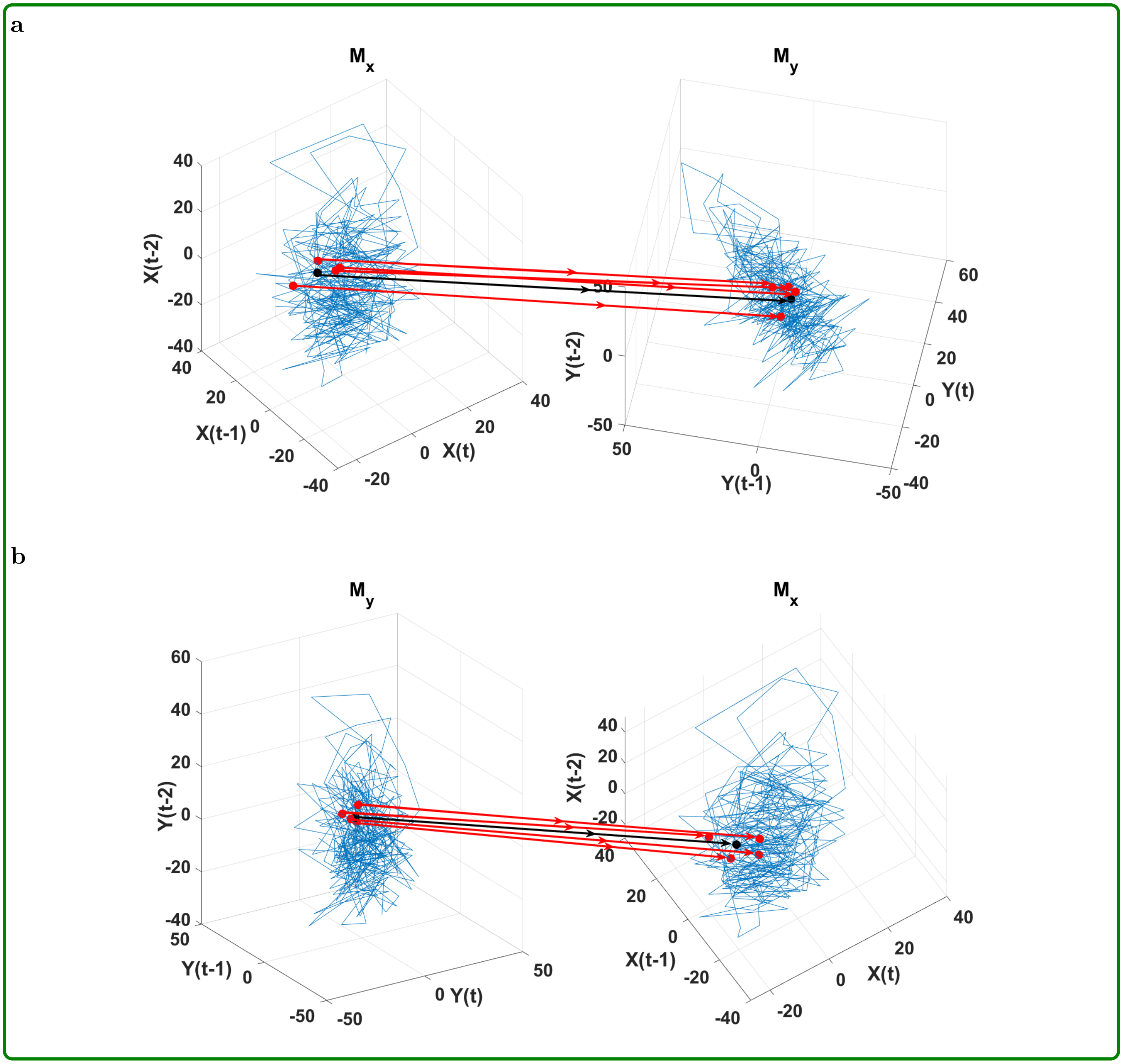

Supplementary Figure 3: **Cross-mapping for Subject 1115: strong causation.** Here X and Y represent the fMRI BOLD signals observed at region 1 (posterior cingulum right) and region 2 (posterior cingulum left), respectively. For these two regions, $\rho_{\text{cCCM}}(X \to Y) = 0.8677$, $\rho_{\text{cCCM}}(Y \to X) = 0.8671$ (here the data length $n = 284$), which indicates that they are cuasally coupled. As can be seen, in this case, nearest neighbors of a point $\mathbf{x}_t$ (the black dot) in $\mathbf{M}_x$ are mapped to nearest neighbors of point $\mathbf{y}_t$ (the black dot) in $\mathbf{M}_y$ and vice versa.

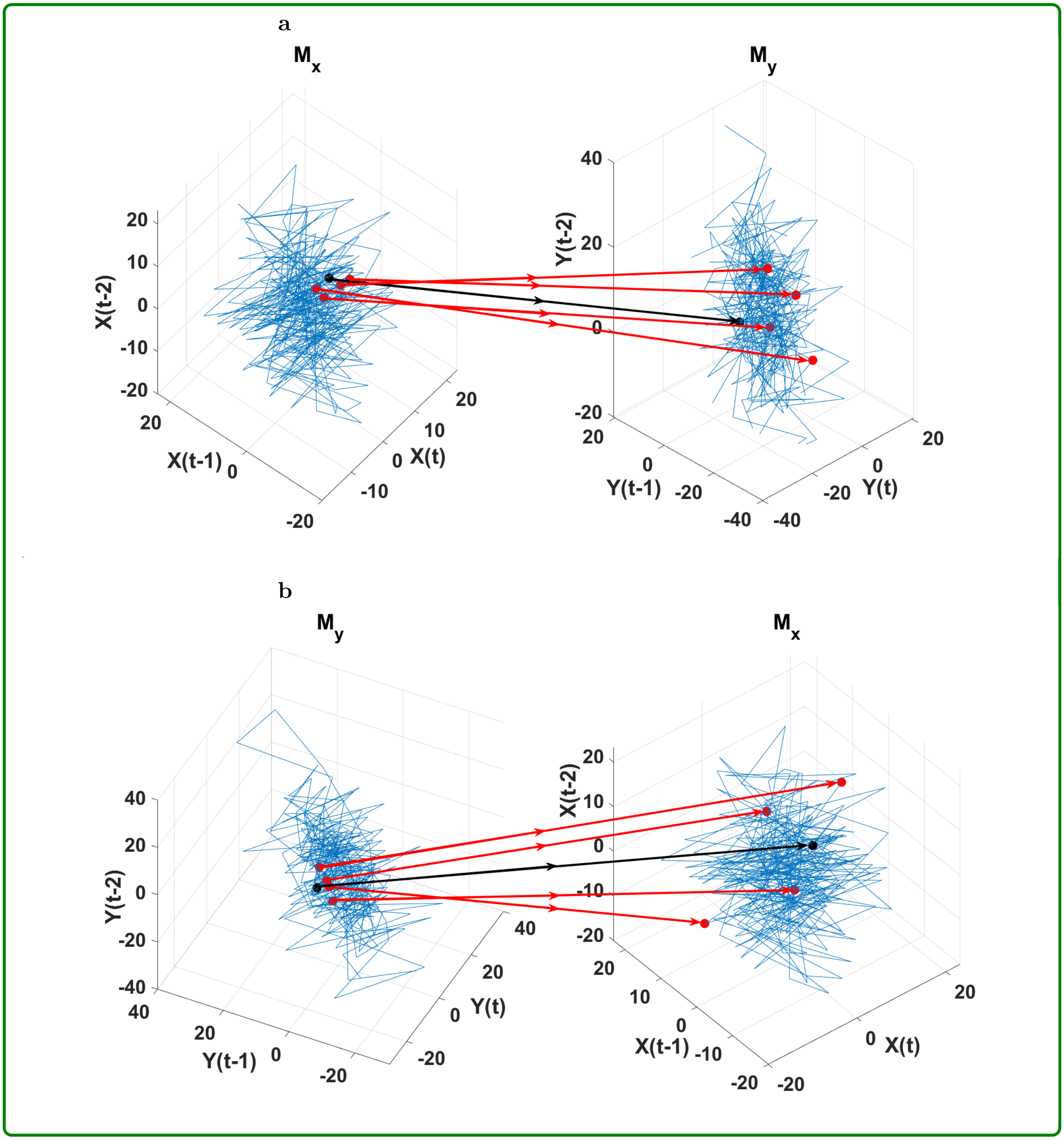

Supplementary Figure 4: **Cross-mapping for Subject 1115: weak or no causation.** Here X and Y represent the fMRI BOLD signals observed at region 16 (lateral posterior parahippocampal gyrus/parahippocampal place area left) and region 5 (medial frontal gyrus/medial orbital gyrus right), respectively. For these two regions, $\rho_{\mathrm{cCCM}}(X \rightarrow Y) = 0.2357$, $\rho_{\mathrm{cCCM}}(Y \rightarrow X) = 0.0682$ (here the data length $n = 284$), which indicates that there is a weak causation effect from X to Y and there is no causation from Y to X. As can be seen, nearest neighbors of a point $\mathbf{x}_t$ (the black dot) in $\mathbf{M}_x$ are no longer mapped to nearest neighbors of point $\mathbf{y}_t$ (the black dot) in $\mathbf{M}_y$, and the situation is even worse in the other direction which shows no causation.

2 Information Measures and Chain Rules

2.1 Information Measures

Let X, Y, Z be three random variables, with samples spaces Ω_x, Ω_y and Ω_z, respectively. Following please find the definitions of the information measures [4] used in this article.

Entropy. The entropy $H(X)$ of a random variable X is defined by

$$H(X) = -\sum_{x \in \Omega_x} p(x) \log p(x). \tag{1}$$

Joint Entropy The joint entropy $H(X, Y)$ of a pair of random variables X and Y is defined by

$$H(X, Y) = -\sum_{x \in \Omega_x, y \in \Omega_y} p(x, y) \log p(x, y). \tag{2}$$

Conditional Entropy. For random variables X and Y, the conditional entropy of X given Y is defined by

$$H(Y|X) = -\sum_{x \in \Omega_x, y \in \Omega_y} p(x, y) \log p(y|x). \tag{3}$$

Mutual Information. For random variables X and Y, the mutual information between X and Y is defined by

$$I(X; Y) = \sum_{x \in \Omega_x, y \in \Omega_y} p(x, y) \log \frac{p(x, y)}{p(x)p(y)}. \tag{4}$$

As can be seen, $I(X; Y) = I(Y; X)$.

Conditional Mutual Information. For random variables X, Y and Z, the mutual information between X and Y conditioning on Z is defined by

$$I(X; Y|Z) = \sum_{x \in \Omega_x, y \in \Omega_y, z \in \Omega_z} p(x, y, z) \log \frac{p(x, y|z)}{p(x|z)p(y|z)}. \tag{5}$$

2.2 Chain Rules

Let $\mathbf{X}^n = [X_1, X_2, \cdots, X_n]$ and $\mathbf{Y}^n = [Y_1, Y_2, \cdots, Y_n]$ denote the time series corresponding to signals X and Y, respectively. The chain rules [4] used in this article are presented below.

Chain Rule for Entropy

$$H(X_1, X_2, ..., X_n) = \sum_{i=1}^{n} H(X_i|X_1, X_2, ..., X_{i-1}). \tag{6}$$

That is,

$$H(\mathbf{X}^n) = \sum_{i=1}^{n} H(X_i|\mathbf{X}^{i-1}), \tag{7}$$

where $\mathbf{X}^i = [X_1, X_2, \cdots, X_i]$.

Chain Rule for Conditional Entropy.

$$H(X_1, X_2, ..., X_n|Y) = \sum_{i=1}^{n} H(X_i|X_1, X_2, ..., X_{i-1}, Y). \tag{8}$$

That is,

$$H(\mathbf{X}^n|Y) = \sum_{i=1}^{n} H(X_i|\mathbf{X}^{i-1}, Y) \tag{9}$$

Chain Rule for Mutual Information

$$I(X_1, X_2, ..., X_n; Y) = \sum_{i=1}^{n} I(X_i; Y|X_1, X_2, ..., X_{i-1}). \tag{10}$$

That is,

$$I(\mathbf{X}^n; Y) = \sum_{i=1}^{n} I(X_i; Y|\mathbf{X}^{i-1}) \tag{11}$$

3 Some Representative Causality Analysis Frameworks: GC, DI, TE and DCM

Recall that $\mathbf{X}^n = [X_1, X_2, \cdots, X_n]$ and $\mathbf{Y}^n = [Y_1, Y_2, \cdots, Y_n]$ denote the time series corresponding to signals X and Y, respectively. The mathematical definitions of Granger Causality (GC), Directed Information (DI), Transfer Entropy (TE) and Dynamic Causal Modeling (DCM) are presented below.

Granger Causality (GC, 1969) The most commonly used method in Granger Causality [5,6] analysis is to compare the following two prediction errors e_i and $\tilde{e_i}$:

$$Y_i = \sum_{j=1}^{K} a_j Y_{i-j} + e_i$$

$$Y_i = \sum_{j=1}^{K} b_j Y_{i-j} + \sum_{j=1}^{L} c_j X_{i-j} + \tilde{e}_i$$

If $\tilde{e_i}$ is much smaller than e_i, that is, the introduction of the previous values of X can improve the accuracy in predicting Y, then we say there is a Granger causal relationship between X and Y. The Granger causality is defined to be the log-likelihood ratio

$$\mathcal{F}_{\mathbf{X}^n \to \mathbf{Y}^n} = \ln \frac{|\mathrm{cov}(\mathbf{e})|}{|\mathrm{cov}(\tilde{\mathbf{e}})|},$$

where $\mathbf{e} = [e_1, e_2, ..., e_n]^T$, $\tilde{\mathbf{e}} = [\tilde{e}_1, \tilde{e}_2, ..., \tilde{e}_n]^T$, and $|\mathrm{cov}(\cdot)|$ stands for the determinant of the covariance matrix.

Directed Information (DI, 1990) The DI from $\mathbf{X}^n$ to $\mathbf{Y}^n$ is defined as [7]:

$$I(\mathbf{X}^n \to \mathbf{Y}^n) = \sum_{i=1}^{n}[H(Y_i|\mathbf{Y}^{i-1}) - H(Y_i|\mathbf{Y}^{i-1}, \mathbf{X}^i)]$$
$$= \sum_{i=1}^{n} I(\mathbf{X}^i; Y_i|\mathbf{Y}^{i-1})$$

The average DI from X to Y, measured in bits per sample is defined as

$$\bar{I}_n(X \to Y) = \frac{I(\mathbf{X}^n \to \mathbf{Y}^n)}{n}$$

Transfer Entropy (TE, 2000) The transfer entropy [8] from X to Y is defined as:

$$\begin{aligned} &T_{X\to Y}(K, L, i) \\ &= H(Y_i|Y_{i-K\,:\,i-1}) - H(Y_i|Y_{i-K\,:\,i-1}, X_{i-L\,:\,i-1}) \\ &= I(X_{i-L\,:\,i-1}; Y_i|Y_{i-K\,:\,i-1}) \end{aligned}$$

where H denotes the entropy operator, $Y_{i-K\,:\,i-1} = [Y_{i-K}, ..., Y_{i-1}]$, and $X_{i-L\,:\,i-1} = [X_{i-L}, ..., X_{i-1}]$, and I is the mutual information operator. Its cumulative variant is generally defined as

$$T(\mathbf{X}^n \to \mathbf{Y}^n) = \sum_{i=2}^{n} T_{X\to Y}(i-1, i-1, i)$$
$$= \sum_{i=2}^{n} I(\mathbf{X}^{i-1}; Y_i|\mathbf{Y}^{i-1})$$

The average TE from X to Y, measured in bits per sample is defined as

$$T(X \to Y) = \frac{T(\mathbf{X}^n \to \mathbf{Y}^n)}{n-1}$$

Dynamic Causal Modeling (DCM, 2003) We illustrate DCM [9] using its discrete case. In DCM, the neurostate $\mathbf{S}$ (generally considered to be the neuronal activity), the external input U, the connectivity matrix A that describes the connections among brain regions, matrix B that describes the input strengths, the observed BOLD signal $\mathbf{Z}$ and the independent noise are formulated as a complex dynamic system, characterized as:

$$\mathbf{S}(k+1) = A\mathbf{S}(k) + BU(k) + \mathbf{\Omega}_1(k)$$
$$\mathbf{Z}(k) = \sum_{m=0}^{M} \Lambda(m)\mathbf{S}(k-m) + \mathbf{\Omega}_2(k)$$

where $\mathbf{\Omega}_1(k)$ and $\mathbf{\Omega}_2(k)$ denote the state noise and observation noise, respectively. If we only consider two regions such that $\mathbf{S} = [X, Y]^T$, and combine the external input and noise into one item, the discrete DCM model reduces to:

$$\begin{bmatrix} X(k+1) \\ Y(k+1) \end{bmatrix} = \begin{bmatrix} A_{11} & A_{12} \\ A_{21} & A_{22} \end{bmatrix} \begin{bmatrix} X(k) \\ Y(k) \end{bmatrix} + \begin{bmatrix} \Omega_{11}(k) \\ \Omega_{12}(k) \end{bmatrix}$$

Here coefficients A_{12} and A_{21} reflects the causal relationship between X and Y. More specifically, if $|A_{21}| > |A_{12}|$, then X is likely to be the casual side; otherwise, Y is the causal side.

4 The Approximate Equivalence between cCCM and DI for Gaussian Variables

In this section, we first revisit the result of Gel'fand [10] where he established the closed-form relationship of mutual information and Pearson correlation for Gaussian variables. We then show the approximate equivalence of cCCM and DI following Takens' theorem [3] and the result of Gel'fand [10] as well as the Shannon–McMillan–Breiman theorem [13].

4.1 Closed-Form Relationship between Pearson Correlation and Mutual Information

Mutual information (MI) is an information-theoretic metric that measures both linear and nonlinear dependence between two random variables.

Let X and Y be two random variables, then the mutual information between them is defined as

$$I(X;Y) = H(X) - H(X|Y), \tag{12}$$

where $H(X)$ is the entropy of X and $H(X|Y)$ is the conditional entropy. Let Ω_x and Ω_y denote the sample space of X and Y, respectively; let $P_Y(y) = Pr\{Y = y\}$, and $P_{X|Y}(x|y) = Pr\{X = x|Y = y\}$, then

$$\begin{aligned} H(X|Y) &= -\sum_{y\in\Omega_y} P_Y(y) \sum_{x\in\Omega_x} P_{X|Y}(x|y) \log P_{X|Y}(x|y) &(13)\\ &= -\sum_{y\in\Omega_y}\sum_{x\in\Omega_x} P_{X,Y}(x,y) \log\left[\frac{P_{X,Y}(x,y)}{P_Y(y)}\right], &(14)\end{aligned}$$

where $P_{X,Y}(X,Y) = Pr\{X = x, Y = y\}$ is the joint probability. The physical meaning of $H(X|Y)$ is the average uncertainty or information left in X per message after Y is observed. This implies that the mutual information:

$I(X;Y) =$ the total uncertainty in X − the uncertainty left in X after Y is observed.

That is, the physical meaning of the mutual information is "the average uncertainty removed about X per message after Y is observed." For this reason, in communications, the mutual information $I(X,Y)$ is also explained as the information which is successfully transmitted or passed (per message on average) through the channel between X and Y. It can be shown that

$$I(X;Y) = I(Y;X) = H(X) + H(Y) - H(X,Y). \tag{15}$$

In general, Pearson correlation represents the linear dependence between two random variables, while mutual information characterizes both linear and non-linear dependence between them. However, closed-form relationship between Pearson correlation and mutual information can be derived under certain conditions. More specifically, when X and Y follow normal distributions and their joint distribution is bivariate normal, that is $X \sim \mathcal{N}(\mu_x, \sigma_x^2)$, $Y \sim \mathcal{N}(\mu_y, \sigma_y^2)$ and

$$\begin{pmatrix} X \\ Y \end{pmatrix} \sim \mathcal{N}\left(\begin{pmatrix} \mu_x \\ \mu_y \end{pmatrix}, \Sigma\right), \qquad \Sigma = \begin{pmatrix} \sigma_x^2 & \rho\sigma_x\sigma_y \\ \rho\sigma_x\sigma_y & \sigma_y^2 \end{pmatrix}$$

then it can be proved that (Gel'fand,1957 [10])

$$I(X;Y) = -\frac{1}{2}\log(1-\rho^2), \tag{16}$$

where ρ is the ensemble average representation of the Pearson correlation between X and Y. In fact,

$$H(X) = \frac{1}{2}\log(2\pi e\sigma_x^2), \qquad H(Y) = \frac{1}{2}\log(2\pi e\sigma_y^2), \tag{17}$$

$$\begin{aligned} H(X,Y) &= \frac{1}{2}\log[(2\pi e)^2|\Sigma|] \\ &= \frac{1}{2}\log[(2\pi e)^2(1-\rho^2)\sigma_x^2\sigma_y^2]. \end{aligned} \tag{18}$$

Equation (16) then follows from equations (15),(17) and (18).

The result above was extended to vector case in 2012 [11,12] by Arellano-Valle et al.

4.2 Approximate Equivalence between Causalized-CCM and DI

Theorem 1. *Let X, Y be two dynamically coupled zero-mean Gaussian random variables which are also bivariate Gaussian and share the same attractor manifold $\boldsymbol{M}$. $\boldsymbol{X}^n = \{X_1, \cdots, X_n\}$, $\boldsymbol{Y}^n = \{Y_1, \cdots, Y_n\}$ are the time series contain the samples of X, Y, respectively, and are are stationary ergodic random processes. Define*

$$I(\boldsymbol{X}^n \to \boldsymbol{Y}^n) = \sum_{i=1}^{n}[H(Y_i|\,\boldsymbol{Y}^{i-1}) - H(Y_i|\,\boldsymbol{Y}^{i-1}, \boldsymbol{X}^i)] = \sum_{i=1}^{n} I(\boldsymbol{X}^i; Y_i|\,\boldsymbol{Y}^{i-1}). \tag{19}$$

$$\bar{I}_n(X \to Y) = \frac{I(\boldsymbol{X}^n \to \boldsymbol{Y}^n)}{n}, \quad \bar{I}_n(Y;\hat{Y}) = \frac{I(\boldsymbol{Y}^n;\hat{\boldsymbol{Y}}^n)}{n}. \tag{20}$$

Then

$$\lim_{n\to\infty} \bar{I}_n(X \to Y) = \lim_{n\to\infty} \bar{I}_n(Y;\hat{Y}), \tag{21}$$

and when n is sufficiently large,

$$\bar{I}_n(X \to Y) \approx \bar{I}_n(Y;\hat{Y}) \approx -\frac{1}{2}\log(1 - \rho^2_{cCCM}(\boldsymbol{X}^n \to \boldsymbol{Y}^n)), \tag{22}$$

where $\rho_{cCCM}(\boldsymbol{X}^n \to \boldsymbol{Y}^n) = \rho\left(\mathbf{Y}^n, \hat{\mathbf{Y}}^n\right)$. The result also holds in the reverse direction.

Now, we can prove the approximate equivalence of causalized CCM and DI under Gaussian variables in two steps.

Step 1: Show that: if the two signals X and Y are dynamically coupled, then

$$\lim_{n\to\infty} \bar{I}_n(X \to Y) = \lim_{n\to\infty} \bar{I}_n(Y;\hat{Y}) \tag{23}$$

where

$$\bar{I}_n(X \to Y) = \frac{I(\mathbf{X}^n \to \mathbf{Y}^n)}{n}, \qquad \bar{I}_n(Y;\hat{Y}) = \frac{I(\mathbf{Y}^n;\hat{\mathbf{Y}}^n)}{n},$$

and

$$\hat{Y}(t)|\mathbf{M}_x = \sum_{i=1}^{E+1} w_i Y(t_i), \qquad t = 1, \cdots, n, \tag{24}$$

is the estimated value of $Y(t)$ in causalized-CCM, here $t_i < t$ and

$$w_i = \frac{u_i}{\sum_{j=1}^{E+1} u_j}, \quad \text{with} \quad u_i = exp\{-\frac{d(\mathbf{x}(t), \mathbf{x}(t_i))}{d(\mathbf{x}(t), \mathbf{x}(t_1))}\}.$$

In fact, recall that the directed information from time series $\mathbf{X}^n$ to $\mathbf{Y}^n$ is defined as

$$\begin{aligned} I(\mathbf{X}^n \to \mathbf{Y}^n) &= \sum_{i=1}^{n} [H(Y_i|\mathbf{Y}^{i-1}) - H(Y_i|\mathbf{Y}^{i-1}, \mathbf{X}^i)] \\ &= \sum_{i=1}^{n} I(\mathbf{X}^i; Y_i|\mathbf{Y}^{i-1}) \end{aligned}$$

and the average DI from X to Y, measured in bits per sample, is defined as

$$\bar{I}_n(X \to Y) = \frac{I(\mathbf{X}^n \to \mathbf{Y}^n)}{n}. \tag{25}$$

The mutual information between $\mathbf{Y}^n$ and $\hat{\mathbf{Y}}^n$ is given by

$$\begin{aligned} I(\mathbf{Y}^n; \hat{\mathbf{Y}}^n) &= H(\mathbf{Y}^n) - H(\mathbf{Y}^n|\hat{\mathbf{Y}}^n) \\ &= \sum_{i=1}^{n} [H(Y_i|\mathbf{Y}^{i-1}) - H(Y_i|\mathbf{Y}^{i-1}, \hat{\mathbf{Y}}^n)] \\ &= \sum_{i=1}^{n} [H(Y_i|\mathbf{Y}^{i-1}) - H(Y_i|\mathbf{Y}^{i-1}, \mathbf{X}^i)] + \sum_{i=1}^{n} [H(Y_i|\mathbf{Y}^{i-1}, \mathbf{X}^i) - H(Y_i|\mathbf{Y}^{i-1}, \hat{\mathbf{Y}}^i)] \\ &+ \sum_{i=1}^{n} [H(Y_i|\mathbf{Y}^{i-1}, \hat{\mathbf{Y}}^i) - H(Y_i|\mathbf{Y}^{i-1}, \hat{\mathbf{Y}}^n)] \end{aligned} \tag{26}$$

Define $a_i = H(Y_i|\mathbf{Y}^{i-1}, \mathbf{X}^i) - H(Y_i|\mathbf{Y}^{i-1}, \hat{\mathbf{Y}}^i)$ and $b_i = H(Y_i|\mathbf{Y}^{i-1}, \hat{\mathbf{Y}}^i) - H(Y_i|\mathbf{Y}^{i-1}, \hat{\mathbf{Y}}^n)$, then it follows that

$$I(\mathbf{Y}^n; \hat{\mathbf{Y}}^n) = I(\mathbf{X}^n \to \mathbf{Y}^n) + \sum_{i=1}^{n} a_i + \sum_{i=1}^{n} b_i \tag{27}$$

Model Y as

$$Y_i = \hat{Y}_i + e_i, \tag{28}$$

where $\hat{Y}_i = \hat{Y}(i)$ is defined in equation (24). Without loss of generality, we can assume that e_i is independent of $\hat{Y}_i$, and has zero-mean and variance $\sigma_{e_i}^2$. Following the argument in Takens' theorem [3], as $i \to \infty$, the shadow manifolds $\mathbf{M}_x$ and $\mathbf{M}_y$ become denser and the neighborhood shrinks such that $\hat{Y}_i \to Y_i$. It then follows that $\lim_{i\to\infty} \sigma_{e_i}^2 = 0$. Note that

$$0 \le H(Y_i|\mathbf{Y}^{i-1}, \mathbf{X}^i) \le H(e_i) \le \frac{1}{2}\log(2\pi e \sigma_{e_i}^2), \tag{29}$$

here $e \approx 2.71828$ is the Euler's number. It then follows that

$$H(Y_i|\mathbf{Y}^{i-1}, \mathbf{X}^i) \to 0 \quad \text{as} \quad i \to \infty. \tag{30}$$

Similarly,

$$H(Y_i|\mathbf{Y}^{i-1}, \hat{\mathbf{Y}}^i) \to 0, \qquad H(Y_i|\mathbf{Y}^{i-1}, \hat{\mathbf{Y}}^n) \to 0 \quad \text{as} \quad i \to \infty. \tag{31}$$

Following the definition of a_i and b_i, we have

$$a_i \to 0, \quad b_i \to 0 \ \text{ as } \ i \to \infty. \tag{32}$$

Define

$$\bar{I}_n(Y;\hat{Y}) = \frac{I(\mathbf{Y}^n;\hat{\mathbf{Y}}^n)}{n}, \tag{33}$$

it then follows from equation (27)that

$$\frac{I(\mathbf{Y}^n;\hat{\mathbf{Y}}^n)}{n} = \frac{I(\mathbf{X}^n \to \mathbf{Y}^n)}{n} + \frac{1}{n}\sum_{i=1}^{n} a_i + \frac{1}{n}\sum_{i=1}^{n} b_i \tag{34}$$

Note that $a_i \to 0, \quad b_i \to 0 \ \text{ as } \ i \to \infty$, it follows that

$$\frac{1}{n}\sum_{i=1}^{n} a_i \to 0, \qquad \frac{1}{n}\sum_{i=1}^{n} b_i \to 0 \ \text{ as } \ n \to \infty \tag{35}$$

That is,

$$\lim_{n\to\infty} \bar{I}_n(X \to Y) = \lim_{n\to\infty} \bar{I}_n(Y;\hat{Y}).$$

This implies that when n is sufficiently large,

$$\bar{I}_n(X \to Y) \approx \bar{I}_n(Y;\hat{Y}). \tag{36}$$

Step 2: Show that: if Y and $\hat{Y}$ are Gaussian random variables and their joint distribution is bivariate Gaussian, then when n is sufficiently large,

$$\bar{I}_n(X \to Y) \approx \bar{I}_n(Y;\hat{Y}) \approx -\frac{1}{2}\log(1 - \rho^2_{\mathrm{cCCM}}(X \to Y)), \tag{37}$$

where $\rho_{\mathrm{cCCM}}(X \to Y) = \rho(\mathbf{Y}^n, \hat{\mathbf{Y}}^n)$.

This can be obtained from the closed-form relationship between Pearson correlation and mutual information. Let $I(Y;\hat{Y})$ and $\rho(Y,\hat{Y})$ denote mutual information and Pearson correlation between Y and $\hat{Y}$, respectively. If Y and $\hat{Y}$ are Gaussian random variables and their joint distribution is bivariate Gaussian, based on Gelfand's result in equation (16), we have

$$I(Y;\hat{Y}) = -\frac{1}{2}\log(1 - \rho^2(Y,\hat{Y})). \tag{38}$$

Recall that the mutual information rate $\bar{I}_n(Y;\hat{Y}) = \frac{I(\mathbf{Y}^n;\hat{\mathbf{Y}}^n)}{n}$ represents the average mutual information between $\mathbf{Y}^n$ and $\hat{\mathbf{Y}}^n$, measured in bits per sample, and each sample in $\mathbf{Y}^n$ can be regarded as a message emitted from the memoryless random source Y, and each sample in $\hat{\mathbf{Y}}^n$ is emitted from $\hat{Y}$. On the other hand, $I(Y;\hat{Y})$, is the average information (virtually) "transmitted" between Y and $\hat{Y}$, measured in bits per sample. That is, they have the same physical meaning. As $n \to \infty$, both $\mathbf{Y}^n$ and $\hat{\mathbf{Y}}^n$ are stationary ergodic random processes, following the Shannon–McMillan–Breiman theorem [13], we have

$$\bar{I}_n(Y;\hat{Y}) \to I(Y;\hat{Y}), \quad \text{as} \ \ n \to \infty. \tag{39}$$

Similarly,

$$\rho(\mathbf{Y}^n, \hat{\mathbf{Y}}^n) \to \rho(Y,\hat{Y}), \quad \text{as} \ \ n \to \infty, \tag{40}$$

we can then get equation (37) from equations (36), (38)-(40).

The other direction: Repeat Step 1 and Step 2 in the reverse direction, we can show that if X and Y are causally coupled, X and $\hat{X}$ are Gaussian random variables and their joint distribution is bivariate Gaussian, then when n is sufficiently large,

$$\bar{I}_n(Y \to X) \approx \bar{I}_n(X; \hat{X}) \approx -\frac{1}{2}\log(1 - \rho^2_{\text{cCCM}}(Y \to X)), \tag{41}$$

where $\rho_{\text{cCCM}}(Y \to X) = \rho(\mathbf{X}^n, \hat{\mathbf{X}}^n)$.

5 Multivariate Conditional cCCM

In this section, we first provide a simulation example to illustrate the concept of conditional causality, and then present the multivariate KNN search based conditional cCCM.

5.1 A Simulation Example on Conditional Causality

The following example illustrates that X and Y may have high correlation and causation, but very low conditional correlation and causation when the third variable is taken into consideration.

Let $randn(1, N)$ represent a Gaussian random sequence of length N, with zero-mean and unit variance. Let $X_0 = randn(1, 500), Y_0 = randn(1, 500)$ and $Z = t^2 + 1$ where $t = [0.01, 0.02, \cdots, 5]$. Consider

$$X = X_0 + Z \text{ and } Y = Y_0 + Z. \tag{42}$$

We have that the Pearson correlation is $\rho(X, Y) = 0.9813$, and CCM causality $\rho_{\text{cCCM}}(X \to Y) = 0.9876$. However, when the impact of Z is considered, the conditional Pearson correlation is $\rho(X, Y|Z) = -0.0579$, and the conditional CCM causality $\rho_{\text{cCCM}}(X \to Y|Z) = -0.0464$. That is, X and Y are not really coupled.

This example illustrates the impact of the third party or additional random variable(s) on the causal relationship between two random variables, and calls for a more systematic study on multivariate conditional cCCM.

5.2 Multivariate KNN search based conditional cCCM

This method aligns with the multivariate KNN predictability approaches [14–17] (Porta et al., PLOS ONE 2014, Proceedings of the IEEE 2016 and Entropy 2023, Abarbanel et al. Phys Rev, 1994).

Let $\mathbf{\Omega} = \{X_1, \cdots, X_Q\}$ be the set of dynamically coupled random variables which share the same attractor manifold $\mathbf{M}$. For $q = 1, \cdots, Q$, let $\mathbf{X}_q^n = [X_{q,1}, X_{q,2}, \cdots, X_{q,n}]$ denotes the time series consisting of samples of $\mathbf{X}_q$ and construct the shadow manifolds with respect to $\mathbf{X}_q^n$ as

$$\mathbf{M}_{X_q} = \{\mathbf{x}_{q,t} \mid \mathbf{x}_{q,t} = [X_{q,t}, X_{q,t-\tau}, \cdots, X_{q,t-(E-1)\tau}],\ t = 1 + (E-1)\tau, \cdots, n\},\quad q = 1, \cdots, Q. \tag{43}$$

For $q = 1, \cdots, Q$, let $\hat{X}_{i,t}|\mathbf{M}_{X_q}$ be the estimated $X_{i,t}$ based on $\mathbf{M}_{X_q}$. Let $\hat{X}_{i,t}|\mathbf{\Omega}$ denote the multivariate prediction of $X_{i,t}$ based on all the $\hat{X}_{i,t}|\mathbf{M}_{X_q}$, $q = 1, \cdots, Q, q \neq i$. That is,

$$\hat{X}_{i,t}|\mathbf{\Omega} = \sum_{q=1, q\neq i}^{Q} a_q \hat{X}_{i,t}|\mathbf{M}_{X_q} + e_{i,t}|\mathbf{\Omega}, \tag{44}$$

where the coefficients $a_q, q = 1, \cdots, i-1, i+1, \cdots, Q$, are selected to minimize the variance of the estimation error $e_{i,t}|\mathbf{\Omega}$. Similarly, $\hat{X}_{i,t}|\mathbf{\Omega} \setminus \{X_j\}$ denotes the multivariate prediction of $X_{i,t}$ based on all the $\hat{X}_{i,t}|\mathbf{M}_{X_q}$,

$q = 1, \cdots, Q$ but $q \neq i, j$, that is

$$\hat{X}_{i,t}|\mathbf{\Omega} \setminus \{X_j\} = \sum_{q=1, q\neq i,j}^{Q} b_q \hat{X}_{i,t}|\mathbf{M}_{X_q} + e_{i,t}|\mathbf{\Omega} \setminus \{X_j\}, \tag{45}$$

where the coefficients $b_q, q \in \{1, \cdots, Q\}$ and $q \neq i, j$, are selected to minimize the variance of the estimation error $e_{i,t}|\mathbf{\Omega} \setminus \{X_j\}$. Following equations (44),(45), define the estimation error vectors as:

$$\mathbf{e}_i^n|\mathbf{\Omega} = [\ e_{i,1}|\mathbf{\Omega}, \cdots, e_{i,n}|\mathbf{\Omega}\ ],$$
$$\mathbf{e}_i^n|\mathbf{\Omega} \setminus \{X_j\} = [\ e_{i,1}|\mathbf{\Omega} \setminus \{X_j\}, \cdots, e_{i,n}|\mathbf{\Omega} \setminus \{X_j\}\ ].$$

Following [15], the causality ratio (CR) from $X_j \to X_i$ is defined as

$$\mathrm{CR}_{X_j \to X_i} = \frac{\mathrm{Var}(\mathbf{e}_i^n|\mathbf{\Omega} \setminus \{X_j\}) - \mathrm{Var}(\mathbf{e}_i^n|\mathbf{\Omega})}{\mathrm{Var}(\mathbf{e}_i^n|\mathbf{\Omega} \setminus \{X_j\})}.$$

6 Additional Results on Brain Causality Analysis based on Experimental fMRI Data

6.1 Causality Analysis of the Default Mode Network based on Resting-State fMRI Data

We conducted causality analysis of the default mode network (DMN) of the brain using DI and cCCM based on the fMRI data for two randomly selected subjects from the rrAD trial, Subject 1115 and Subject 1151, respectively. Again, we can observe the log relationship between DI and cCCM. The cCCM value versus the data length is illustrated for selected region pairs in Supplementary Figure 5. (d), (e), (f) (for Subject 1115) and Supplementary Figure 6. (d), (e), (f) (for Subject 1151), where each figure corresponds to a particular region pair. Here the total length of the BOLD (blood-oxygen-level-dependent) signal is 284 data points, with a sampling period of 2.5s, i.e., the time duration of the BOLD signal is about 12 minutes. Region pairs that show significantly asymmetric interactions (or say, with significant unidirectional causality), selected as pairs (i,j) where $|\rho_{\mathrm{cCCM}}(i \to j)| - |\rho_{\mathrm{cCCM}}(j \to i)| > 0.15$, are shown in Supplementary Figure 5 (g) for Subject 1115, and Supplementary Figure 6 (g) for Subject 1151. As can be seen, asymmetric interactions (or unidirectional causality) can be observed in individual subjects, however, the region pairs that show obvious unidirectional causality vary across different subjects. When the result is averaged over all the scans, as shown in Figure 3. (C), the DMN network does not present dominant unidirectional causality during the resting-state but does show significant bidirectional causality among regions right posterior cingulum, left Posterior Cingulum, right precuneus/angular gyrus and left precuneus/angular gyrus, where ρ_{cCCM} is bigger or close to 0.5 in bother directions. This result is consistent with the previous findings in literature [18].

The cCCM causation (ρ_{cCCM}) distribution and average node significance in DMN for all the 30 subjects are shown in Supplementary Figure 7. Here node significance as a transmitter and receiver is evaluated using $\sum_{j=1, j\neq i}^{18} |\rho_{\mathrm{cCCM}}(i \to j)|$ and $\sum_{i=1, i\neq j}^{18} |\rho_{\mathrm{cCCM}}(i \to j)|$, respectively, and ρ_{cCCM} for each region pair is averaged over all the 30 subjects. The pattern of bidirectional causal interactions in the averaged ρ_{cCCM} indicates that Posterior cingula of DMN, followed by precuneus/angular gyri, and medial frontal gyri/medial orbital gyri, act as key nodes for both information transmitting and receiving. Our result is consistent with previous findings in [18, 19].

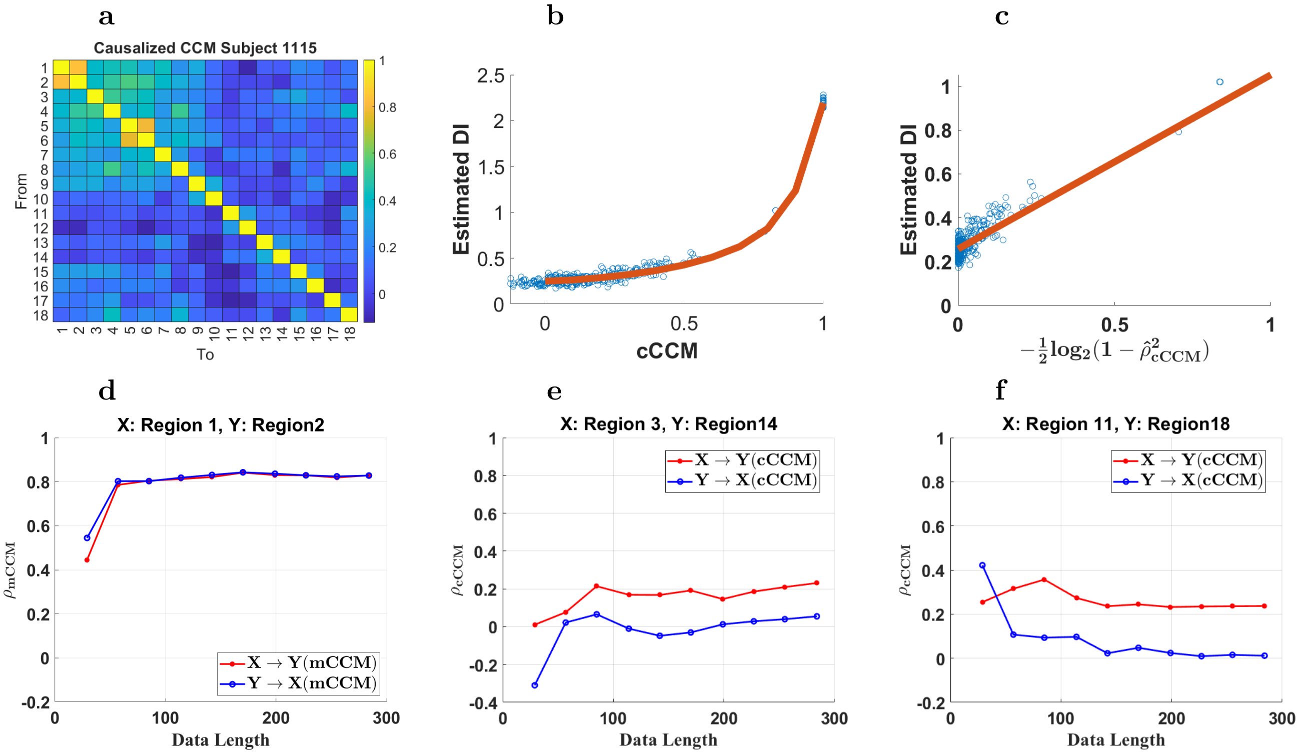

g. Region pairs that show significantly asymmetric interactions, Subject 1115

Region i	Region j	$\rho_{\mathrm{cCCM}}(i \to j)$	$\rho_{\mathrm{cCCM}}(j \to i)$	Pearson
3	14	0.2309	0.0548	0.3063
9	16	0.2132	0.0618	0.3037
11	18	0.2367	0.0114	0.1986
18	3	0.2076	0.0335	0.3244
18	15	0.2562	0.0332	0.2249

Supplementary Figure 5: **Causality analysis for Subject 1115**. a, cCCM causality in DMN. b, The approximate log-relationship between DI & cCCM. c, The approximate linear relationship between DI and cCCM-predicted DI, which is given by $-\frac{1}{2}\log_2(1-\rho^2_{\mathrm{cCCM}}(X \to Y))$. d-f, Illustration of cCCM causation versus fMRI data length for different region pairs. g, Region pairs that show significantly asymmetric interactions, selected as pairs (i,j) where $|\rho_{\mathrm{cCCM}}(i \to j)| - |\rho_{\mathrm{cCCM}}(j \to i)| > 0.15$.

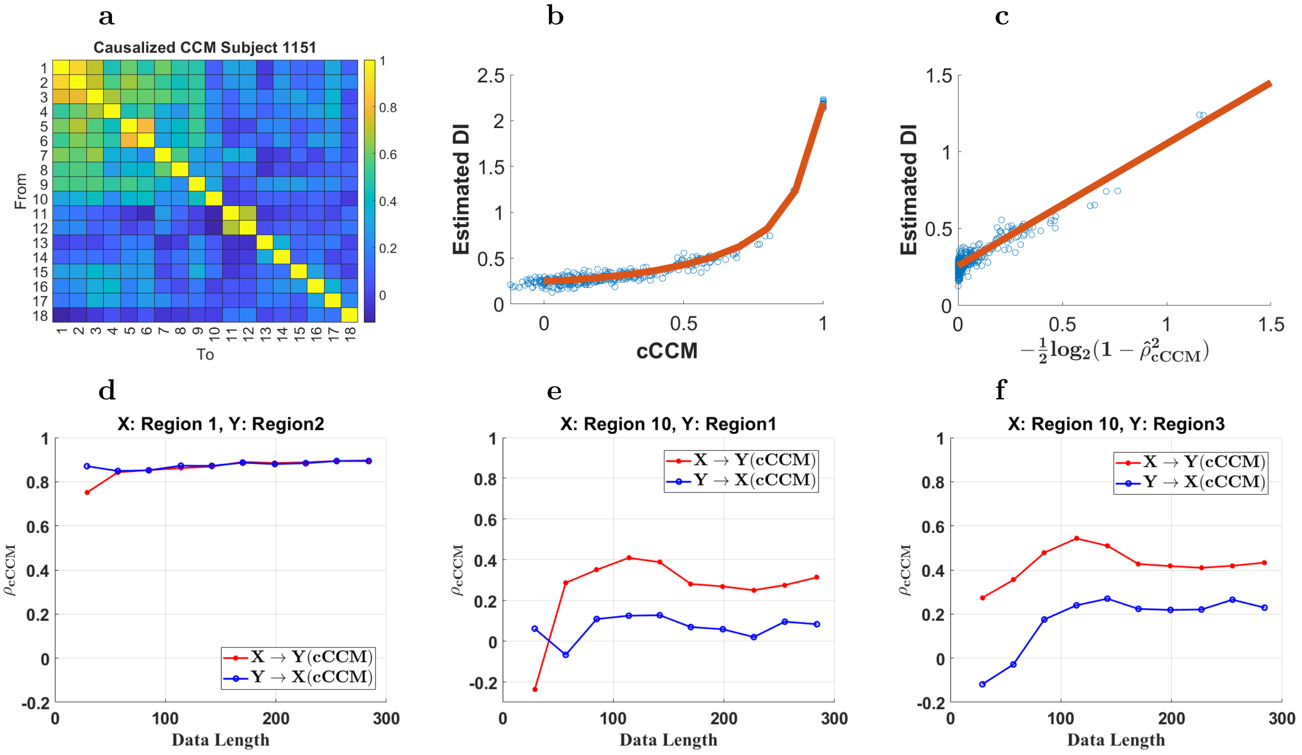

g. Region pairs that show significantly asymmetric interactions, Subject 1151

Region i	Region j	$\rho_{\mathrm{cCCM}}(i \to j)$	$\rho_{\mathrm{cCCM}}(j \to i)$	Pearson
1	17	0.3243	0.1539	0.4063
3	12	0.2535	0.0853	0.2945
10	1	0.3134	0.0835	0.3272
10	2	0.3462	0.0926	0.3561
10	3	0.4333	0.2290	0.4576
10	5	0.3805	0.1776	0.3735
15	1	0.2886	0.1159	0.3483
15	2	0.2973	0.1240	0.3409
15	5	0.2677	0.0728	0.3355
15	6	0.2734	0.0868	0.3259
16	3	0.3398	0.1741	0.4040

Supplementary Figure 6: **Causality analysis for Subject 1151.** a, cCCM causality in DMN. b, The approximate log-relationship between DI & cCCM. c, The approximate linear relationship between DI and cCCM-predicted DI, which is given by $-\frac{1}{2}\log_2(1-\rho^2_{\mathrm{cCCM}}(X \to Y))$. d-f, Illustration of cCCM causation versus fMRI data length for different region pairs. g, Region pairs that show significantly asymmetric interactions, selected as pairs (i,j) where $|\rho_{\mathrm{cCCM}}(i \to j)| - |\rho_{\mathrm{cCCM}}(j \to i)| > 0.15$.

a. Node significance as transmitter

Order	Region Index i	Total Out (cCCM) $\sum_{j=1(j\neq i)}^{18} \lvert\rho_{\text{cCCM}}(i \to j)\rvert$
1	2	5.4646
2	1	5.2728
3	4	4.5156
4	3	4.1896
5	6	4.1602
6	5	3.9994
7	8	3.8308
8	7	3.5408
9	9	3.2851
10	10	2.8044

b. Node significance as receiver

Order	Region Index j	Total In (cCCM) $\sum_{i=1(i\neq j)}^{18} \lvert\rho_{\text{cCCM}}(i \to j)\rvert$
1	2	5.4735
2	1	5.3334
3	4	4.7424
4	3	4.4279
5	6	4.2708
6	5	4.1148
7	8	3.8376
8	7	3.5031
9	9	3.3430
10	10	2.6626

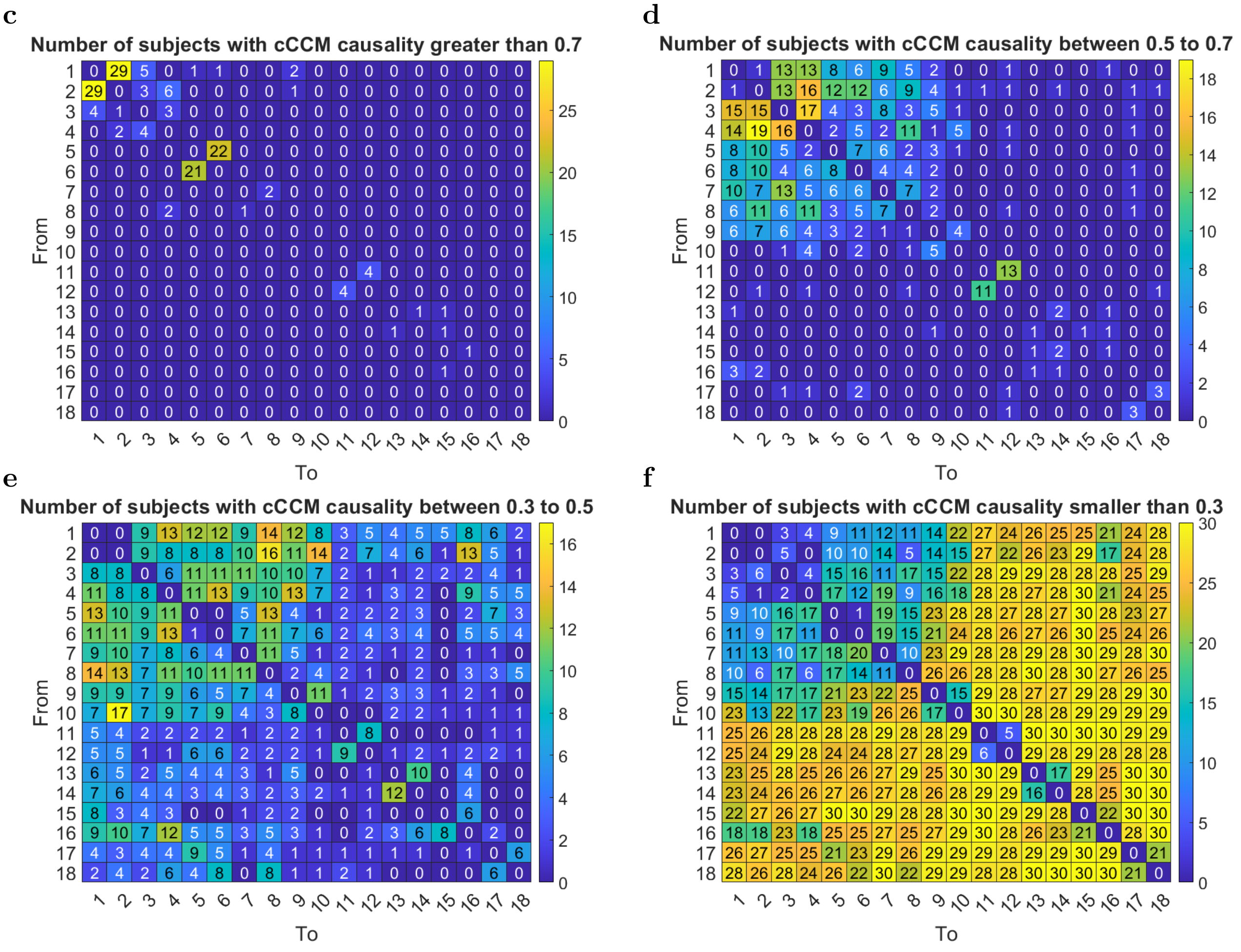

Supplementary Figure 7: **Causality analysis of the Default Mode Network (DMN).** a-b, Node significance as a transmitter or receiver in the default mode network (DMN), averaged over 30 subjects. Here, the pattern of bidirectional causal interactions indicates that Posterior cingula of DMN, followed by Precuneus/Angular Gyri, and Medial Frontal Gyri/Medial Orbital Gyri, act as key nodes for both information transmitting and receiving. c-f, Distribution of cCCM causality in DMN, represented with the number of subjects whose corresponding cCCM value falls into the specified ranges ((0.7,1), (0.5 0.7], (0.3 0.5], (0 0.3]) in each region pair, respectively. These numbers indicate that cCCM causality varies across different region pairs and subjects, and also reflect the significance level of each region in information transmission and reception.

6.2 Unidirectional Causality Analysis of the Brain Network based on Task-Driven fMRI Data

Numerical results based on the task-fMRI data:

Result for bivariate cCCM and CCM: Recall that the total length of the fMRI BOLD time series under visual stimulation condition is $n = 192$, with the sampling period being 2.5s. We conducted causality analysis for all the possible unidirectional regional pairs using both cCCM and DI. However, it was observed that cCCM did not converge with the data length $n = 192$. We chose to interpolate the fMRI sequence by a factor of 2 using the spline interpolation command in Matlab, which reduced the sampling period from 2.5 s to 1.25 s and increased the data length to $(2 \times 192) - 1 = 383$. We then performed cCCM and CCM to the interpolated sequences and obtained *consistent* results on unidirectional causality with DI.

It is worth noting that CCM, cCCM, and DI all identified the same unidirectional causal relations among these ROI pairs, with both the original BOLD sequences and the interpolated sequences. This implies that even if CCM and cCCM did not converge with $n = 192$ samples, they were still able to identify the unidirectional causal relations correctly, but interpolation of the fMRI sequence did enhance the robustness of CCM and cCCM based causality analysis. In literature, it was also reported in Lin et al. 2014 [22] that increasing the sampling rate of the fMRI signal can improve the robustness of causality analysis.

The consistency of cCCM and DI in the detection of unidirectional causality (averaged over all the 14 subjects) is shown in Supplementary Figures 8 and 9. In addition, the causal relations identified by cCCM is also consistent across most subjects, as illustrated in Supplementary Figure 8 (d)-(e). For example, in Supplementary Figure 8 (d), 12 out of 14 subjects showed apparent unidirectional causality from LV1 → LSMC; in Supplementary Figure 8(e), 13 out of 14 subjects showed apparent unidirectional causality from LV1 → LPWM. On the other hand, as shown in Supplementary Figure 8 (f), bidirectional causality exists between LV1 and LPPA, where 8 out 14 subjects showed slight unidirectional causality from LV1 → LPPA, and 6 showed slight unidirectional causality in the opposite direction or almost equal bidirectional causality.

Result for multivariate conditional cCCM: In this part, we conducted multivariate KNN search based conditional cCCM and conditional cCCM for all the $10 \times 9 = 90$ unidirectional ROI pairs. In both cases, the conditional cCCM was calculated with respect to all the other ROIs and used the same interpolated fMRI sequences of length $n = 383$ as in the bivariate case.

The results corresponding to multivariate KNN search based conditional cCCM and CCM are shown in Supplementary Figure 10. As can be seen, both conditional cCCM and conditional CCM are very sensitive to the interdependence between the brain regions under consideration and the other regions in the state space. Due to rich brain network diversity, the multivariate conditional CCM and cCCM causality ratios with respect to the rest of the state space turn out to be very small or insignificant and cannot really be used for unidirectional causality detection.

We further checked the multivariate conditional CCM and cCCM causality with respect to individual regions. More specifically, we compared the conditional CCM and cCCM causality from $LV1 \to LPWM$, $LV1 \to RFWM$ and $LPPA \to LPWM$ with respect to other individual ROI regions. It can be observed that: (i) Conditional CCM and cCCM ratios with respect to individual regions are highly consistent; (ii) RV1 has the most significant impact on the conditional causality from $LV1 \to LPWM$ and $LV1 \to RFWM$. This implies that RV1 has the highest interdependence with LV1, followed by LPPA and RPPA. (iii) RPPA has the most significant impact on the conditional causality from $LPPA \to LPWM$, followed by LV1 and RV1.

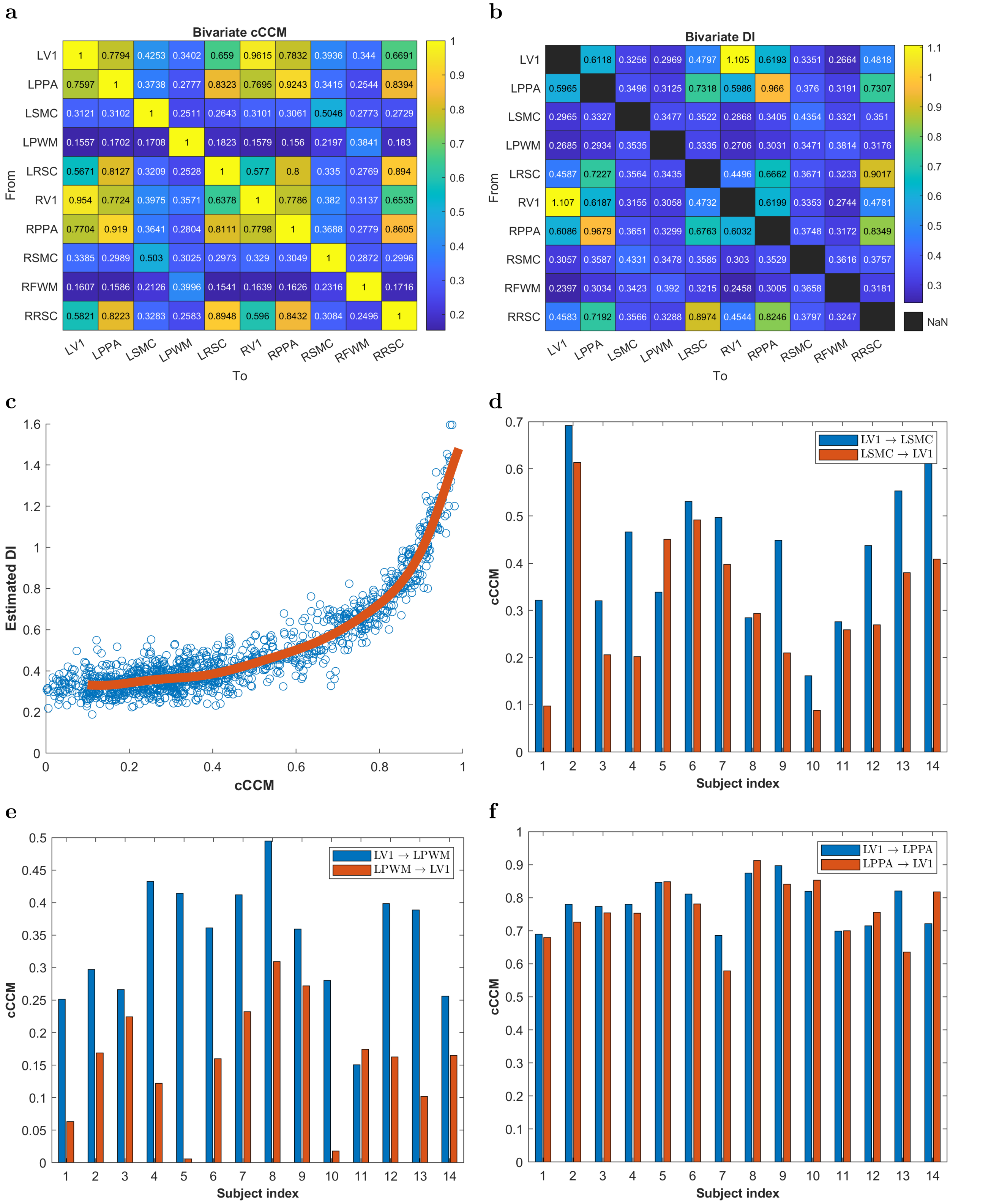

Supplementary Figure 8: **Bivariate cCCM and DI causality analysis based on visual task-fMRI.** (**a**) Heatmap of cCCM. (**b**) Heatmap of DI.(**C**) DI vs cCCM.(**d**) Unidirectional cCCM causality: LV1→LSMC. (**e**) Unidirectional cCCM causality: LV1→LPWM. (**f**) Bidirectional cCCM causality between LV1 and LPPA.

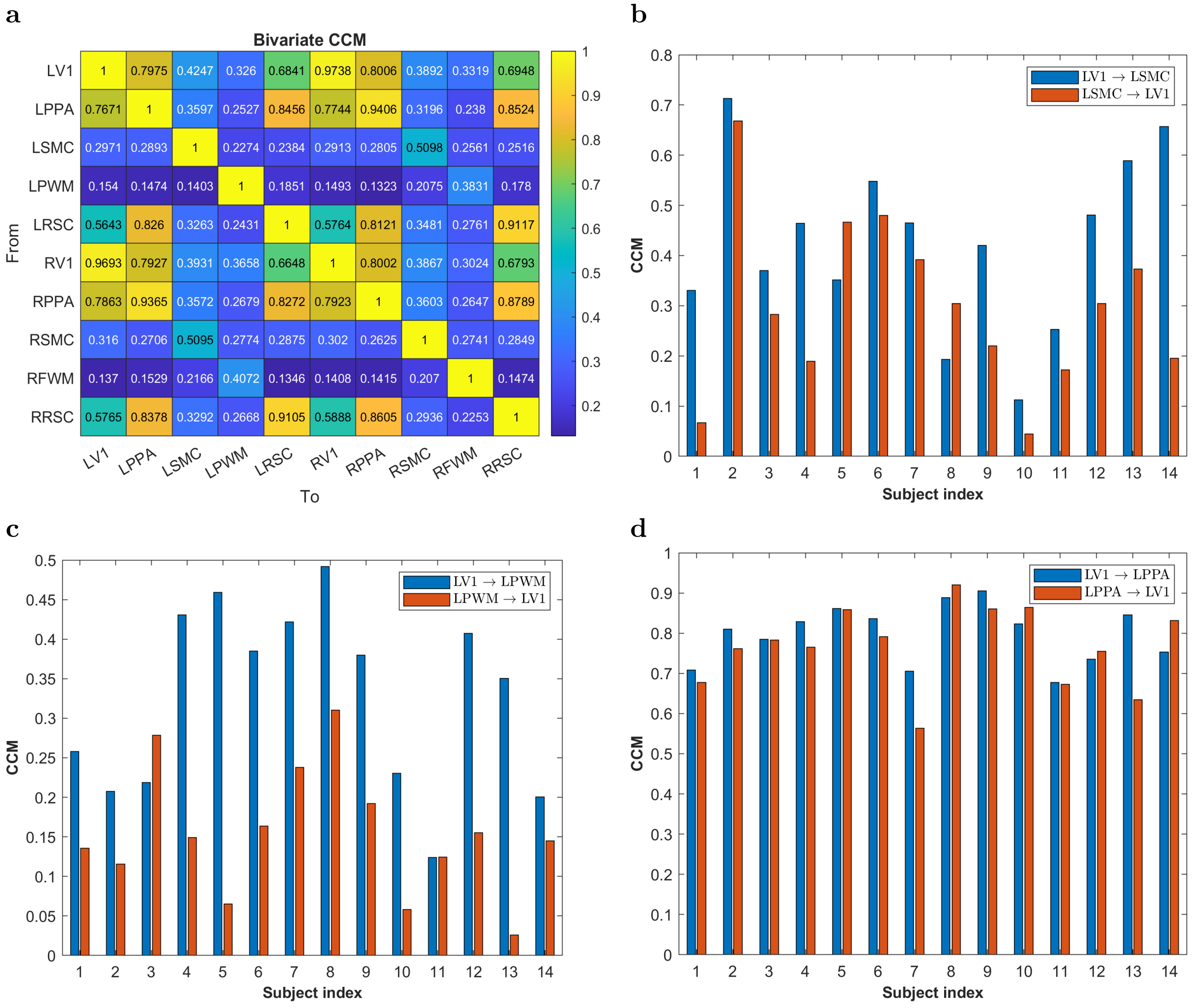

Supplementary Figure 9: **Bivariate CCM causality analysis based on visual task-fMRI.** (**a**) Heatmap of CCM. (**B**) Unidirectional CCM causality: LV1→LSMC. (**C**) Unidirectional CCM causality: LV1→LPWM. (**D**) Bidirectional CCM causality between LV1 and LPPA.

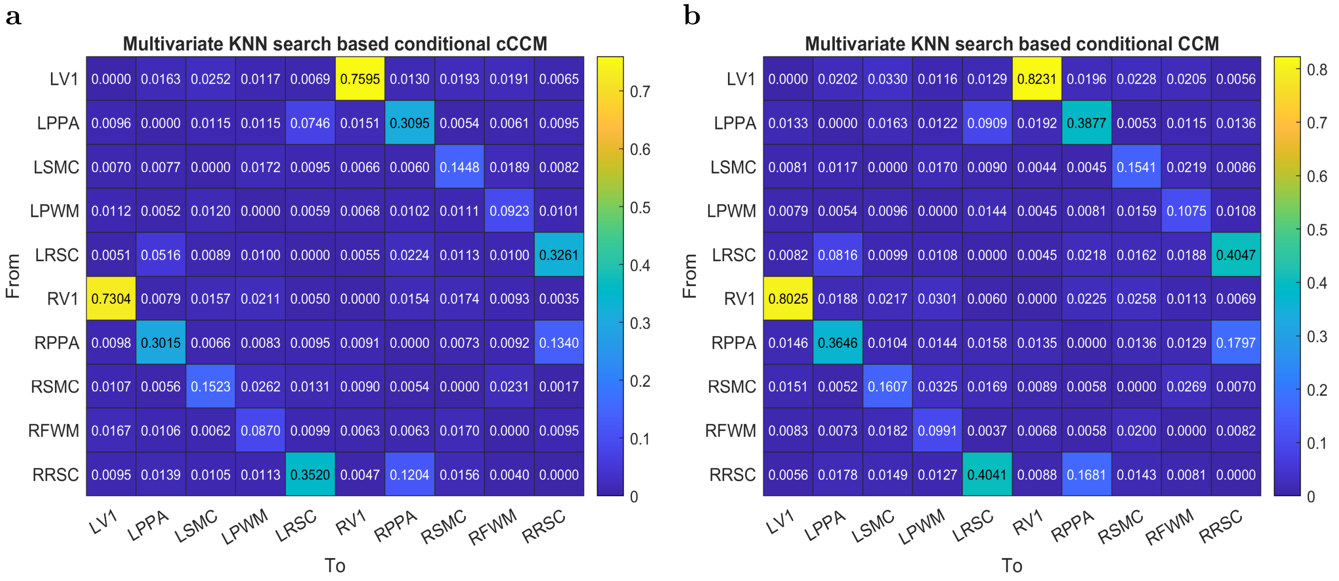

Supplementary Figure 10: **Multivariate conditional cCCM and CCM causality with respect to all the other ROI regions.** (**a**) Heatmap of conditional cCCM. (**b**) Heatmap of conditional CCM. As can be seen, due to rich brain network diversity, the multivariate conditional CCM and cCCM causality ratios with respect to the rest of the state space turn out to be very small or insignificant and cannot be used for unidirectional causality detection.

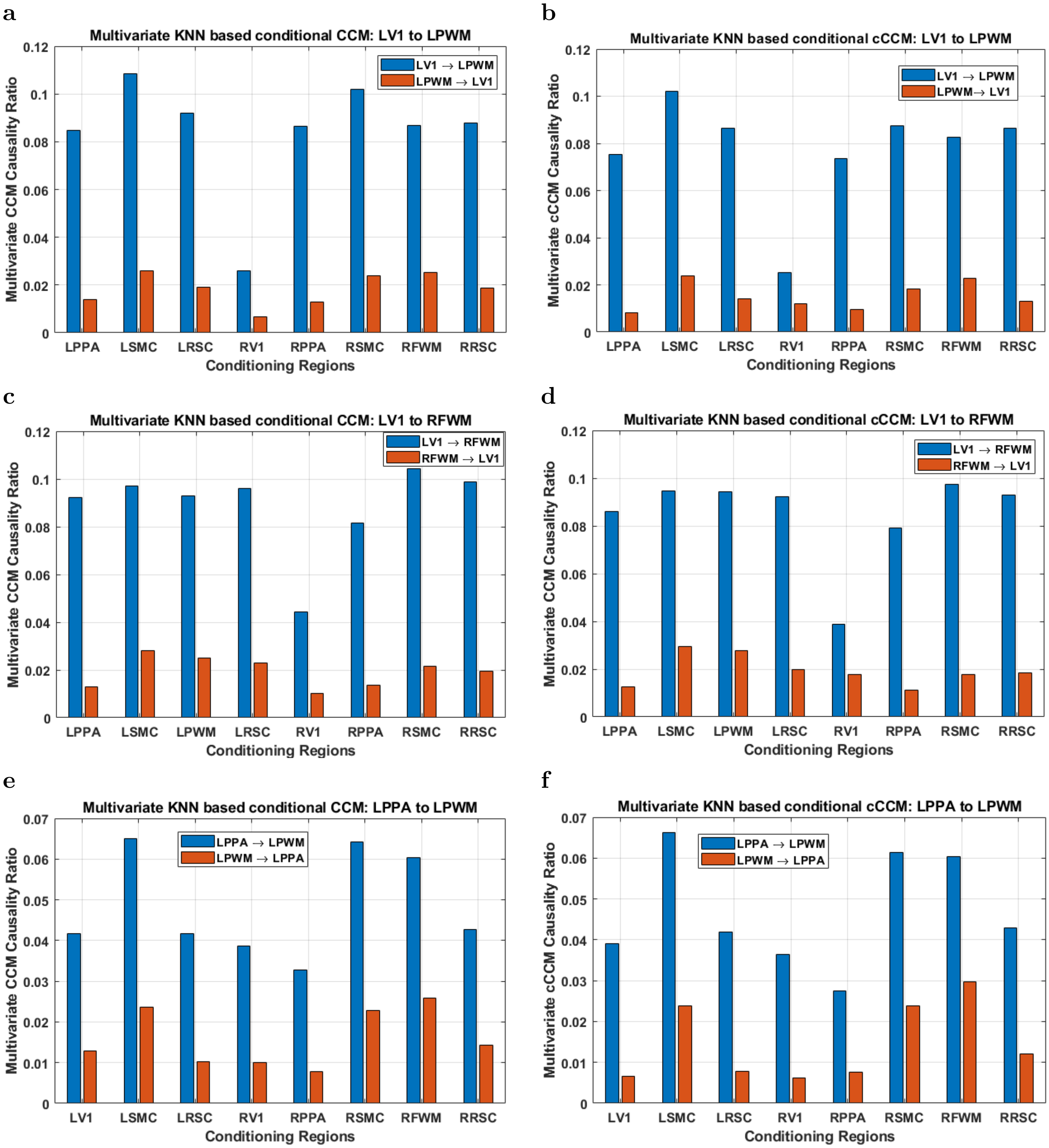

Supplementary Figure 11: **Multivariate conditional CCM and cCCM with respect to individual regions.** (a) Conditional CCM from $LV1 \rightarrow LPWM$, (b) Conditional cCCM from $LV1 \rightarrow LPWM$,(c) Conditional CCM from $LV1 \rightarrow RFWM$, (d) Conditional cCCM from $LV1 \rightarrow RFWM$, (e) Conditional CCM from $LPPA \rightarrow LPWM$, (f) Conditional cCCM from $LPPA \rightarrow LPWM$. The results indicated that: (i) Conditional CCM and cCCM ratios with respect to individual regions are highly consistent; (ii) RV1 has the most significant impact on the conditional causality from $LV1 \rightarrow LPWM$ and $LV1 \rightarrow RFWM$. This implies that RV1 has the highest interdependence with LV1, followed by LPPA and RPPA. (iii) RPPA has the most significant impact on the conditional causality from $LPPA \rightarrow LPWM$, followed by LV1 and RV1.

Supplementary Table 1: The ROI region pairs which show consistent unidirectional causality across bivariate cCCM, CCM, DI, and the majority of subjects. Here, *Diff* means the causation difference in the two opposite directions.

Descending order in bivariate cCCM	Region pairs	Diff. of bivariate cCCM	# of consistent subjects	Diff. of bivariate CCM	# of consistent subjects	Diff. of bivariate DI	# of consistent subjects	Diff. of Conditional CCM	# of consistent subjects	Diff. of Conditional cCCM	# of consistent subjects
1	RV1 to LPWM	0.1992	14	0.2166	14	0.0323	13	0.0256	12	0.0143	8
2	LV1 to LPWM	0.1845	13	0.1720	12	0.0307	13	0.0037	9	0.0005	7
3	LV1 to RFWM	0.1833	12	0.1949	13	0.0338	13	0.0122	10	0.0024	9
4	RV1 to RFWM	0.1498	11	0.1616	11	0.0326	12	0.0045	9	0.0030	8
5	RPPA to LPWM	0.1244	11	0.1357	13	0.0211	9	0.0063	7	-0.0018	10
6	RPPA to RFWM	0.1153	11	0.1232	11	0.0266	11	0.0071	7	0.0030	9
7	LV1 to LSMC	0.1132	12	0.1276	12	0.0310	11	0.0250	11	0.0182	10
8	LPPA to LPWM	0.1075	13	0.1054	12	0.0161	11	0.0067	7	0.0063	10

*Due to rich diversity in the brain network, the multivariate conditional CCM and cCCM causality ratios with respect to the rest of the state space turn out to be very small or insignificant and cannot be used for unidirectional causality detection.

ROI region pairs which show consistent unidirectional causality: The ROI region pairs which show consistent unidirectional causality across bivariate CCM, cCCM, DI, and across majority of the subjects for all these three models are shown in Supplementary Table 1. Here region pairs with averaged causation difference (between the two opposite directions) larger than 0.1 for both bivariate CCM and cCCM, and with average difference larger than 0.01 for DI were identified as ROI pairs with significant unidirectional causality, which include: RV1 $\rightarrow$ LPWM, LV1 $\rightarrow$ LPWM, LV1 $\rightarrow$ RFWM, RV1 $\rightarrow$ RFWM, RPPA $\rightarrow$ LPWM, RPPA $\rightarrow$ RFWM, LV1 $\rightarrow$ LSMC, and LPPA $\rightarrow$ LPWM.

For Multivariate conditional CCM and cCCM with respect to all the rest of the ROI regions, no apparent unidirectional causality observation can be recognized since the causality ratios are generally very small due to the rich diversity among different brain regions. However, as shown in Figure 11, multivariate conditional CCM and cCCM with respect to individual regions can detect unidirectional causality and demonstrate the impact of interdependence between the ROI regions on the conditional causality.

7 The Impact of Estimation Error on cCCM

In this section, we analyze the impact of estimation error or noise on cCCM. Without loss of generality, we consider $\rho_{\text{cCCM}}(X \rightarrow Y) = \rho(\mathbf{Y}^n, \hat{\mathbf{Y}}^n) \approx \rho(Y, \hat{Y})$ as an example. Recall that

$$\hat{Y}(t)|\mathbf{M}_x = \sum_{i=1}^{E+1} w_i Y(t_i) \tag{46}$$

where $t_i < t$, and

$$w_i = \frac{u_i}{\sum\limits_{j=1}^{E+1} u_j}, \quad with \quad u_i = exp\{-\frac{d(\bar{x}(t), \bar{x}(t_i))}{d(\bar{x}(t), \bar{x}(t_1))}\}.$$

Here $d(\cdot, \cdot)$ denotes the Euclidean distance between two vectors.

When estimation error presents, we can model $\hat{Y}$ as

$$\hat{Y} = Y + n_e \tag{47}$$

where n_e is the estimation error independent of Y. Without loss of generality, we assume that n_e is of zero-mean and has variance σ_e^2.

Recall that

$$\rho(Y, \hat{Y}) = \frac{E\{(Y - \mu_Y)(\hat{Y} - \mu_{\hat{Y}})\}}{\sigma_Y \sigma_{\hat{Y}}}, \tag{48}$$

and

$$\mu_{\hat{Y}} = E\{Y + n_e\} = \mu_Y, \tag{49}$$

$$\sigma_{\hat{Y}}^2 = \sigma_Y^2 + \sigma_e^2, \tag{50}$$

$$\begin{aligned} E\{(Y-\mu_Y)(\hat{Y}-\mu_{\hat{Y}})\} &= E\{(Y-\mu_Y)(Y+n_e-\mu_Y)\} \\ &= E\{(Y-\mu_Y)^2 + n_e(Y-\mu_Y)\} \\ &= E\{(Y-\mu_Y)^2\} \\ &= \sigma_Y^2 \end{aligned} \tag{51}$$

Therefore, we have

$$\rho(Y, \hat{Y}) = \frac{\sigma_Y}{\sqrt{\sigma_Y^2 + \sigma_e^2}}. \tag{52}$$

As can be seen, *when noise presents, the estimation error power* σ_e^2 *will increase, and hence* $\rho_{cCCM}(X \to Y) \approx \rho(Y, \hat{Y})$ *will decrease.*

8 Discussions on the Choice of the Shadow Manifold Dimension E and Signal Lag τ

Consider two dynamically coupled variables X and Y which share the same attractor manifold $\mathbf{M}$. Let $\mathbf{X}^n = [X_1, X_2, \cdots, X_n]$ and $\mathbf{Y}^n = [Y_1, Y_2, \cdots, Y_n]$ be the time series consisting of samples of X and Y, respectively. Recall that the shadow manifolds with respect to $\mathbf{X}^n$ and $\mathbf{Y}^n$ are defined as

$$\begin{aligned} \mathbf{M}_x &= \{\mathbf{x}_t \mid \mathbf{x}_t = [X_t, X_{t-\tau}, \cdots, X_{t-(E-1)\tau}],\ t = 1 + (E-1)\tau, \cdots, n\} \\ \mathbf{M}_y &= \{\mathbf{y}_t \mid \mathbf{y}_t = [Y_t, Y_{t-\tau}, \cdots, Y_{t-(E-1)\tau}],\ t = 1 + (E-1)\tau, \cdots, n\} \end{aligned}$$

In [2], Sugihara et al. pointed out that "in general, state space reconstruction (SSR) methods work best when the system is nonlinear and can be approximated in few dimensions and especially when observational noise is not excessive", and also that the amount of data required depends on the attractor dimensionality. In the implementation of the cross-mapping technique, the choice of the dimension of the shadow manifolds E plays a critical role. According to Takens' theorem [3] and Whitney's embedding theorem [20, 21], the magic number is $E = 2d + 1$, and often less [2], where $d \leq E$ is the dimension of the attractor $\mathbf{M}$ shared by X and Y. Another parameter that determines the definition of the shadow manifolds is the signal lag τ. Here we illustrate the impact of E and τ on the performance of cCCM through simulation examples.

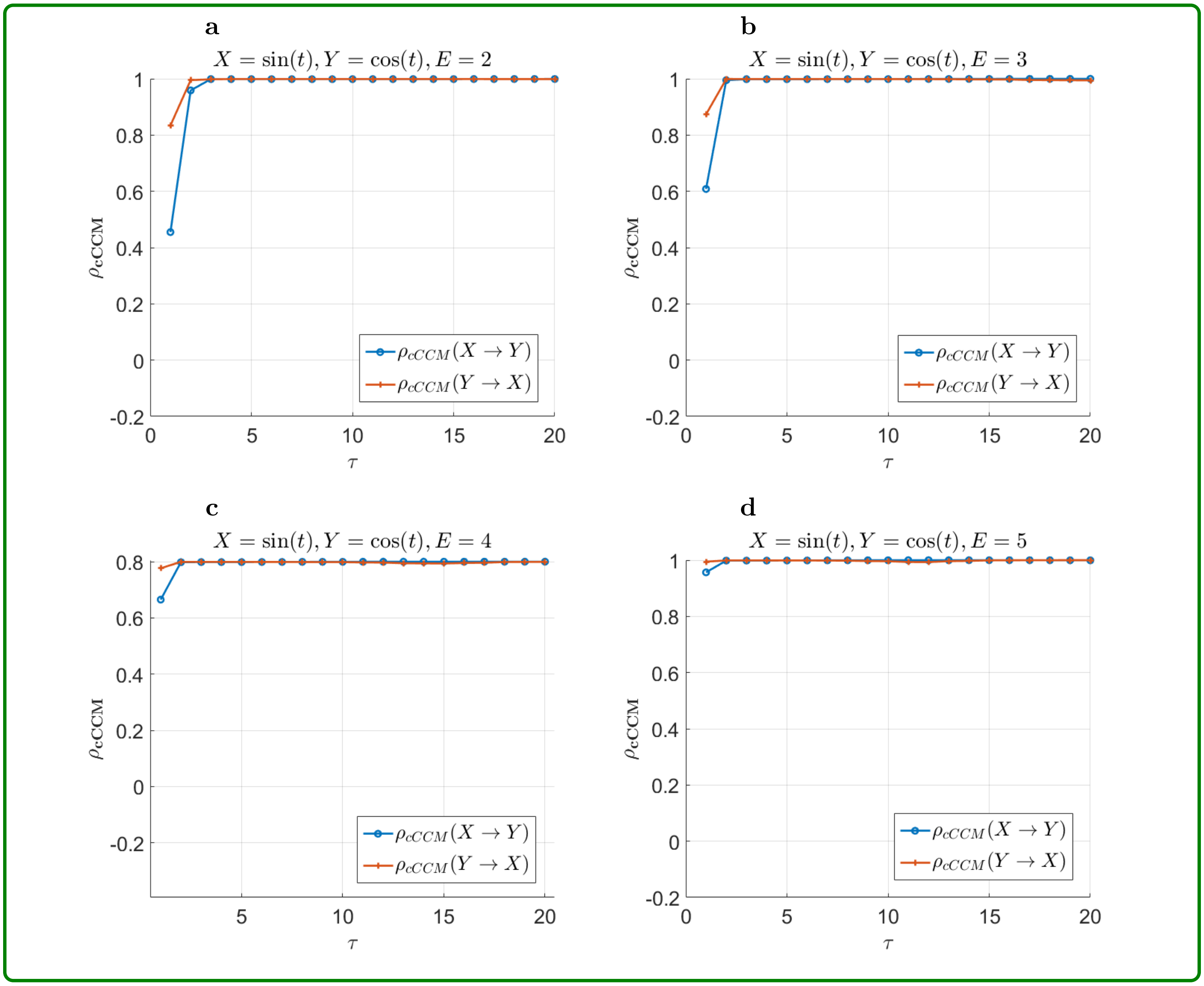

Supplementary Figure 12: **The impact of E and τ on cCCM:** $X = sin(t)$ and $Y = cos(t)$ with $t = 0 : 0.01\pi : 2\pi$.

The performance cCCM is relatively stable with respect to E and τ when $d \le E \le 2d+1$. In our simulation, the time series corresponding to X and Y can be represented as a $2 \times L$ matrix. We take $d \le 2$, since d denotes the Minkowski dimension or box-counting dimension of the attractor $\mathbf{M}$ in fractal geometry and could be a fraction which is less than 2. Following Takens' theorem and Whitney's embedding theorem, we choose $2 \le E \le 5$, and allow τ to change over a wide range $1 \le \tau \le 20$.

The **first example** considered is $X = \sin(t)$ and $Y = \cos(t)$ where $t = 0 : 0.01\pi : 2\pi$, and the result is shown in Supplementary Figure 12. In this case, a strong bidirectional causality exists between X and Y. From the simulation result, we can see that: (i) when $E = 2$, the expected causal relationship could be observed when $\tau \ge 3$; (ii) When $E \ge 3$, we only need $\tau \ge 2$ to detect the expected bidirectional causality. The product of E and τ determines the data span in each vector of $\mathbf{M}_x$, and the data span needs to be large enough for satisfying results.

The **second example** considered is X =randn(1024, 1), $Y = X^2$, and the result is shown in Supplementary Figure 13. In this example, for $2 \le E < 5$, τ does not show significant impact on cCCM, largely because that X is a random process consisting of independent and identically distributed (i.i.d.) Gaussian random variables. Again, the performance of cCCM is relatively stable with respect to E and τ when $d \le E \le 2d+1$.

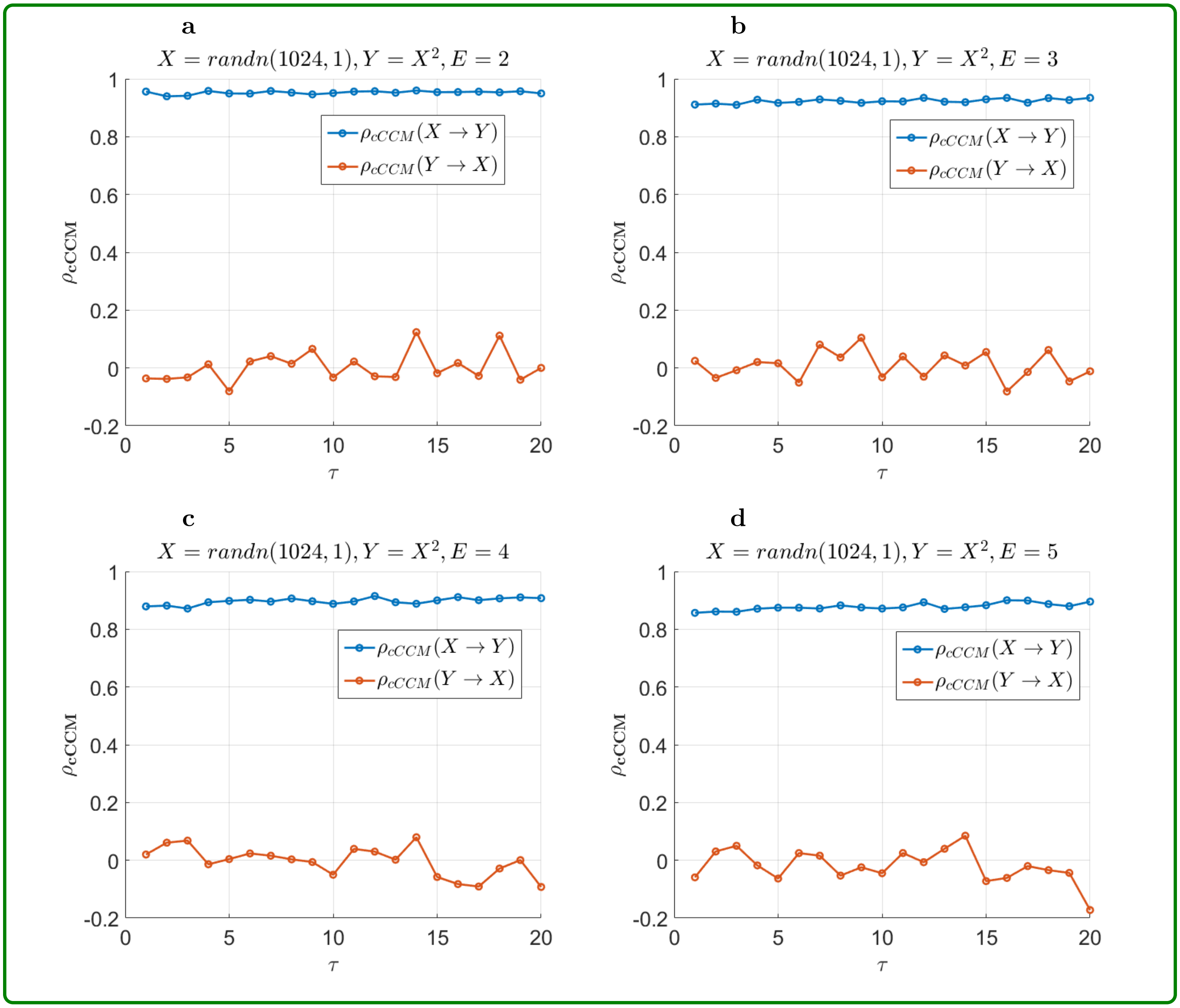

Supplementary Figure 13: **The impact of E and τ on cCCM:** X =randn(1024, 1) and $Y = X^2$.

Parameters used in our simulation and experimental examples Based on our exploration above, in the article, we generally chose $E = 5$ and $\tau = 1$ in the simulation examples and the causality analysis of the experimental fMRI data, except for *Example 14* in the manuscript, where we investigated both $E = 5, \tau = 1$ and $E = 5, \tau = 5$. This is because that in Example 14, the sampling period is very small (as the sampling frequency is much higher than the Nyquist rate), and we need a larger τ to get better noise immunity.

The simulation results for $E = 5, \tau = 1$ and $E = 5, \tau = 5$ are shown in Supplementary Tables 2 and 3. As can be seen, $E = 5$ and $\tau = 5$ can achieve much better noise immunity since the data span, $E \cdot \tau$, is sufficiently long.

Example 14	Direction	0 dB	5 dB	10 dB	15 dB	20 dB	Noise Free
14. $X(t) = \sin(t) + n_1$,	$X \to Y$	0.1623	0.2540	0.3202	0.3956	0.5262	0.9566
$Y(t) = \cos(t) + n_2$.	$Y \to X$	0.3104	0.4821	0.5761	0.6668	0.7792	0.9945
$t = 0 : 0.01\pi : 2\pi$	Difference	-0.1481	-0.2281	-0.2559	-0.2712	-0.2530	-0.0379

Supplementary Table 2: Performance of cCCM under additive white Guassian noise: $E = 5$ and $\tau = 1$.

Example 14	Direction	0 dB	5 dB	10 dB	15 dB	20 dB	Noise Free
14. $X(t) = \sin(t) + n_1$,	$X \to Y$	0.2327	0.5062	0.7882	0.9403	0.9798	0.9985
$Y(t) = \cos(t) + n_2$.	$Y \to X$	0.4402	0.6827	0.8632	0.9607	0.9877	0.9991
$t = 0 : 0.01\pi : 2\pi$	Difference	-0.2074	-0.1765	-0.0750	-0.0204	-0.0079	-0.0006

Supplementary Table 3: Performance of cCCM under additive white Gaussian noise: $E = 5$ and $\tau = 5$.

If E is too large, cCCM may no longer deliver meaningful results. The following example shows that if E is much larger than $2d + 1$, cCCM may deliver inaccurate or even incorrect results. Consider X =randn(1024, 1) and $Y = X^2$, where there is a strong unidirectional causality from X to Y, and no causation in the inverse direction. Choose $\tau = 1$. From Supplementary Figure 14, we can see that as E increases, cCCM value keeps on decreasing and is already below 0.2 when $E = 60$, and can no longer reflect the strong unidirectional causality from X to Y.

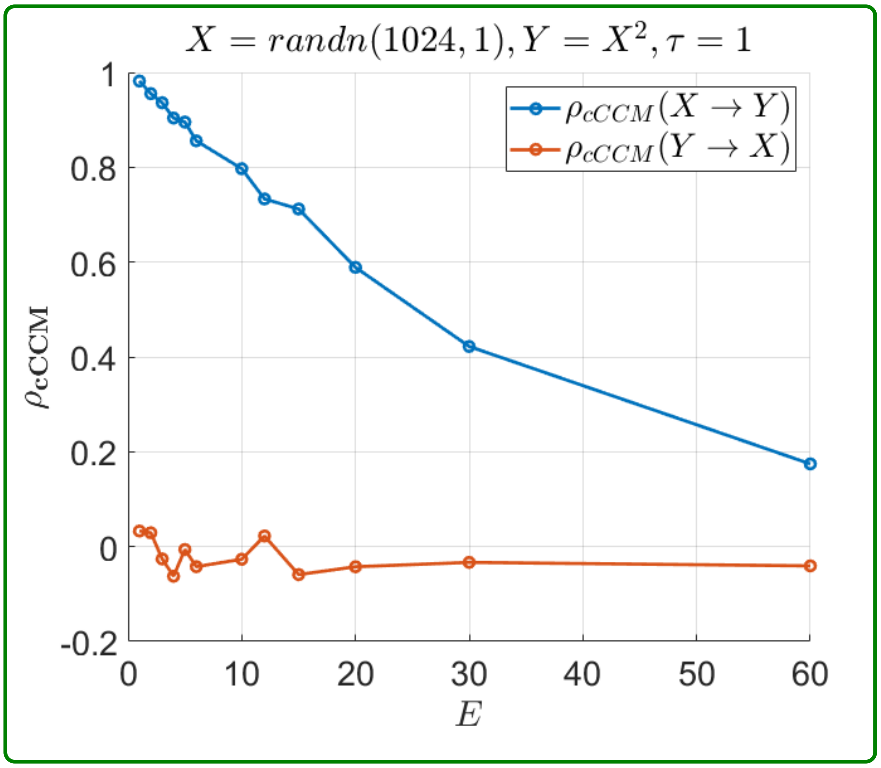

Supplementary Figure 14: **Large** E **may downgrade the performance of cCCM:** $X = randn(1024, 1)$, $Y = X^2$ and $\tau = 1$.

Choices of E and τ **for systems with memory** We examined cCCM causation in systems with memory and found that: the dominant delays (i.e., delays corresponding to the dominant peaks) in the channel impulse response do impose requirements on both the dimension of the shadow manifolds E and the signal lag τ used in shadow manifold construction. More specifically, for the causality to be accurately detected in systems with memory, we need: (i) The product $E \cdot \tau$ is larger than all the dominant delays (i.e., delays corresponding to the dominant peaks in the channel impulse response), and (ii) For each time instant t, all the samples with dominant delays appear in the constructing vector $\mathbf{x}(t) = [X(t), X(t - \tau), \cdots, X(t - (E - 1)\tau)]$ of the shadow manifold.

This was illustrated through simulation examples in Supplementary Table 4. From Example 8-1, $E = 3, \tau = 1$, we can see that if *Condition (i)* is not satisfied, then the corresponding causality cannot be detected. If *Condition (i)* is satisfied but *Condition (ii)* is not fully satisfied, then only the causality corresponding to the dominant delays that appear in the constructing vector of the shadow manifold will

be detected. This can be seen from Example 8-2, $E = 3, \tau = 2$, where $Y(t) = 0.8X(t-1) + 0.8X(t-4)$, $\mathbf{x}(t) = [X(t), X(t-2), \cdots, X(t-4)]$, and only the causality corresponding to the item $0.8X(t-4)$ can be detected and the causality corresponding to the item $0.8X(t-1)$ cannot be detected, because $X(t-4)$ appears in $\mathbf{x}(t)$ but $X(t-1)$ does not. In Example 8-3, $E = 3, \tau = 2$, where $Y(t) = 0.8X(t-2)+0.8X(t-4)$, $\mathbf{x}(t) = [X(t), X(t-2), \cdots, X(t-4)]$, the causality can be accurately detected since both $X(t-2)$ and $X(t-4)$ appear in $\mathbf{x}(t)$.

From Table 4 and Figures 15 and 16, it can be seen, cCCM and CCM causations are highly consistent, and they also share similar convergence speeds and deliver comparable MSE in state space reconstruction based on geometric cross mapping.

Supplementary Table 4: cCCM versus CCM: simulation examples on systems with memory

Simulation Examples		**cCCM**	**CCM**
Example 8-1.<br>X =randn(1, 1024)<br>$Y(t) = 0.1X(t-1) + 0.8X(t-4)$	$E = 3, \tau = 1$	$\rho_{\text{cCCM}}(X \to Y) : 0.0173$<br>$\rho_{\text{cCCM}}(Y \to X) : 0.0412$<br>$\text{MSE}(\mathbf{X}^n, \hat{\mathbf{X}}^n) : 1.2821$<br>$\text{MSE}(\mathbf{Y}^n, \hat{\mathbf{Y}}^n) : 0.8557$	$\rho_{\text{CCM}}(X \to Y) : 0.0100$<br>$\rho_{\text{CCM}}(Y \to X) : 0.0498$<br>$\text{MSE}(\mathbf{X}^n, \hat{\mathbf{X}}^n) : 1.2505$<br>$\text{MSE}(\mathbf{Y}^n, \hat{\mathbf{Y}}^n) : 0.8526$
	$E = 3, \tau = 2$	$\rho_{\text{cCCM}}(X \to Y) : 0.9714$<br>$\rho_{\text{cCCM}}(Y \to X) : 0.0298$<br>$\text{MSE}(\mathbf{X}^n, \hat{\mathbf{X}}^n) : 1.2633$<br>$\text{MSE}(\mathbf{Y}^n, \hat{\mathbf{Y}}^n) : 0.0393$	$\rho_{\text{CCM}}(X \to Y) : 0.9826$<br>$\rho_{\text{CCM}}(Y \to X) : 0.0487$<br>$\text{MSE}(\mathbf{X}^n, \hat{\mathbf{X}}^n) : 1.2389$<br>$\text{MSE}(\mathbf{Y}^n, \hat{\mathbf{Y}}^n) : 0.0236$
	$E = 5, \tau = 1$	$\rho_{\text{cCCM}}(X \to Y) : 0.9561$<br>$\rho_{\text{cCCM}}(Y \to X) : -0.0295$<br>$\text{MSE}(\mathbf{X}^n, \hat{\mathbf{X}}^n) : 1.1714$<br>$\text{MSE}(\mathbf{Y}^n, \hat{\mathbf{Y}}^n) : 0.0733$	$\rho_{\text{CCM}}(X \to Y) : 0.9777$<br>$\rho_{\text{CCM}}(Y \to X) : -0.0633$<br>$\text{MSE}(\mathbf{X}^n, \hat{\mathbf{X}}^n) : 1.2009$<br>$\text{MSE}(\mathbf{Y}^n, \hat{\mathbf{Y}}^n) : 0.0429$
Example 8-2.<br>X =randn(1, 1024)<br>$Y(t) = 0.8X(t-1) + 0.8X(t-4)$	$E = 3, \tau = 1$	$\rho_{\text{cCCM}}(X \to Y) : 0.5620$<br>$\rho_{\text{cCCM}}(Y \to X) : 0.0114$<br>$\text{MSE}(\mathbf{X}^n, \hat{\mathbf{X}}^n) : 1.3030$<br>$\text{MSE}(\mathbf{Y}^n, \hat{\mathbf{Y}}^n) : 0.8850$	$\rho_{\text{CCM}}(X \to Y) : 0.5819$<br>$\rho_{\text{CCM}}(Y \to X) : 0.0086$<br>$\text{MSE}(\mathbf{X}^n, \hat{\mathbf{X}}^n) : 1.2857$<br>$\text{MSE}(\mathbf{Y}^n, \hat{\mathbf{Y}}^n) : 0.8614$
	$E = 3, \tau = 2$	$\rho_{\text{cCCM}}(X \to Y) : 0.5607$<br>$\rho_{\text{cCCM}}(Y \to X) : 0.0038$<br>$\text{MSE}(\mathbf{X}^n, \hat{\mathbf{X}}^n) : 1.2796$<br>$\text{MSE}(\mathbf{Y}^n, \hat{\mathbf{Y}}^n) : 0.8615$	$\rho_{\text{CCM}}(X \to Y) : 0.5902$<br>$\rho_{\text{CCM}}(Y \to X) : 0.0167$<br>$\text{MSE}(\mathbf{X}^n, \hat{\mathbf{X}}^n) : 1.2652$<br>$\text{MSE}(\mathbf{Y}^n, \hat{\mathbf{Y}}^n) : 0.8416$
	$E = 5, \tau = 1$	$\rho_{\text{cCCM}}(X \to Y) : 0.9553$<br>$\rho_{\text{cCCM}}(Y \to X) : -0.0036$<br>$\text{MSE}(\mathbf{X}^n, \hat{\mathbf{X}}^n) : 1.1721$<br>$\text{MSE}(\mathbf{Y}^n, \hat{\mathbf{Y}}^n) : 0.1486$	$\rho_{\text{CCM}}(X \to Y) : 0.9744$<br>$\rho_{\text{CCM}}(Y \to X) : -0.0318$<br>$\text{MSE}(\mathbf{X}^n, \hat{\mathbf{X}}^n) : 1.1902$<br>$\text{MSE}(\mathbf{Y}^n, \hat{\mathbf{Y}}^n) : 0.0958$
Example 8-3.<br>X =randn(1, 1024)<br>$Y(t) = 0.8X(t-2) + 0.8X(t-4)$	$E = 3, \tau = 1$	$\rho_{\text{cCCM}}(X \to Y) : 0.5951$<br>$\rho_{\text{cCCM}}(Y \to X) : 0.0087$<br>$\text{MSE}(\mathbf{X}^n, \hat{\mathbf{X}}^n) : 1.2979$<br>$\text{MSE}(\mathbf{Y}^n, \hat{\mathbf{Y}}^n) : 0.8963$	$\rho_{\text{CCM}}(X \to Y) : 0.6058$<br>$\rho_{\text{CCM}}(Y \to X) : -0.0030$<br>$\text{MSE}(\mathbf{X}^n, \hat{\mathbf{X}}^n) : 1.2773$<br>$\text{MSE}(\mathbf{Y}^n, \hat{\mathbf{Y}}^n) : 0.8785$
	$E = 3, \tau = 2$	$\rho_{\text{cCCM}}(X \to Y) : 0.9807$<br>$\rho_{\text{cCCM}}(Y \to X) : -0.0012$<br>$\text{MSE}(\mathbf{X}^n, \hat{\mathbf{X}}^n) : 1.2826$<br>$\text{MSE}(\mathbf{Y}^n, \hat{\mathbf{Y}}^n) : 0.0584$	$\rho_{\text{CCM}}(X \to Y) : 0.9921$<br>$\rho_{\text{CCM}}(Y \to X) : 0.0547$<br>$\text{MSE}(\mathbf{X}^n, \hat{\mathbf{X}}^n) : 1.2142$<br>$\text{MSE}(\mathbf{Y}^n, \hat{\mathbf{Y}}^n) : 0.0253$
	$E = 5, \tau = 1$	$\rho_{\text{cCCM}}(X \to Y) : 0.9574$<br>$\rho_{\text{cCCM}}(Y \to X) : -0.0434$<br>$\text{MSE}(\mathbf{X}^n, \hat{\mathbf{X}}^n) : 1.1825$<br>$\text{MSE}(\mathbf{Y}^n, \hat{\mathbf{Y}}^n) : 0.1442$	$\rho_{\text{CCM}}(X \to Y) : 0.9774$<br>$\rho_{\text{CCM}}(Y \to X) : -0.0300$<br>$\text{MSE}(\mathbf{X}^n, \hat{\mathbf{X}}^n) : 1.1778$<br>$\text{MSE}(\mathbf{Y}^n, \hat{\mathbf{Y}}^n) : 0.0875$

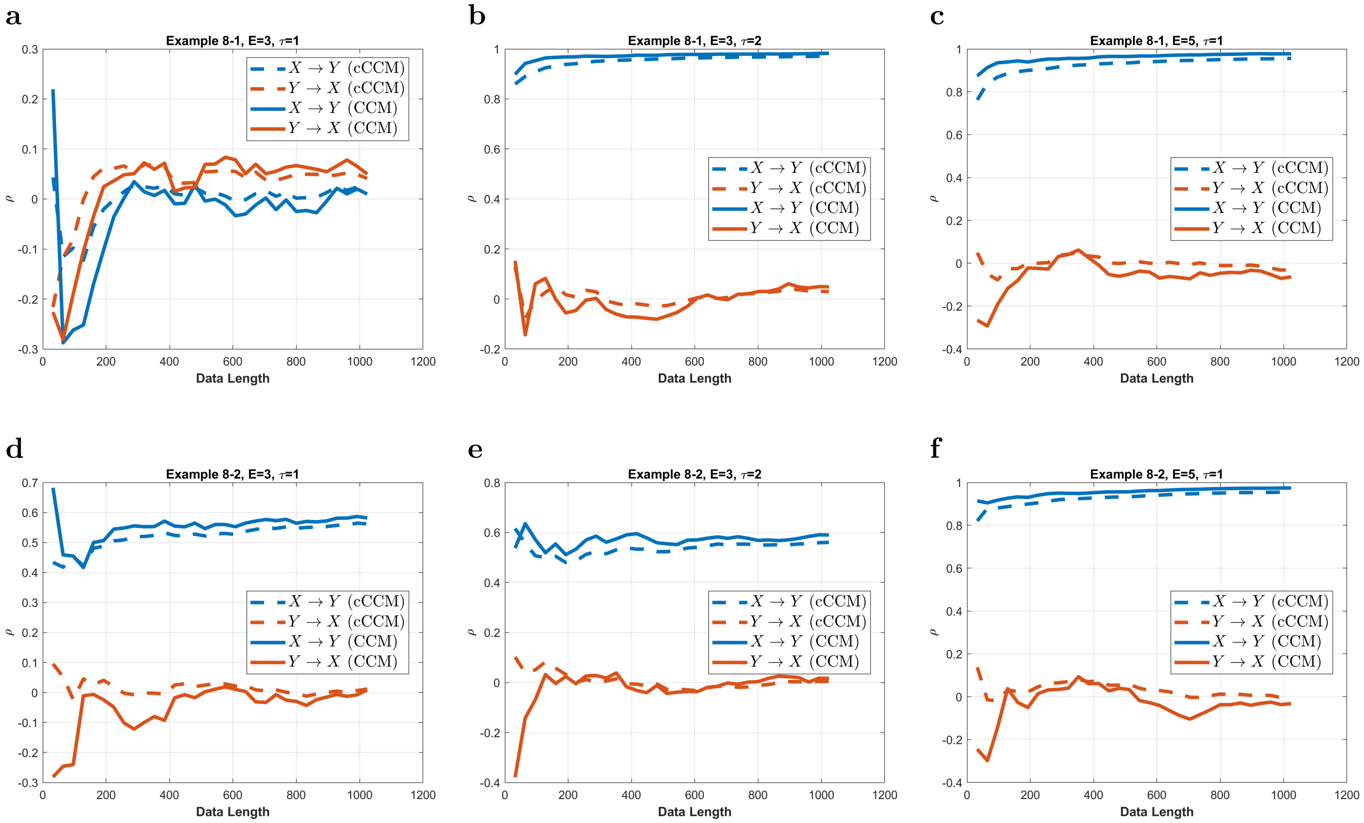

Supplementary Figure 15: cCCM versus CCM – causation values: (a) Example 8-1, $E = 3, \tau = 1$. (b) Example 8-1, $E = 3, \tau = 2$. (c) Example 8-1, $E = 5, \tau = 1$. (d) Example 8-2, $E = 3, \tau = 1$. (e) Example 8-2, $E = 3, \tau = 2$. (f) Example 8-2, $E = 5, \tau = 1$.

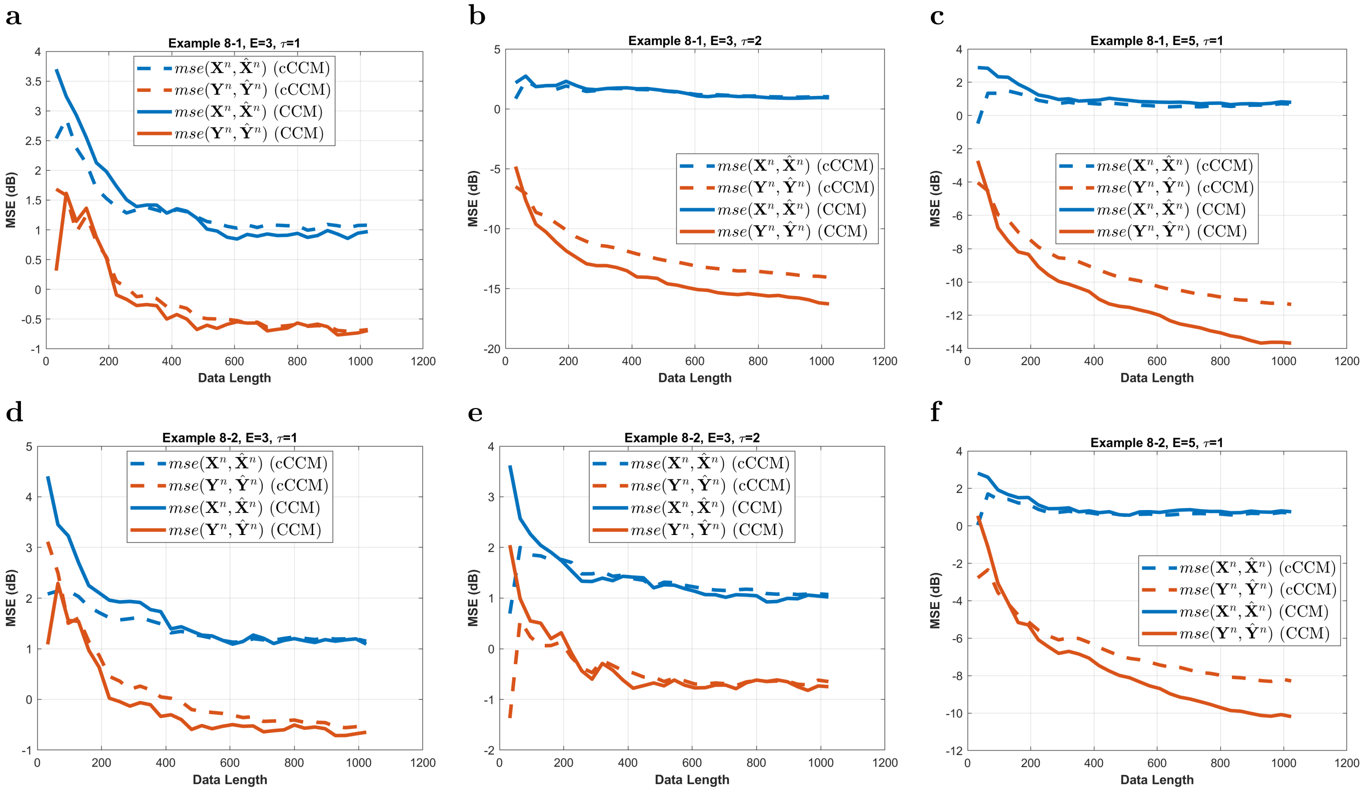

Supplementary Figure 16: cCCM versus CCM – MSE between the cross mapping based prediction and the true value: (a) Example 8-1, $E = 3, \tau = 1$. (b) Example 8-1, $E = 3, \tau = 2$. (c) Example 8-1, $E = 5, \tau = 1$. (d) Example 8-2, $E = 3, \tau = 1$. (e) Example 8-2, $E = 3, \tau = 2$. (f) Example 8-2, $E = 5, \tau = 1$.

9 How Many Monte Carlo Runs Do We Need to Evaluate the Noise Effect in cCCM and DI ?

To examine whether the number of Monte Carlo runs performed is sufficient in the Examples in Box 3 of the article (where all the results were averaged over 100 Monte Carlo runs), we evaluated the averaged cCCM and DI values (under different noise levels) versus the number of Monte Carlo runs. As can be seen from the figures below, both the averaged cCCM and DI values converge as the number of runs gets larger. Our analysis shows that 100 Monte Carlo runs are sufficient in these examples.

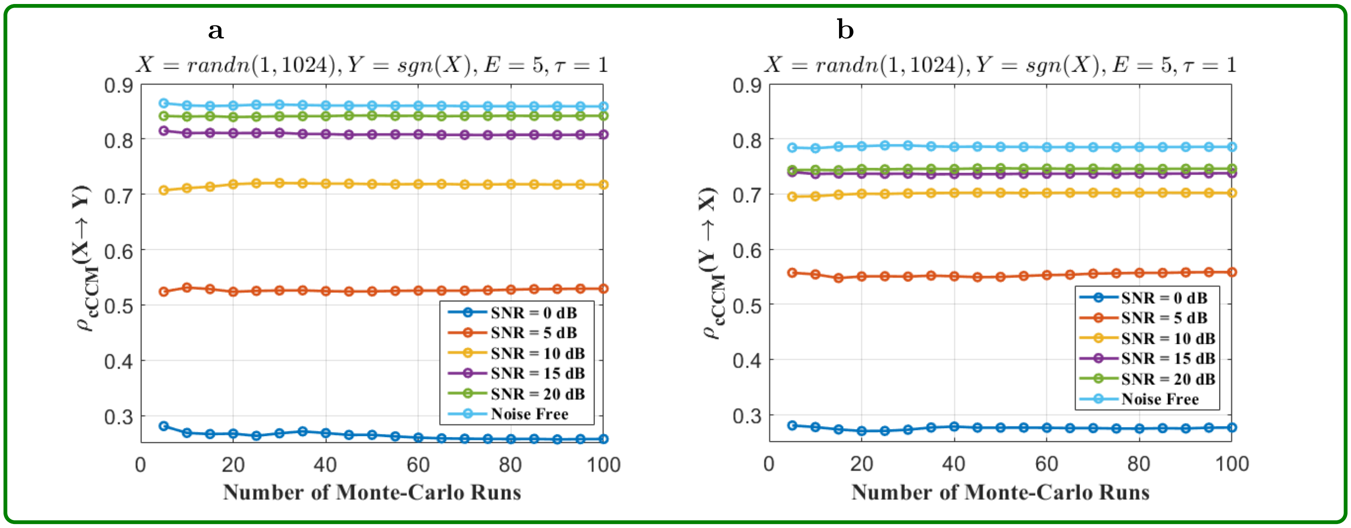

Supplementary Figure 17: **Averaged ρ_{cCCM} versus the number of Monte Carlo runs under different SNR levels:** $X = randn(1, 1024)$ and $Y = sgn(X)$.

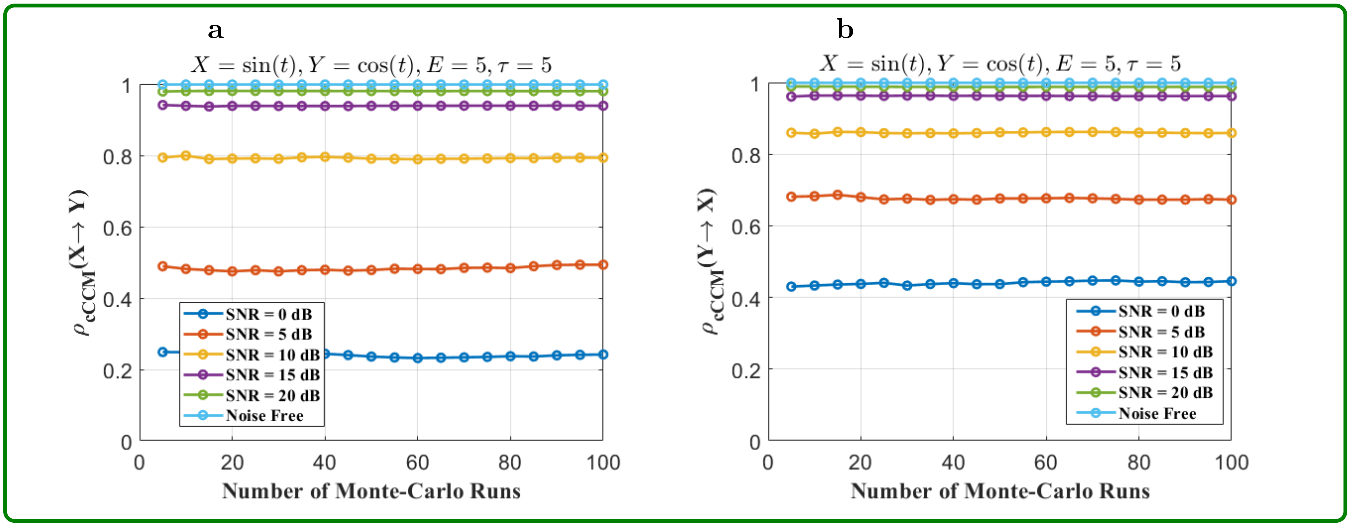

Supplementary Figure 18: **Averaged ρ_{cCCM} versus the number of Monte Carlo runs under different SNR levels:** $X = \sin(t)$, $Y = \cos(t)$, and $t = 0 : 0.01\pi : 2\pi$.

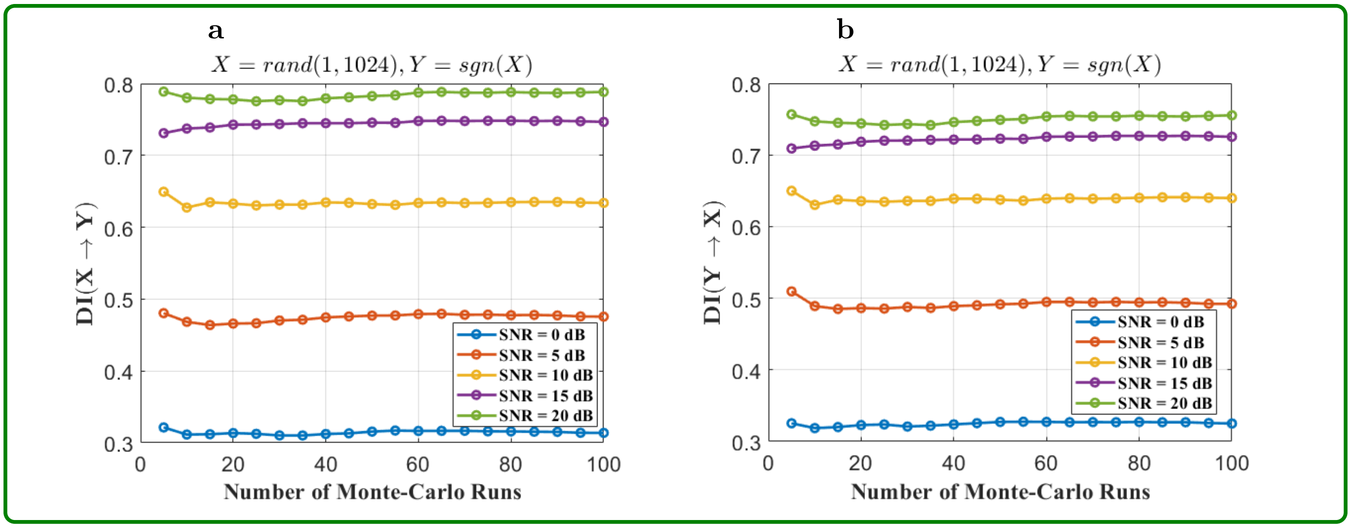

Supplementary Figure 19: **Averaged DI versus the number of Monte Carlo runs under different SNR levels:** $X = randn(1, 1024)$ and $Y = sgn(X)$.

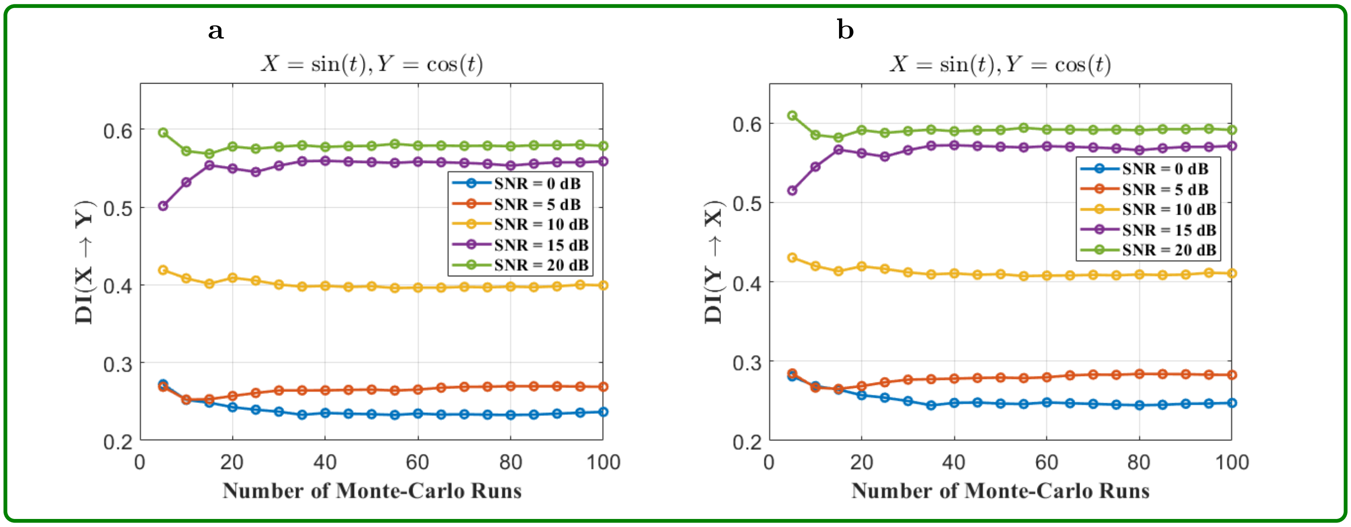

Supplementary Figure 20: **Averaged DI versus the number of Monte Carlo runs under different SNR levels:** $X = sin(t)$, $Y = cos(t)$, and $t = 0 : 0.01\pi : 2\pi$.

10 An Example on the Impact of Sampling Frequency on cCCM

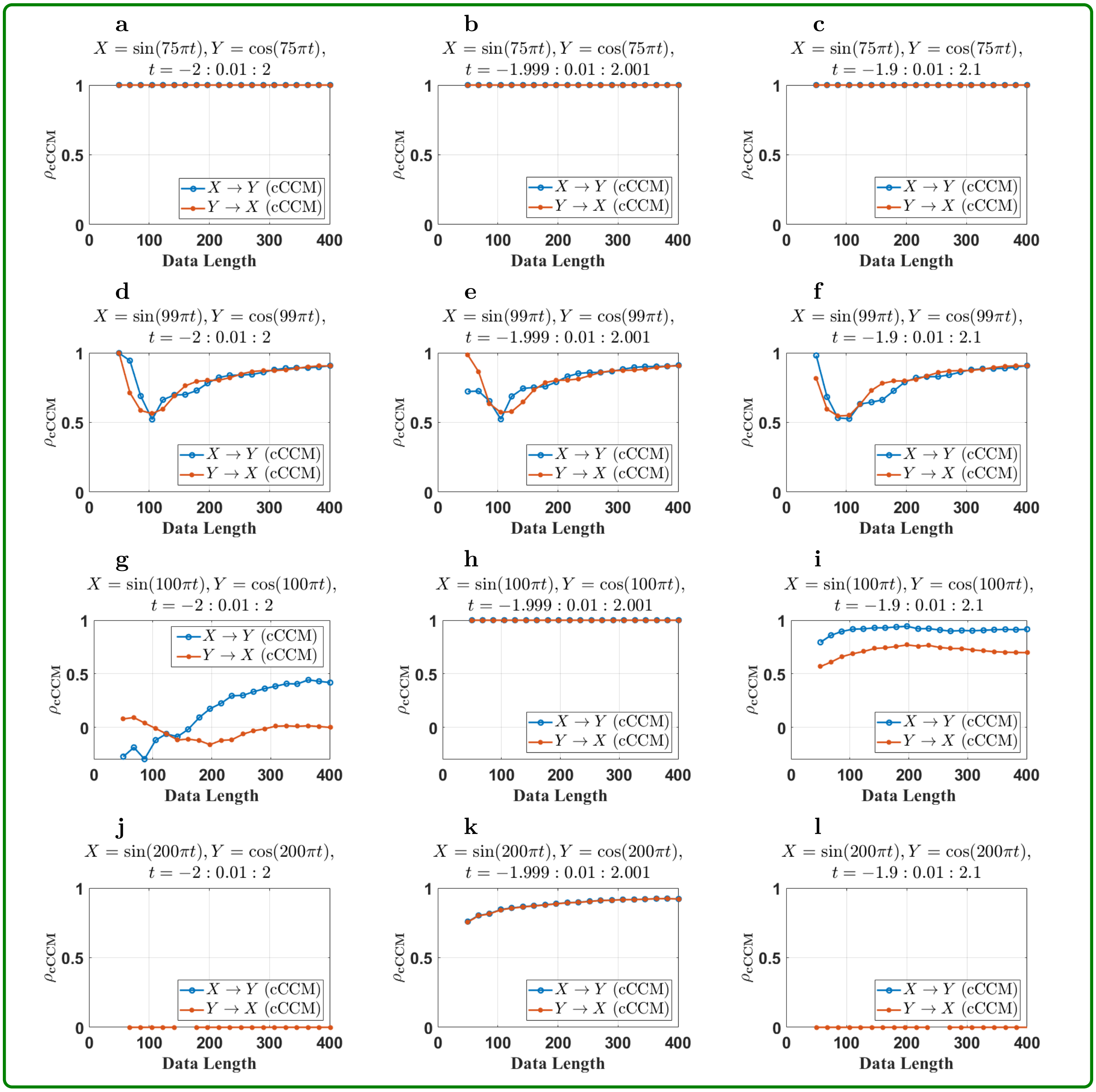

Supplementary Figure 21: **Impact of sampling frequency on cCCM convergence speed:** an illustration using sinusoidal waveforms with different frequencies. (a)-(c), f_0=37.5 Hz; (d)-(f), f_0=49.5Hz; (g)-(i), f_0=50Hz; (j)-(l), f_0=100Hz, where the sampling instant sequence was chosen as $t = [-2 : 0.01 : 2]$, $t = [-1.999 : 0.01 : 2.001]$, $t = [-1.9 : 0.01 : 2.1]$, respectively. Here f_0 denotes the frequency of the sinusoidal waveform, and the sampling frequency f_s = 100Hz for all the functions. As can be seen, cCCM works well when the sampling rate is well above the Nyquist rate and may require larger data length to converge as the sampling frequency is above but very close to the Nyquist sampling rate, as shown in figures (a)-(f), but may or may not deliver meaningful results when the sampling frequency is below or equal to the Nyquist rate, as shown in figures (g)-(l).

11 An Example on Data Repetition in Causality Analysis

The following example illustrates that even if X and Y are two independent signals which are not causally coupled, a causal pattern is enforced in the concatenated time series through data repetition.

Let $X = \text{randn}(1024, 1)$ and $Y = \text{randn}(1024, 1)$ be two independent normally distributed time series as in Example 2 of the article. We know that they are not causally coupled. Consider

$$X_1 = [X; X] \qquad Y_1 = [Y; Y],$$

then we have

$$\rho_{\text{cCCM}}(X_1 \to Y_1) = 0.6499, \qquad \rho_{\text{cCCM}}(Y_1 \to X_1) = 0.6516.$$

As can be seen, data concatenation creates new causality that does not exist in the original X and Y.

References

[1] Risk Reduction for Alzheimer's Disease (rrAD) (2016-2022), ClinicalTrials.gov identifier (NCT number): NCT02913664, https://clinicaltrials.gov/ct2/show/NCT02913664

[2] Sugihara, G., May, R., Ye, H., Hsieh, C. H., Deyle, E., Fogarty, M., & Munch, S. (2012). Detecting causality in complex ecosystems. Science (New York, N.Y.), 338(6106), 496–500. https://doi.org/10.1126/science.1227079

[3] Takens F. (1981). Detecting strange attractors in turbulence. In D. A. Rand and L.-S. Young (ed.). Dynamical Systems and Turbulence, Lecture Notes in Mathematics, vol. 898. SpringerVerlag. pp. 366–381.

[4] Yeung, R. W. (2002) A First Course in Information Theory: Information Technology: Transmission, Processing and Storage, 1st Edition, Springer.

[5] Granger, C. W. J. (1969). Investigating Causal Relations by Econometric Models and Cross-spectral Methods. Econometrica, 37(3), 424. https://doi.org/10.2307/1912791

[6] Granger, C. W. J. & Newbold, P. (1977). Forecasting Economic Time Series. New York: Academic Press. p. 225. ISBN 0122951506.

[7] Massey, J. (1990) Causality, feedback, and directed information, in The International Symposium on Information Theory and Its Applications, Honolulu, HI, Nov. 1990, pp. 303–305. CiteSeerX 10.1.1.36.5688.

[8] Schreiber, T. (2000). Measuring information transfer. Physical Review Letters. 85 (2): 461–464. doi:10.1103/PhysRevLett.85.461. PMID 10991308.

[9] Friston, K., Harrison, L., & Penny, W. (2003). Dynamic causal modelling. NeuroImage, 19(4), 1273–1302. https://doi.org/10.1016/s1053-8119(03)00202-7

[10] Gel'fand I. and Yaglom A. Calculation of amount of information about a random function contained in another such function. American Mathematical Society translations: Series 2. 1957; 12: 199-246.

[11] Arellano-Valle RB, Contreras-Reyes JE, & Genton MG (2012). Shannon entropy and mutual information for multivariate skew-elliptical distributions. Scandinavian Journal of Statistics, 40(1), 42–62. https://doi.org/10.1111/j.1467-9469.2011.00774.x

[12] Komaee, A. (2020). Mutual information rate between stationary Gaussian Processes. Results in Applied Mathematics, 7, 100107. https://doi.org/10.1016/j.rinam.2020.100107

[13] Algoet, Paul H.; Cover, Thomas M. (1988). "A Sandwich Proof of the Shannon-McMillan-Breiman Theorem" (PDF). The Annals of Probability. 16 (2): 899–909.

[14] Porta A, et al. (2014) Effect of age on complexity and causality of the cardiovascular control: Comparison between model-based and model-free approaches. PLoS ONE 9(2). doi:10.1371/journal.pone.0089463.

[15] Porta A, Faes L. Wiener–granger causality in network physiology with applications to cardiovascular control and Neuroscience. Proceedings of the IEEE. 2016;104(2):282-309. doi:10.1109/jproc.2015.2476824

[16] Porta A, et al. (2023) On the different abilities of cross-sample entropy and K-nearest-neighbor cross-unpredictability in assessing dynamic cardiorespiratory and cerebrovascular interactions. Entropy 25(4):599.

[17] Abarbanel HD, Carroll TA, Pecora LM, Sidorowich JJ, Tsimring LS (1994) Predicting physical variables in time-delay embedding. Physical Review E 49(3):1840–1853.

[18] Deshpande, G., Santhanam, P., & Hu, X. (2011). Instantaneous and Causal Connectivity in Resting State Brain Networks Derived From Functional MRI Data. NeuroImage, 54(2), 1043–1052. https://doi.org/10.1016/j.neuroimage.2010.09.024

[19] Ridderinkhof, K. R., Ullsperger, M., Crone, E. A., & Nieuwenhuis, S. (2004). The role of the medial frontal cortex in cognitive control. Science (New York, N.Y.), 306(5695), 443–447. https://doi.org/10.1126/science.1100301

[20] N. Packard, J. Crutchfield, D. Farmer and R. Shaw (1980). Geometry from a time series. Physical Review Letters. 45 (9): 712–716. Bibcode:1980PhRvL..45..712P. doi:10.1103/PhysRevLett.45.712.

[21] Whitney, H., Eells, J. and Toledo, D. (1992.) Collected Papers of Hassler Whitney, Boston: Birkhäuser, ISBN 0-8176-3560-2

[22] Lin FH, Ahveninen J, Raij T, et al. Increasing fMRI Sampling Rate Improves Granger Causality Estimates. Marinazzo D, ed. PLoS ONE. 2014;9(6):e100319. doi:https://doi.org/10.1371/journal.pone.0100319
